# The Minimal Proteome in the Reduced Mitochondrion of the Parasitic Protist *Giardia intestinalis*

**DOI:** 10.1371/journal.pone.0017285

**Published:** 2011-02-24

**Authors:** Petr L. Jedelský, Pavel Doležal, Petr Rada, Jan Pyrih, Ondřej Šmíd, Ivan Hrdý, Miroslava Šedinová, Michaela Marcinčiková, Lubomír Voleman, Andrew J. Perry, Neritza Campo Beltrán, Trevor Lithgow, Jan Tachezy

**Affiliations:** 1 Department of Parasitology, Faculty of Science, Charles University in Prague, Prague, Czech Republic; 2 Laboratory of Mass Spectrometry, Faculty of Science, Charles University in Prague, Prague, Czech Republic; 3 Department of Biochemistry & Molecular Biology, Monash University, Clayton Campus, Melbourne, Australia; Newcastle University, United Kingdom

## Abstract

The mitosomes of *Giardia intestinalis* are thought to be mitochondria highly-reduced in response to the oxygen-poor niche. We performed a quantitative proteomic assessment of *Giardia* mitosomes to increase understanding of the function and evolutionary origin of these enigmatic organelles. Mitosome-enriched fractions were obtained from cell homogenate using Optiprep gradient centrifugation. To distinguish mitosomal proteins from contamination, we used a quantitative shot-gun strategy based on isobaric tagging of peptides with iTRAQ and tandem mass spectrometry. Altogether, 638 proteins were identified in mitosome-enriched fractions. Of these, 139 proteins had iTRAQ ratio similar to that of the six known mitosomal markers. Proteins were selected for expression in *Giardia* to verify their cellular localizations and the mitosomal localization of 20 proteins was confirmed. These proteins include nine components of the FeS cluster assembly machinery, a novel diflavo-protein with NADPH reductase activity, a novel VAMP-associated protein, and a key component of the outer membrane protein translocase. None of the novel mitosomal proteins was predicted by previous genome analyses. The small proteome of the *Giardia* mitosome reflects the reduction in mitochondrial metabolism, which is limited to the FeS cluster assembly pathway, and a simplicity in the protein import pathway required for organelle biogenesis.

## Introduction

Mitochondria are eukaryotic organelles that are thought to have evolved from an alpha-proteobacterial endosymbiont about two billion years ago. The loss of bacterial autonomy and transition of the endosymbiont to a “protomitochondrion” were associated with a reduction in the number of genes in the endosymbiont genome; these genes were either transferred to the nuclear genome or lost. While the genome of the extant alpha-proteobacterium *Rickettsia prowazekii* contains 834 protein-coding genes [1], the largest number of genes (67 protein-coding genes) in a mitochondrial genome is found in *Reclinomonas americana* [2], with only three protein-coding genes present in the *Plasmodium falciparum* mitochondrial genome [3]. Paradoxically, the reduction of the mitochondrial genome did not lead to a reduction of the organellar proteome [4]. The acquisition of a mechanism for mitochondrial import at the earliest stage of the endosymbiont-to-protomitochondrion transition allowed the recruitment of the proteins of endosymbiotic origin that were now encoded in the nucleus, and the import of proteins of other origins [5]. Contemporary mitochondrial proteomes contain hundreds of proteins, up to 1100 proteins in the mouse [6].

Mitosomes are the most highly reduced forms of mitochondria, having completely lost their genomes and dramatically reduced their proteomes. Mitosomes have also lost many of the typical mitochondrial functions, such as respiration, the citric acid cycle, and ATP synthesis. Biosynthesis of FeS clusters is the only mitochondrial function seen to be retained by at least some mitosomes [7]. Mitosomes have become established independently in diverse groups of unicellular eukaryotes (protists); many of them once considered to be amitochondrial because they lack organelles with the expected mitochondrial morphology [8].

Organisms with mitosomes live under oxygen-limiting conditions, like the human intestinal parasites *Giardia intestinalis* [9] and *Entamoeba histolytica* [10], or are intracellular parasites like the microsporidians *Encephalitozoon cuniculi* and *Trachipleistophora hominis* [11], [12] and the apicomplexan *Cryptosporidium parvum* [13]. Mitosomes are tiny ovoid organelles enclosed by two membranes. Unlike mitochondria, the inner membrane of mitosomes does not form cristae. The morphology of the mitosome is reminiscent of the hydrogenosome, another form of mitochondrion that is present in some anaerobic protists, such as *Trichomonas vaginalis*. Unlike mitosomes, however, hydrogenosomes are metabolically active organelles that produce ATP by substrate level phosphorylation [14].

The limited knowledge of mitosomal proteomes has been gained mainly from analyses of genome sequences and localization studies of a few model mitosomal proteins [9], [11], [15]–[21]. The only published proteomics study that focused on mitosomes was that recently reported for the amoeba *E. histolytica*, identifying a unique sulfate activation pathway [22]. To increase our understanding of the function and origin of these enigmatic organelles, we established a large-scale proteomic approach to analyze the mitosomes of *Giardia intestinalis*. This organism was selected because *Giardia intestinalis* is a common human intestinal pathogen, its genome sequence has been published [23], [24], and it is considered to be among the most basal eukaryotes [25]. Moreover, previous analysis of the *G. intestinalis* genome provided little new information pertaining to the putative mitosomal proteome [24], so there are substantial gaps in our knowledge of the structure and function of this essential organelle. Here, we quantitatively analyzed the presence of isobarically-tagged proteins in mitosome enriched fractions. This technique allowed us to discriminate the mitosomal proteins from those of contaminating cellular structures. Combined with an exhaustive bioinformatics analysis, this strategy identified 139 putative mitosomal proteins; 20 of which were experimentally confirmed to be localized in mitosomes. Our results revealed that the proteome of the *G. intestinalis* mitosome is selectively reduced and houses a single metabolic pathway for FeS cluster assembly, a novel diflavin protein with NADPH reductase activity, a minimal protein import machinery and proteins that may be important for the interaction of mitosomes with other cellular compartments.

## Results and Discussion

### Identification of putative mitosomal proteins by isobaric tagging

**Figure 1:**
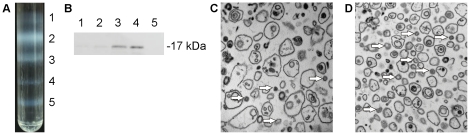
Isolation of mitosome-rich fractions. (A) Trophozoites were disrupted and centrifuged to remove unbroken cells, nuclei and cytoskeletal residue. The high-speed pellet was resuspended in sucrose buffer, layered onto an Optiprep density gradient, and centrifuged overnight. Five distinct fractions were obtained. (B) Fractions were collected and analyzed by SDS-PAGE and Western blot. The mitosomal marker GiIscU was detected in fractions #3 and #4 using a polyclonal rabbit antibody. (C–D) Electron microscopy of subcellular fractions. Fraction #3 (C) contains numerous vesicles of variable sizes, while fraction #4 (D) contains vesicles of more homogeneous sizes. Arrows indicate mitosomes.

**Figure 2:**
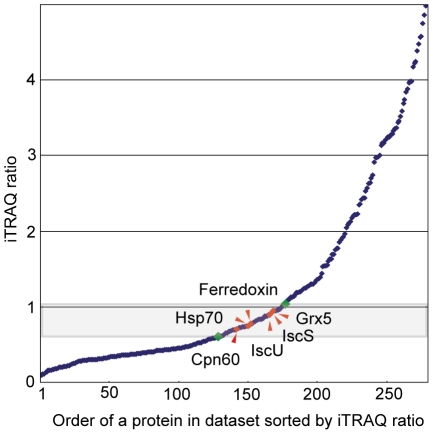
iTRAQ ratios define protein subcellular localization. Proteins in fractions #3 and #4 isolated on the Optiprep gradient were labeled with the iTRAQ-114 and iTRAQ-115 reagents, respectively, analyzed by LC MS/MS, and sorted according to the iTRAQ ratios. Mitosomal marker proteins (red diamonds) fall into a narrow range of iTRAQ ratios. Green diamondsdindicate the zone of proteins considered as mitosomal candidates (mitosomal distribution, MiD).

Mitosome-enriched fractions were separated from a *Giardia* homogenate by preparative centrifugation using a discontinuous Optiprep (iodixanol) gradient [26]. This method produced five dense organellar fractions (Fig. 1A). The mitosomal marker protein IscU was particularly enriched in fraction #4 and to a lesser extent in fraction #3 (Fig. 1B). Electron microscopy confirmed the presence of mitosomes in both fractions; however, co-fractionating vesicles of similar densities were also found (data not shown).To discriminate between putative mitosomal proteins and those of contaminating cellular structures, we compared the relative distribution of each protein in fractions #3 and #4. Because the mitosomal proteins necessarily co-fractionate (i.e. being contained within mitosomes) during gradient centrifugation, each of the *bona fide* mitosomal protein should display similar distribution ratios [27]. To this end, the proteins of fractions #3 and #4 were digested in parallel with trypsin and each peptide population was labeled with a distinct iTRAQ reagent and then combined. The isobaric mass characteristics of the iTRAQ reagents means the differentially-labeled peptides from fractions #3 and #4 form a single peak in the MS scan for protein identification. MS/MS analysis of the iTRAQ-labelled peptides liberates the isotope-encoded reporter ions, the ratio of which can reflects the distribution of the protein across the two fractions. In our analysis, the pooled peptides were analyzed by tandem mass spectrometry after subsequent separation with isoelectric focusing and nano-liquid chromatography (nano-LC MS/MS). The iTRAQ ratio was then calculated for each protein, and the proteins were sorted according to the relative distributions in the fractions (Fig. 2).

**Figure 3:**
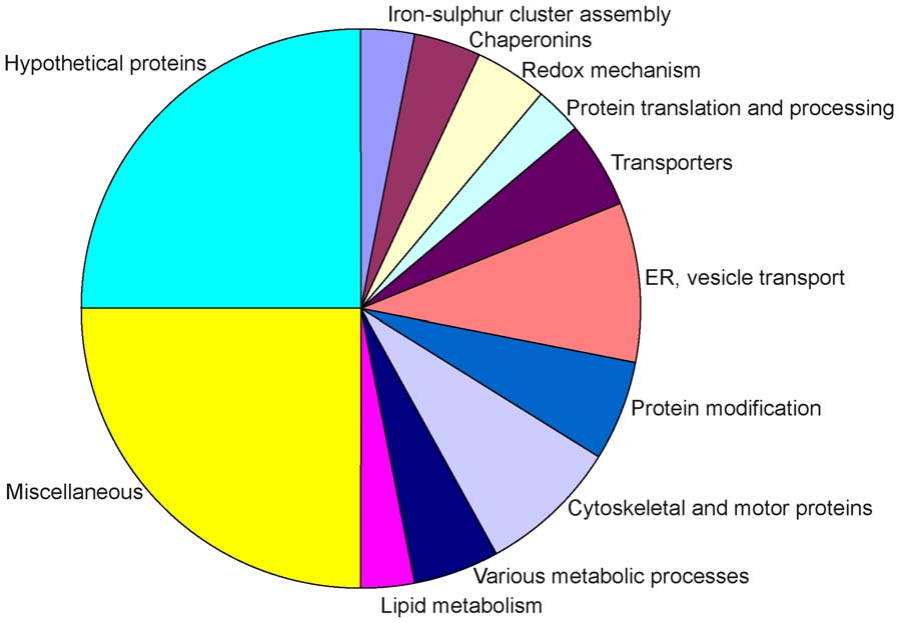
Classification of the identified proteins according to function. Functions were assigned based upon *Giardia*DB annotations, PSI-BLAST analysis and searches of the Pfam database (Tables 1, 2, 3, 4, 5, 6 and 7, Tables S2–S3).

Validating the methodology, mitosomal markers (IscS, IscU, [2Fe2S] ferredoxin, Cpn60, Hsp70 and glutaredoxin 5) [28] clustered together with similar iTRAQ ratios (Fig. 2). Proteins with ratios between the lowest and highest values for the markers were considered to be candidate mitosomal proteins. We also extended this window on both sides by half of the distance between the limiting markers and included all proteins in this extended window (Fig. 2). In total, we identified 638 proteins (Table S1), with 139 of these proteins meeting the defined criteria for mitosomal proteins (Tables 1–7). Each of the 139 mitosomal candidates was assigned to a probable function based on current annotations in the GiardiaDB, PSI BLAST searches in the NCBI nr database, and motif and domain searches in the Pfam database. Three additional bioinformatics tools were used to predict cellular localization (PsortII, TargetP 1.1 and SignalP 3.0), and two web-based programs were used to predict alpha-helical transmembrane region segments (TMHMM and Memsat3) (Tables S2–S4, summary is given in Tables 1–7). The candidate proteins were clustered into 13 groups according to their predicted functions (Tables 1–7, Fig. 3). The proteomic data confirmed the validity of 250 hypothetical genes predicted from the complete genome sequence of *Giardia* [24]; 40 of these formed the largest group of candidate mitosomal proteins.

**Table 1:** Putative mitosomal proteins classified by predicted function: Iron-sulfur cluster assembly, chaperones, redox mechanism and protein translocation and processing.

Accession number	Annotation	Identification		Localization					Structure		
		MASCOT	Coverage	MiD	SignalP	Target P	Psort II	Exp Ver.	MEMSAT3	SGP	TMHMM
		score	peptides				% mito		TM No.		TM No.
**Iron-sulfur cluster assembly**											
GL50803_14519	IscS, cysteine desulfurase	296	4	Y	N	O		M	0	#	0
GL50803_15196	IscU	243	5	Y	N	M	17%	M	0	#	0
EAA38809	Nfu	60	2	Y	N	M	39%	M	1		0
GL50803_14821	IscA	198	3	Y	N	O	35%	M	0	#	0
GL50803_2013	Glutaredoxin 5	249	3	Y	N	O	13%	M	0	#	0
**Molecular chaperones**											
GL50803_14581	mitochondrial type HSP70	404	7	Y	N	O	13%	M	0	#	0
GL50803_1376	GrpE	29	1	Y	N	M	39%	M	0	#	0
GL50803_17030	DnaJ protein, Jac1	*	*	*	N	O	35%	M	0	#	0
GL50803_9751	DnaJ protein, Type III	34	1	Y	N	O	13%	M	1		1
GL50803_103891	Cpn60	336	6	Y	N	O		M	0	#	0
GL50803_29500	Cpn10	68	1	Y	Y	O	9%	M	0	#	0
**Redox mechanism**											
GL50803_27266	[2Fe-2S] ferredoxin	182	2	Y	N	M	48%	M	0	#	0
GL50803_91252	GiOR-1, oxidoreductase	40	1	N	N	O	13%	M	0	#	0
GL50803_15897	GiOR-2, oxidoreductase	*	*	*	N	O	21%	O	0	#	0
GL50803_9827	Thioredoxin reductase	*	*	*	N	M	13%	O	0	#	0
GL50803_9719	NADH oxidase	271	5	Y	N	O	9%	**	0	#	0
GL50803_16076	Peroxiredoxin 1	293	5	Y	N	O	9%		0	#	0
**Protein translocation and processing**											
GL50803_17161	Tom40	208	2	Y	N	O	13%	M	0	#	0
XP_002364144	Pam18	68	1	Y	N	M	30%	M	0	#	0
GL50803_19230	Pam16	35	1	Y	N	O	13%	M	0	#	0
GL50803_9478	GPP, processing peptidase	30	1	Y	N	O	4%	M	0	#	0

Mascot score, Mascot total ion score for the identified protein. Coverage, number of unique peptides per identified protein. MiD, mitosomal distribution. Proteins are marked “Y” if their distributions in fractions #3 and #4 of the Optiprep gradient (measured by the iTRAQ ratio) were within the range between Cpn10 and IscU and the window that extended in both directions by half of the distance between these markers. Proteins with ratios outside of this range are indicated with “N”. TargetP and PsortII were used to predict the subcellular location of *Giardia* proteins. S, secretory; N, non-secretory; M, mitochondrial; O, other. Exp. ver., experimental verification of protein localization using the pONDRA expression vector. The recombinant tagged proteins were localized by fluorescence microscopy. M, mitosome; ER, endoplasmic reticulum; O, other; ? inconclusive. MEMSAT3 and TMHMM were used to predict transmembrane domains. SGP, predicted soluble proteins are marked with number sign (#). Asterisk (*) is used where no data were available. (**) transformed *Giardia* did not express the recombinant tagged protein.

**Table 2 pone-0017285-t002:** Putative mitosomal proteins classified by predicted function: transporters and proteins known to operate in endoplasmic reticulum and tramsport vesicles.

Accession number	Annotation	Identification		Localization					Structure		
		MASCOT	Coverage	MiD	SignalP	Target P	Psort II	Exp Ver.	MEMSAT3	SGP	TMHMM
		score	peptides				% mito		TM No.		TM No.
**Transporters**											
GL50803_114777	major facilitator superfamily mfs_1	658	8	N	N	M	4%	ER	10		12
GL50803_17296	major facilitator superfamily mfs_1	32	1	Y	N	M		**	7		10
GL50803_17342	major facilitator superfamily mfs_1	151	3	Y	N	M		**	10		12
GL50803_87446	ABC transporter, A family, putative	554	7	Y	N	O		**	4		7
GL50803_3470	ABC transporter, A family, putative	95	2	Y	N	M		**	6		7
GL50803_17165	ABC transporter, A family, putative	113	2	Y	N	O	4%		8		7
GL50803_21411	ABC transporter, A family, putative	429	10	Y	N	S			0		14
**ER, vesicle transport**											
GL50803_5744	Sec61-alpha	175	3	Y	N	M	22%	ER	10		9
GL50803_16906	Phosphatidate cytidylyltransferase	48	2	Y	N	M	9%	ER	7		8
GL50803_14200	Molybdenum cofactor sulfurase	56	1	Y	N	O	22%	ER	2		1
GL50803_14670	Protein disulfide isomerase PDI3	69	1	Y	Y	S	22%		1		0
GL50803_8064	Protein disulfide isomerase PDI5	58	1	Y	Y	S	13%	ER	1		1
GL50803_17121	ER Hsp70 (Bip)	1626	24	Y	Y	S	11%	ER	0	#	1
GL50803_15204	Endosomal cargo receptor 3	95	2	Y	Y	S			1		1
GL50803_14469	R-SNARE 3	45	1	Y	N	O			1		2
GL50803_8559	Vacuolar ATP synthase 16 kDa proteolipid subunit	90	1	Y	N	O	11%		4		4
GL50803_7532	Vacuolar ATP synthase catalytic subunit A	146	2	Y	N	O	17%		1		0
GL50803_13000	Vacuolar ATP synthase subunit d	342	5	Y	N	O	13%		1		0
GL50803_23833	Vacuolar protein sorting 35	26	1	Y	N	O	11%		1		0
GL50803_18470	Vacuolar proton-ATPase subunit, putative	608	8	Y	N	O	4%		6		6
GL50803_96670	Potassium-transporting ATPase alpha chain 1	473	9	Y	N	O	4%		10		8

Mascot score, Mascot total ion score for the identified protein. Coverage, number of unique peptides per identified protein. MiD, mitosomal distribution. Proteins are marked “Y” if their distributions in fractions #3 and #4 of the Optiprep gradient (measured by the iTRAQ ratio) were within the range between Cpn10 and IscU and the window that extended in both directions by half of the distance between these markers. Proteins with ratios outside of this range are indicated with “N”. TargetP and PsortII were used to predict the subcellular location of *Giardia* proteins. S, secretory; N, non-secretory; M, mitochondrial; O, other. Exp. ver., experimental verification of protein localization using the pONDRA expression vector. The recombinant tagged proteins were localized by fluorescence microscopy. M, mitosome; ER, endoplasmic reticulum; O, other; ? inconclusive. MEMSAT3 and TMHMM were used to predict transmembrane domains. SGP, predicted soluble proteins are marked with number sign (#). Asterisk (*) is used where no data were available. (**) transformed *Giardia* did not express the recombinant tagged protein.

**Table 3 pone-0017285-t003:** Putative mitosomal proteins classified by predicted function: protein modification, cztosceletal and motor proteins.

Accession number	Annotation	Identification		Localization				Structure		
		MASCOT	Coverage	MiD	SignalP	Target P	Psort II	MEMSAT3	SGP	TMHMM
		score	peptides				% mito	TM No.		TM No.
**Protein modification**										
GL50803_8587	Kinase, AGC NDR	22	1	Y	N	O	4%	0	#	0
GL50803_14223	Kinase, NEK	124	2	Y	N	O	13%	0	#	0
GL50803_16824	Kinase, NEK	87	2	Y	N	O		0	#	0
GL50803_17510	Kinase, NEK	25	1	Y	N	O	17%	0	#	0
GL50803_5375	Kinase, NEK	46	1	Y	N	O	17%	0	#	0
GL50803_11775	Kinase, NEK-frag	50	2	Y	N	O	17%	0	#	0
GL50803_7183	Kinase, NEK-frag	22	1	Y	N	O	13%	0	#	0
GL50803_8805	Kinase, SCY1	159	2	Y	N	O	11%	1		0
GL50803_7110	Ubiquitin	360	5	Y	N	O	17%	0	#	0
**Cytoskeletal and motor proteins**										
GL50803_11654	Alpha-1 giardin	934	17	Y	N	O	13%	1		0
GL50803_7796	Alpha-2 giardin	478	8	Y	N	O	17%	1		0
GL50803_5649	Alpha-10 giardin	294	5	Y	N	O	9%	1		0
GL50803_15097	Alpha-14 giardin	643	9	Y	N	O	4%	0	#	0
GL50803_112079	Alpha-tubulin	394	7	Y	N	O		0	#	0
GL50803_136020	Beta tubulin	841	13	Y	N	O		0	#	0
GL50803_42285	Ciliary dynein heavy chain 11	23	1	Y	N			1		0
GL50803_93736	Dynein heavy chain	29	1	Y	N		13%	0		0
GL50803_16993	FtsJ cell division protein, putative	24	1	Y	N	O	17%	0	#	0
GL50803_102101	Kinesin-3	85	1	Y	N	O	26%	0	#	0
GL50803_21444	Spindle pole protein, putative	63	2	Y	N	O	22%	0	#	0
GL50803_8589	Suppressor of actin 1	81	2	Y	N	O	11%	3		2

Mascot score, Mascot total ion score for the identified protein. Coverage, number of unique peptides per identified protein. MiD, mitosomal distribution. Proteins are marked “Y” if their distributions in fractions #3 and #4 of the Optiprep gradient (measured by the iTRAQ ratio) were within the range between Cpn10 and IscU and the window that extended in both directions by half of the distance between these markers. Proteins with ratios outside of this range are indicated with “N”. TargetP and PsortII were used to predict the subcellular location of *Giardia* proteins. S, secretory; N, non-secretory; M, mitochondrial; O, other. Exp. ver., experimental verification of protein localization using the pONDRA expression vector. The recombinant tagged proteins were localized by fluorescence microscopy. M, mitosome; ER, endoplasmic reticulum; O, other; ? inconclusive. MEMSAT3 and TMHMM were used to predict transmembrane domains. SGP, predicted soluble proteins are marked with number sign (#). Asterisk (*) is used where no data were available. (**) transformed *Giardia* did not express the recombinant tagged protein.

**Table 4 pone-0017285-t004:** Putative mitosomal proteins classified by predicted function: various metabolic processes, lipid metabolism.

Accession number	Annotation	Identification		Localization					Structure		
		MASCOT	Coverage	MiD	SignalP	Target P	Psort II	Exp Ver.	MEMSAT3	SGP	TMHMM
		score	peptides				% mito		TM No.		TM No.
**Various metabolic processes**											
GL50803_7203	Guanylate kinase	*	*	*	N	M	65%	O	0	#	0
GL50803_3287	Acetyl-CoA acetyltransferase	*	*	*	N	M	22%	O	0	#	0
GL50803_8163	Manganese-dependent inorganic pyrophosphatase, putative	25	1	Y	N	O	22%		0	#	0
GL50803_6497	Metal-dependent hydrolase	30	1	Y	N	O	13%		1		0
GL50803_10311	Ornithine carbamoyltransferase	665	8	Y	N	O	9%		1		0
GL50803_14993	Pyrophosphate-fructose 6-phosphate 1-phosphotransferase alpha subunit	56	1	Y	N	M	35%		1		0
GL50803_15380	CDC8 Thymidylate kinase	*	*	*	N	O	35%	O	0	#	0
**Lipid metabolism**											
GL50803_9062	Long chain fatty acid CoA ligase 5	279	3	Y	N	O	22%	**	0	#	0
GL50803_21118	Long chain fatty acid CoA ligase 5	25	1	Y	N	O	26%		0	#	0
GL50803_113892	Long chain fatty acid CoA ligase, putative	224	4	Y	N	O	26%		0	#	0
GL50803_7259	CDP-diacylglycerol-glycerol-3-phosphate 3-phosphatidyltransferase	43	1	N	N	M	22%		6		2

Mascot score, Mascot total ion score for the identified protein. Coverage, number of unique peptides per identified protein. MiD, mitosomal distribution. Proteins are marked “Y” if their distributions in fractions #3 and #4 of the Optiprep gradient (measured by the iTRAQ ratio) were within the range between Cpn10 and IscU and the window that extended in both directions by half of the distance between these markers. Proteins with ratios outside of this range are indicated with “N”. TargetP and PsortII were used to predict the subcellular location of *Giardia* proteins. S, secretory; N, non-secretory; M, mitochondrial; O, other. Exp. ver., experimental verification of protein localization using the pONDRA expression vector. The recombinant tagged proteins were localized by fluorescence microscopy. M, mitosome; ER, endoplasmic reticulum; O, other; ? inconclusive. MEMSAT3 and TMHMM were used to predict transmembrane domains. SGP, predicted soluble proteins are marked with number sign (#). Asterisk (*) is used where no data were available. (**) transformed *Giardia* did not express the recombinant tagged protein.

**Table 5 pone-0017285-t005:** Putative mitosomal proteins classified by predicted function: miscellaneous.

Accession number	Annotation	Identification		Localization					Structure		
		MASCOT	Coverage	MiD	SignalP	Target P	Psort II	Exp Ver.	MEMSAT3	SGP	TMHMM
		score	peptides				% mito		TM No.		TM No.
**Miscellaneous**											
GL50803_11953	Coatomer alpha subunit (WD40)	31	1	Y	N	O			0	#	0
GL50803_88765	Cytosolic HSP70	22	1	Y	N	O	4%		1		0
GL50803_112312	Elongation factor 1-alpha	424	10	Y	N	O	4%		1		0
GL50803_12102	Elongation factor 1-gamma	158	3	Y	N	M	13%		1		0
GL50803_28379	Multidrug resistance-associated protein 1	210	4	Y	N	O			0		10
GL50803_16313	Pescadillo (ribosome biogenesis)	52	1	Y	N	M	17%	**	0	#	0
GL50803_15380	CDC8 Thymidylate kinase	*	*	*	N	O	35%	O	0	#	0
GL50803_16354	Protein 21.1	25	1	Y	N	O	4%		0	#	0
GL50803_17288	Protein 21.1	54	2	Y	N	O	4%		0		0
GL50803_23492	Protein 21.1	130	1	Y	N	O	30%		1		0
GL50803_86855	Protein 21.1	22	1	Y	N	O	9%		0	#	0
GL50803_88245	Protein 21.1	23	1	Y	N	O	17%		0	#	0
GL50803_21662	Coiled-coil protein	31	1	N	N	M		**	0	#	0
GL50803_16152	Coiled-coil protein	57	2	Y	N	O			0	#	0
GL50803_8564	Coiled-coil protein	74	3	Y	N	O			0		0
GL50803_9515	Coiled-coil protein	61	2	Y	N	O			0	#	0
GL50803_40244	P24, putative	53	1	Y	N	O	13%		1		1
GL50803_6430	14-3-3 protein	78	2	Y	N	O	13%		1		0
GL50803_8903	Copine I	190	4	Y	N	O	44%	O	0	#	0
GL50803_14225	CXC-rich protein	494	8	Y	Y	S			0		1
GL50803_17476	CXC-rich protein	255	7	Y	Y	S	4%		0		1
GL50803_113656	Cysteine protease	73	2	Y	Y	S			1		1
GL50803_103454	High cysteine membrane protein Group 1	1038	14	Y	Y	S			1		1
GL50803_17328	High cysteine membrane protein Group 2	113	3	Y	Y	S			0		1
GL50803_91099	High cysteine membrane protein Group 2	65	1	Y	Y	S	13%		0	#	1
GL50803_114042	High cysteine membrane protein Group 4	330	5	Y	Y	S			1		1
GL50803_11359	Ribosomal protein S4	31	1	Y	N	O	17%		1		0
GL50803_17411	TCP-1 chaperonin subunit gamma	24	1	Y	N	O			1		0

Mascot score, Mascot total ion score for the identified protein. Coverage, number of unique peptides per identified protein. MiD, mitosomal distribution. Proteins are marked “Y” if their distributions in fractions #3 and #4 of the Optiprep gradient (measured by the iTRAQ ratio) were within the range between Cpn10 and IscU and the window that extended in both directions by half of the distance between these markers. Proteins with ratios outside of this range are indicated with “N”. TargetP and PsortII were used to predict the subcellular location of *Giardia* proteins. S, secretory; N, non-secretory; M, mitochondrial; O, other. Exp. ver., experimental verification of protein localization using the pONDRA expression vector. The recombinant tagged proteins were localized by fluorescence microscopy. M, mitosome; ER, endoplasmic reticulum; O, other; ? inconclusive. MEMSAT3 and TMHMM were used to predict transmembrane domains. SGP, predicted soluble proteins are marked with number sign (#). Asterisk (*) is used where no data were available. (**) transformed *Giardia* did not express the recombinant tagged protein.

**Table 6 pone-0017285-t006:** Putative mitosomal proteins classified by predicted function: miscellaneous - continued; hypothetical proteins.

Accession number	Annotation	Identification		Localization					Structure		
		MASCOT	Coverage	MiD	SignalP	Target P	Psort II	Exp Ver.	MEMSAT3	SGP	TMHMM
		score	peptides				% mito		TM No.		TM No.
GL50803_10330	Tenascin precursor	330	4	Y	Y	S	11%		0	#	0
GL50803_16477	Tenascin-37	178	4	Y	Y	S	17%		1		0
GL50803_16833	Tenascin-like	96	2	Y	Y	S			0	#	0
GL50803_13561	Translation elongation factor	36	1	Y	N	O	13%		1		0
GL50803_15889	UDP-N-acetylglucosamine-dolichyl-phosphateN-acetylglucosamine- phosphotransferase	36	1	Y	Y	S	4%		10		7
GL50803_11521	VSP	198	3	Y	Y	S			1		1
GL50803_137618	VSP	530	9	Y	N	O	4%		2		1
GL50803_11470	VSP with INR	220	3	Y	N	O			2		1
GL50803_6733	Zinc finger domain	55	1	Y	N	S	22%		4		4
**Hypothetical proteins**											
GL50803_12999	Hypothetical protein	414	5	Y	Y	M		?	2		2
GL50803_14939	Hypothetical protein	133	2	Y	Y	M	30%	M	1		2
GL50803_15985	Hypothetical protein (VAP, VAMP associated protein)	35	1	Y	N	M	13%	M	1		1
GL50803_16596	Hypothetical protein	177	3	N	N	M	30%	O	0	#	0
GL50803_4768	Hypothetical protein	21	1	Y	N	M	57%	O	0	#	0
GL50803_9296	Hypothetical protein	178	4	Y	Y	M	57%	M	0	#	0
GL50803_11237	Hypothetical protein	24	1	Y	N	O	9%		1		0
GL50803_11557	Hypothetical protein	41	1	Y	N	O	17%		1		0
GL50803_11866	Hypothetical protein	25	1	Y	N	O	22%		0	#	0
GL50803_13288	Hypothetical protein	35	1	Y	N	O	9%		1		0
GL50803_13413	Hypothetical protein	95	2	Y	N	O	11%		2		2
GL50803_137685	Hypothetical protein	200	4	Y	N	S			13		9
GL50803_137746	Hypothetical protein	25	1	Y	N	O			0	#	0
GL50803_13922	Hypothetical protein	1121	14	Y	Y	S			1		1
GL50803_14164	Hypothetical protein	23	1	Y	N	O	13%		0	#	0
GL50803_14278	Hypothetical protein	31	1	Y	N	O	13%		0	#	0
GL50803_14660	Hypothetical protein	105	2	Y	N	O	35%		1		0

Mascot score, Mascot total ion score for the identified protein. Coverage, number of unique peptides per identified protein. MiD, mitosomal distribution. Proteins are marked “Y” if their distributions in fractions #3 and #4 of the Optiprep gradient (measured by the iTRAQ ratio) were within the range between Cpn10 and IscU and the window that extended in both directions by half of the distance between these markers. Proteins with ratios outside of this range are indicated with “N”. TargetP and PsortII were used to predict the subcellular location of *Giardia* proteins. S, secretory; N, non-secretory; M, mitochondrial; O, other. Exp. ver., experimental verification of protein localization using the pONDRA expression vector. The recombinant tagged proteins were localized by fluorescence microscopy. M, mitosome; ER, endoplasmic reticulum; O, other; ? inconclusive. MEMSAT3 and TMHMM were used to predict transmembrane domains. SGP, predicted soluble proteins are marked with number sign (#). Asterisk (*) is used where no data were available. (**) transformed *Giardia* did not express the recombinant tagged protein.

**Table 7:** Putative mitosomal proteins classified by predicted function: hypothetical proteins – continued.

Accession number	Annotation	Identification		Localization				Structure		
		MASCOT	Coverage	MiD	SignalP	Target P	Psort II	MEMSAT3	SGP	TMHMM
		score	peptides				% mito	TM No.		TM No.
GL50803_14845	Hypothetical protein	69	2	Y	N	O	4%	0	#	0
GL50803_15084	Hypothetical protein	22	1	Y	N	O		0	#	0
GL50803_16424	Hypothetical protein	117	3	Y	N	O	26.1%	0	#	0
GL50803_16430	Hypothetical protein	32	1	Y	N	O	9%	1		0
GL50803_16998	Hypothetical protein	24	1	Y	N	O	17%	0	#	0
GL50803_17236	Hypothetical protein	69	1	Y	N	M		10		10
GL50803_1937	Hypothetical protein	75	2	Y	N	S		2		2
GL50803_23389	Hypothetical protein	33	1	Y	N	O		4		6
GL50803_28962	Hypothetical protein	39	1	Y	Y	S	4%	1		1
GL50803_29327	Hypothetical protein	111	2	Y	N	O	17%	1		0
GL50803_3021	Hypothetical protein	21	1	Y	N	O	13%	0	#	0
GL50803_32999	Hypothetical protein	98	3	Y	N	O	13%	0	#	0
GL50803_3491	Hypothetical protein	25	1	Y	N	O	30%	1		0
GL50803_6617	Hypothetical protein	350	5	Y	Y	S		1		1
GL50803_7188	Hypothetical protein	926	11	Y	Y	S	13%	3		1
GL50803_7242	Hypothetical protein	69	1	Y	N	O	22%	3		3
GL50803_7244	Hypothetical protein	144	3	Y	N	O	11%	4		3
GL50803_94658	Hypothetical protein	27	1	Y	N	O	13%	0	#	0
GL50803_9503	Hypothetical protein	206	3	Y	N	O	9%	0	#	0
GL50803_9780	Hypothetical protein	333	5	Y	Y	S	11%	0	#	0
GL50803_9861	Hypothetical protein	137	2	Y	N	O	4%	0	#	0
GL50803_10016	Hypothetical protein	265	5	Y	Y	S	22%	1		0
GL50803_111809	Hypothetical protein	34	1	Y	N	O		0	#	0

Mascot score, Mascot total ion score for the identified protein. Coverage, number of unique peptides per identified protein. MiD, mitosomal distribution. Proteins are marked “Y” if their distributions in fractions #3 and #4 of the Optiprep gradient (measured by the iTRAQ ratio) were within the range between Cpn10 and IscU and the window that extended in both directions by half of the distance between these markers. Proteins with ratios outside of this range are indicated with “N”. TargetP and PsortII were used to predict the subcellular location of *Giardia* proteins. S, secretory; N, non-secretory; M, mitochondrial; O, other. Exp. ver., experimental verification of protein localization using the pONDRA expression vector. The recombinant tagged proteins were localized by fluorescence microscopy. M, mitosome; ER, endoplasmic reticulum; O, other; ? inconclusive. MEMSAT3 and TMHMM were used to predict transmembrane domains. SGP, predicted soluble proteins are marked with number sign (#). Asterisk (*) is used where no data were available. (**) transformed *Giardia* did not express the recombinant tagged protein.

### Evolution-inspired orthology phylogenetic profiling

Previous phylogenetic analyses of known mitosomal proteins have generally confirmed their alpha-proteobacterial origin [28]–[30]. On this premise, we compared the genomes of *G. intestinalis* and *Rickettsia typhi* using the orthology phylogenetic profile tool at GiardiaDB (http://www.orthomcl.org/cgi-bin/OrthoMclWeb.cgi) to identify proteins of alpha-proteobacterial ancestry in the *G. intestinalis* genome. The phylogenetic profiling yielded 106 candidate genes that were analyzed with the topology prediction algorithms described above (Table S5). Based on these analyses, six additional proteins: acetyl CoA acetyl transferase, CDP-diacylglycerol-glycerol-3-phosphate 3-phosphatidyltransferase, guanylate kinase, J-protein HesB, thioredoxin reductase, and thymidylate kinase were added to the set of candidate mitosomal proteins identified by our proteomics approach (Tables 1–7).

### Experimental validation of protein subcellular localization

**Figure 4:**
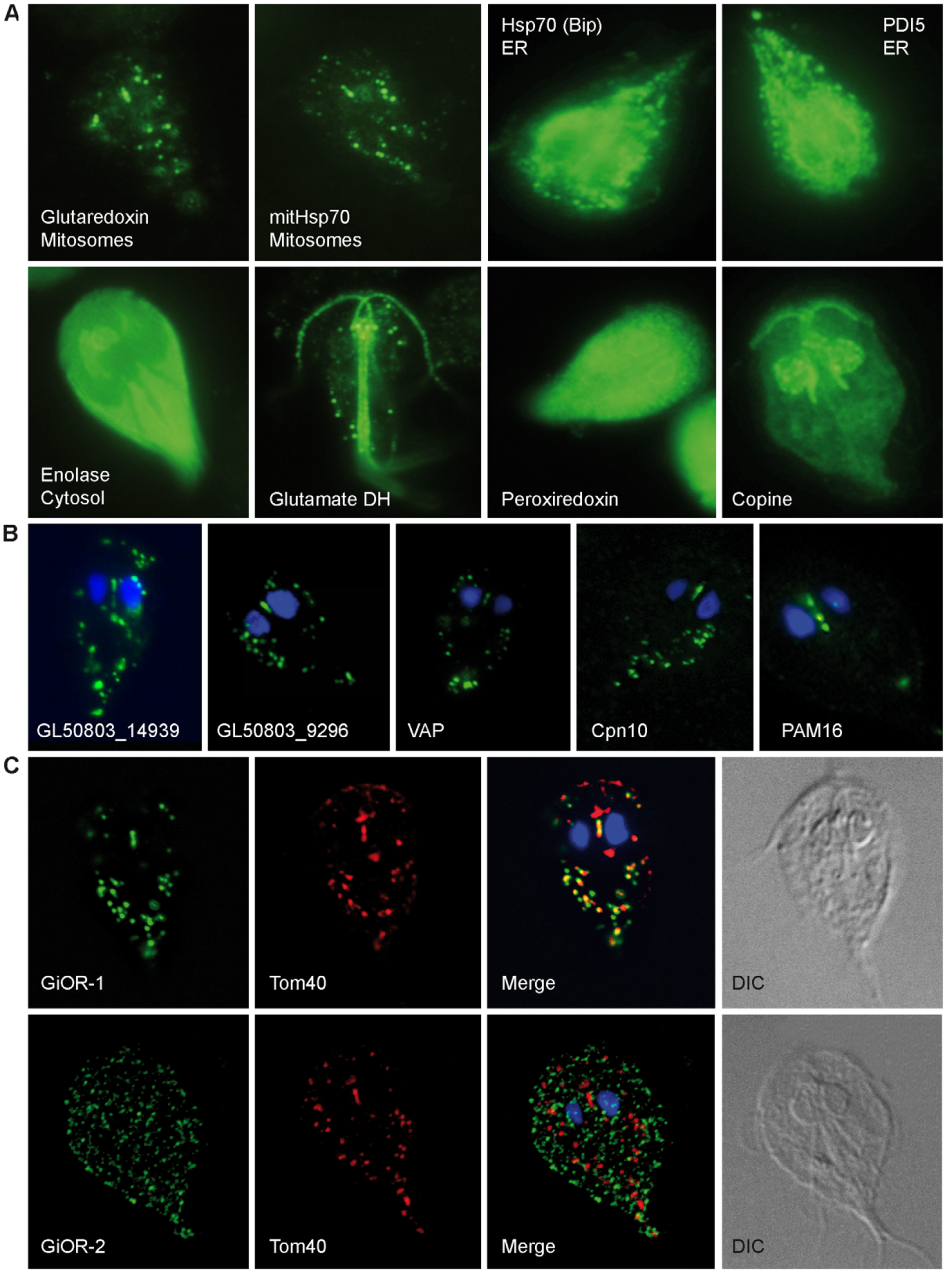
Sub-cellular localization of selected proteins in *Giardia*. Transformed *G. intestinalis* cells with episomally-expressed HA-tagged proteins. (A) Marker proteins were stained using a mouse anti-HA antibody (green). Grx5, glutaredoxin 5; ER, endoplasmic reticulum; glutamate DH, glutamate dehydrogenase. (B) Predicted mitosomal proteins (GL50803_14939, GL50803_9296, VAP, Cpn10, Pam16) were stained using a mouse anti-HA antibody (green). (C) Cellular localization of tagged diflavin proteins GiOR-1 and GiOR-2 stained with mouse anti HA antibody (green). Tom40 was detected by polyclonal rabbit anti-Tom40 antibody (red).

The cellular localization of the selected candidate proteins was observed by stable episomal expression in *Giardia*. To establish the morphology of subcellular localizations by this approach, we first observed the localization of five marker proteins: cytosolic enolase, two proteins from the endoplasmic reticulum (Hsp70 and protein disulfide isomerase 5), mitosomal Hsp70 and glutaredoxin (Fig. 4A). We added to these markers of known location, three proteins of untested location with iTRAQ ratios outside that of the mitosomal range: glutamate dehydrogenase, copine and peroxiredoxin. Glutamate dehydrogenase and copine were associated with cytoskeletal structures, while peroxiredoxin localizes to the endoplasmic reticulum network. This strategy was used to test the sub-cellular localization of 44 selected proteins. Of these 20 expressed fluorescent fusions that were found in the mitosomes (Tables 1–7). By way of example, four of these: VAP, Pam16, Cpn10 and unknown proteins GL50803_9296 and GL50803_ 14939 are shown in Fig. 4B.

### Iron-sulfur cluster assembly

Proteins involved in FeS cluster assembly formed the most prominent functional group within the predicted mitosomal proteins. These included components required for the formation of transient FeS clusters on the molecular scaffold (IscS, IscU, Nfu) (Fig. S1) and components that have been proposed to transfer the transient FeS clusters to target apoproteins, including IscA (Fig. S2), the monothiol glutaredoxin 5, chaperone Hsp70 and its co-chaperones the J-protein HscB (Fig. S3) and nucleotide exchange factor GrpE (Fig. S4). The identification of the FeS cluster assembly machinery in the mitosomal proteome is consistent with the ability of the mitosome-enriched fraction to catalyze the formation of FeS clusters on a ferredoxin apoprotein [9]. However, when we compared the FeS cluster machinery of *Giardia* mitosomes to that of *S. cerevisiae* and *Trypanosoma brucei* mitochondria, we found that several mitochondrial components were absent from the mitosomes (Table 8).

**Table 8:** Comparison of iron-sulfur cluster assembly machineries in organisms with mitosomes (*Giardia intestinalis*, *Cryptosporidium parvum*, and *Encephalitozoon cuniculi*), hydrogenosomes (*Trichomonas vaginalis*), and mitochondria (*Trypanosoma brucei*, *Saccharomyces cerevisiae*).

Name	*G. intestinalis*	*C. parvum*	*E. cuniculi*	*T. vaginalis*	*T. brucei*	*S. cerevisiae*
IscS (Nfs)	**•**	**•**	**•**	**••**	**•**	**•**
Isd11	**○**	**○**	**•**	**••**	**•••**	**•**
Nfu	**•**	**○**	**○**	**•••**	**•••**	**•**
IscU (Isu)	**•**	**•**	**•**	**•**	**•**	**••**
IscA1(Isa1)	**○**	**○**	**○**	**○**	**•**	**•**
IscA2 (Isa2)	**•**	**○**	**○**	**••••**	**•**	**•**
Iba57	**○**	**○**	**○**	**○**	**•**	**•**
Ind	**○**	**○**	**○**	**•••**	**•**	**•**
Grx5	**•**	**○**	**•**	**○**	**•**	**•**
Ferredoxin (Yah1)	**•**	**•**	**•**	**•••••••**	**••**	**•**
FOR (Arh1)	**○**	**•**	**•**	**○**	**•**	**•**
Frataxin (Yfh1)	**○**	**•**	**•**	**••**	**•**	**•**
HSP70	**•**	**•**	**•**	**•••**	**••**	**•♦♦**
Dna-J (Jac1)	**•**	**•**	**•**	**••**	**•**	**•**
GrpE	**•**	**•**	**•**	**••**	**•**	**•**
Atm1	**○**	**•**	**•**	**○**	**•**	**•**
Erv1	**○**	**○**	**•**	**○**	**•**	**•**

Filled circles indicate the presence of protein exhibiting homology to the known component of mitochondrial iron-sulfur cluster assembly machinery identified by BLAST searches. Empty circles indicates absence of homologous protein. Mitochondria of *S. cerevisiae* possess three distinct Hsp70 of which Ssq1 is devoted for FeS cluster assembly (filled circle), while Ssc1, and Ecm10 have other fuctions (diamonds). Other eukaryotes possess multifunctional Hsp70. IscS, cysteine desulfurase; Isd11, IscS binding protein; Nfu, IscU, IscA, a scafold proteins; Iba57, IscA binding protein required for [4Fe4S] cluster assembly; Ind, P-loop NTPaseb required for assembly of respiratory complex I; Grx5, glutaredoxin 5; ferredoxin, [2Fe2S] ferredoxin that transport electrons; FOR, ferredoxin oxidoreductase; frataxin, iron binding protein; Hsp70, chaperone; DnaJ, GrpE, co-chaperones; Atm1, ABC half trasnporter; Erv1, sulfhydryl oxidase. Names of proteins used for *S. cerevisiae* orthologs are in brackets.

A striking deviation from other eukaryotes is the absence of frataxin in *Giardia* mitosomes. Frataxin is invariably present in eukaryotes that contain the ISC-type FeS cluster assembly machinery. The presence of frataxin in mitosomes was found in *E. cuniculi* [17], and genes encoding frataxin are present in the genomes of *C. parvum* and the diplomonad *Spironucleus vortens*, a close relative of *Giardia*. We failed to identify frataxin in the genomes of three *G. intestinalis* strains in the GiardiaDB, using either BLAST searches or the motif search tool.

Two IscA-like proteins, IscA1 (Isa1) and IscA2 (Isa2) are present in virtually all mitochondria [31] and are thought to act as scaffold proteins for transient FeS clusters [32]–[34] and/or serve as iron donors [35]. Interestingly, the *Giardia* mitosome contains only a single IscA-2 type protein (Fig. S2), while IscA-1 is absent. The same situation was found in hydrogenosomes of *Trichomonas vaginalis* (Table 8). No genes encoding IscA were found in the genomes of other organisms with mitosomes. The observed distributions of IscA therefore suggest that IscA-1 was lost in mitosomes and hydrogenosomes together with a specific set of mitochondrial FeS proteins, while IscA-2 was retained in *Giardia* mitosomes to function either in the maturation of specific FeS protein(s) or as an iron transporter [35].

The mitosomes did not contain Ind1 or Iba57. In mitochondria, these proteins are required for the formation of FeS clusters on specific substrates. Ind1 is a P-loop NTPase that is required for the maturation of FeS proteins of the multi-subunit respiratory complex I [36], [37]. Homologues of Ind1 are also present in the hydrogenosomes of *T. vaginalis* (Table 8), which contain a highly reduced form of complex I with only two FeS catalytic subunits [38]. The selective absence of Ind1 in the mitosomes of *Giardia* (Table 8) is thus consistent with the absence of complex I and highlights the specific role of Ind1 in the biogenesis of this respiratory complex. Iba57 forms a complex with the scaffold protein IscA (Isa1p and Isa2p in yeast), which plays a specific role in [4Fe4S] cluster assembly of aconitase-type proteins and the functional activation of mitochondrial radical-SAM FeS proteins [39]. As in the case of Ind1, the absence of Iba57 likely reflects the absence of the respective substrate proteins in mitosomes.

### Pyridine nucleotide-driven electron transport in mitosomes

The formation of FeS clusters requires reducing equivalents, which are provided by a short electron chain consisting of the [2Fe2S] ferredoxin and ferredoxin:NADP^+^ reductase (FNR) [40]. The presence of this chain has been predicted in the mitosomes of *C. parvum* and *E. cuniculi*; however, [2Fe2S] ferredoxin, but not FNR, was found in *Giardia* mitosomes (Table 1). We identified a distinct protein with a possible redox activity named GiOR-1 (GL50803_91252), which is currently annotated in the GiardiaDB as an inducible nitric oxide synthase. This protein consists of a flavodoxin-like FMN-binding domain that is connected to a cytochrome p450 reductase-like domain, including a FAD binding pocket and an NADP(H) binding site (Fig. S5). These two domains are present in the C-termini of various oxidoreductases, such as cytochrome p450 reductase and nitric oxide synthase, and serve as electron donors (Fig. S5). GiOR-1 does not contain an N-terminal domain that determines the specific functions of known oxidoreductases.

The architecture of GiOR-1 resembles that of the recently identified protein Tah18 in *Saccharomyces cerevisiae* [41], [42]. Tah18 was shown to form a complex with Dre2 in the cytosol, where it participates in cytosolic FeS cluster assembly [43]. Under oxidative stress, the Dre2-Tag18 complex was destabilized, and Tah18 relocalized from the cytosol to the mitochondria. This behavior has been shown to be associated with apoptotic events. Searches for a Dre2 homologue in *Giardia* were unsuccessful. However, we identified a second paralogue of Tah18 named GiOR-2 (GL50803_15897, Fig. S5). The expression of tagged GiOR-1 and GiOR-2 in *G. intestinalis* confirmed that the GiOR-1 is localized to the mitosome, but GiOR-2 was found in numerous vesicles that did not correspond to mitosomes (Fig. 4C). To assess the oxidoreductase activity of GiOR-1, recombinant GiOR-1 was produced in *Escherichia coli* and isolated as a yellow protein, which is expected for diflavin oxidoreductases. GiOR-1 efficiently transferred electrons from NADPH to dichlorophenolindolphenol, whereas an about 30 fold lower activity was measured using NADH as the electron donor (Table 9). Low specific activities were observed also with methyl viologen and oxygen as electron acceptors (Table 9). No activity was observed when GiOR-1 was assayed with *G. intestinalis* mitosomal ferredoxin as a possible native electron acceptor. These results suggest that GiOR-1 does not act directly as a ferredoxin reductase in mitosomes, however, its ability to utilize NADPH as an electron donor indicates that pyridine nucleotides are involved in mitosomal electron transport.

**Table 9:** Activity of mitosomal diflavin oxidoreductase GiOR-1.

Substrate	Specific activity [µg.min^−1^.mg^−1^]	Standard deviation
NADPH	0	
NADPH+DCIP	9,053	0,111
NADH + DCIP	0,269	0,034
NADPH + MV	0,450	0,205
NADPH + O_2_	0,144	0,042
NADPH+ferredoxin	0	

Electron donors: NADPH, NADH.

Electron acceptors: DCIP, dichlorophenol*-*indolephenol; MV, methyl viologen; O_2_, aerobic conditions; ferredoxin, recombinant *G. intestinalis* [2Fe2S]ferredoxin.

### Molecular chaperones in the mitosomal matrix: protein folding and assembly

A single mitosomal Hsp70, three J-protein co-chaperones and the nucleotide exchange factor GrpE were identified in the mitosomes. The J-proteins included HscB, an orthologue of yeast Jac1 (Fig. S3)that has a predicted role in FeS cluster biogenesis [44], and Pam18/Tim14, which is required for translocation of proteins across the mitochondrial inner membrane [45]. The third J-protein also contains an N-terminal DnaJ domain (type III family); however, its function cannot be inferred from domain structure or phylogenetic profiling. We also identified the chaperonins Cpn60 and Cpn10 (Fig. S6), that function in folding and assembly of newly-imported proteins [46], [47] (Table 1).

### Protein import

We identified four components that are potentially involved in transporting proteins across the mitosomal membranes: a homologue of a mitochondrial Tom40, which would form a general import pore in the outer mitosomal membrane, and the three essential components of the PAM (presequence translocase-associated motor) complex: Pam18, Pam16 (Fig. S7) and mHsp70. Pam18 and Pam16 form an intimate complex that anchors a population of the matrix Hsp70 to the inner membrane and regulates its activity to drive protein translocation across the inner membrane [48]. Typically, it functions together with a TIM complex that forms the translocation pore for protein passage across the membrane. In representative organisms from all lineages of eukaryotes, the TIM complex is built from one or two proteins of the Tim17/22/23 family [49]. Surprisingly, we find no evidence for a member of this protein in our proteomics data, and sensitive hidden Markov model searches detected no related sequences in the *Giardia* genome (unpublished, see Methods). In eukaryotes, the Sec61 channel catalyzes protein transport across the endoplasmic reticulum [2], while a highly-related protein called SecY is the translocation channel in the inner membrane of bacteria, including the alpha-proteobacteria from which mitochondria are derived. Interestingly, *Reclinomonas americana* encodes a bacterial-type SecY protein translocation channel in its mitochondrial genome [50], and our proteomics analysis detected what appeared to be contamination of the mitosomal membranes with GiSecY/Sec61. We expressed a tagged version of this protein in *Giardia* but it localized to the endoplasmic reticulum, as expected for a cognate Sec61, rather than to the mitosomes. The nature of the mitosomal inner membrane protein translocation channel remains unknown, and yet must exist given that at least 17 of the proteins detected in the mitosomal proteome are likely to reside in the matrix.

We suggest that Tim23/17/22 protein(s) have been secondarily lost from *Giardia*, given that the these proteins appear to be derived from components of the ancestral endosymbiont [48] and are present in all other groups of eukaryotes including other members of the Excavata [51], particularly *T. vaginalis* (TrichDB, http://trichdb.org/trichdb; our unpublished data). Because there is evidence to suggest that *T. vaginalis* and *G. intestinalis* share a common ancestor [44], [52], the absence of a Tim23 homologue in *Giardia* likely reflects the overall simplification of the organelle than a primitive trait. Why has the TIM complex been replaced? In addition to a reliance on ATP hydrolysis mediated by the PAM motor, the TIM complex is powered by the membrane potential through its physical association with the respiratory complexes III and IV [53], [54]. *Giardia* mitosomes do not generate a large membrane potential, as shown by their inability to accumulate the routinely used mitochondrial probes that are sensitive to the membrane potential (e.g., mitotrackers, JC-1, our observations). Perhaps any membrane potential that is present, is insufficient to support the function of a TIM23 translocase.

### Interaction of mitosomes with other cellular compartments

In the *Giardia* mitosomes, we identified a VAMP (vesicle-associated membrane protein)-associated protein, VAP (Table 6). VAPs are involved mainly in membrane trafficking and lipid metabolism. They provide membrane anchors for various lipid binding proteins on the surfaces of the endoplasmic reticulum and Golgi complex [53] and physically interact with SNARE proteins, with FFAT-motif containing lipid transport proteins and microtubules. Like other VAPs, the *Giardia* VAP protein contains an N-terminal domain that includes the VAP consensus sequence [55], a central coiled-coil domain and a C-terminal transmembrane domain with the putative dimerization motif GxxxG (Fig. S8). The presence of a VAP protein has not been reported in mitochondria or other mitosomes so far. In *Giardia*, GiVAP was found within the set of hypothetical proteins with distribution value corresponding to mitosomal proteins (Table 6) and its mitosomal localization was experimentally confirmed (Fig. 4B).

### Hypothetical proteins

The set of mitosomal candidates contains 40 proteins annotated as hypothetical proteins. We selected six proteins with high mitochondrial score (Tables 6–7) for the verification of their sub-cellular localization. Three proteins were confirmed to reside in mitosomes (Table 6, Fig 4): (i) putative VAP (GL50803_15985) that is discussed above, (ii) hypothetical protein GL50803_14939 that contains two predicted transmembrane domains (residues 13–35 and 102–124), and (iii) a putative soluble globular protein GL50803_9296. The latter two proteins seem to be unique for giardia as no orthologues were identified in available databases. Two other hypothetical proteins (GL50803_16596 and GL50803_4768) were observed in the cytosol and in association with kinetosomes, respectively (Table 6, data not shown). The cellular localization of hypothetical protein GL50803_12999 remains inconclusive. Although the protein co-localized with IscU in some vesicles, it was not observed in typical rod-like structure between nuclei (data not shown).

### Origin of mitosomes and perspectives

Mitosomes are thought to have evolved several times in different eukaryotic lineages through the reduction of ancestral mitochondria. For example, microsporidians are intracellular parasites allied with Fungi; whereas Fungi typically possess fully equipped mitochondria with large proteomes (>850 proteins) [11], [56], only twenty to thirty proteins have been identified from genome analysis of *E. cuniculi* as having similarity to *bona fide* mitochondrial proteins of *Saccharomyces cerevisiae* [15], [18], [21]. Apicomplexan parasites related to *Plasmodium* also include organisms with mitosomes, such as *Cryptosporidium parvum* and *Cryptosporidium hominis*. Based on genomic analyses, 37–54 proteins have been predicted to reside in these mitosomes [19], of which 18 were detected by mass spectrometry in whole *C. parvum* sporozoites [25].

**Figure 5:**
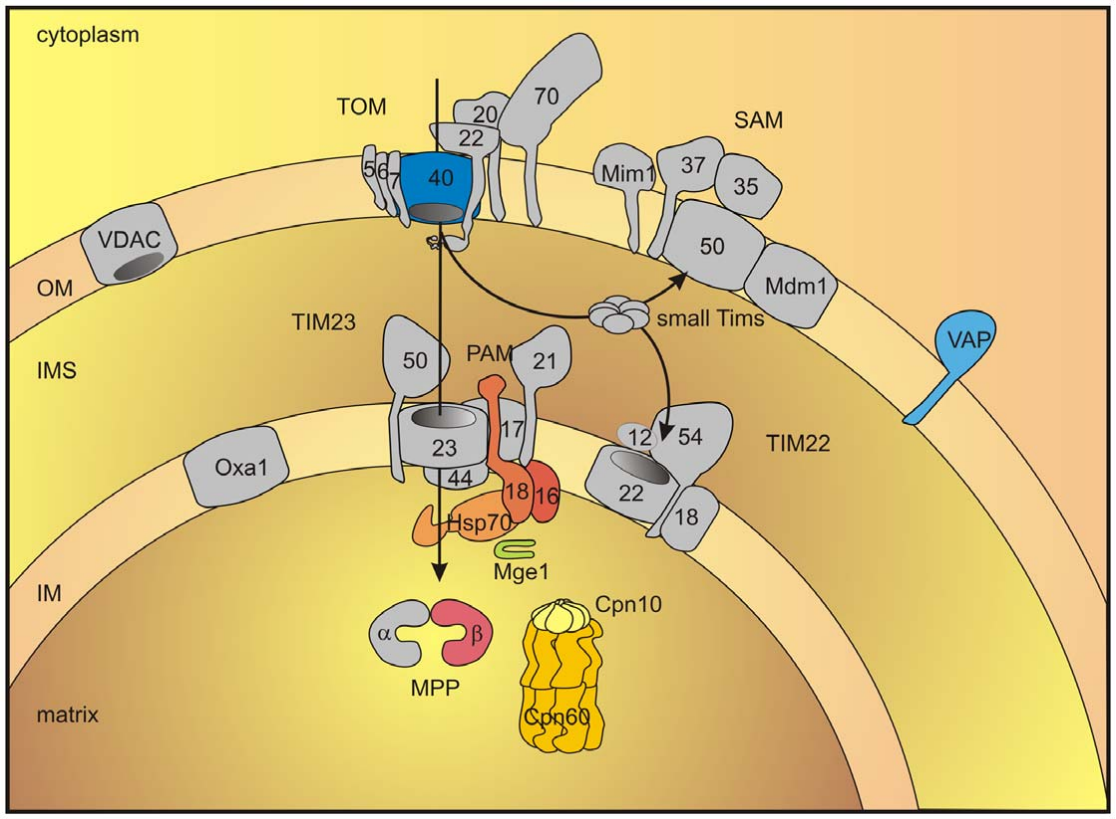
Schematic representation of protein import pathway in the mitosome of *G. intestinalis.* Components identified in mitosome are highlighted by color. Components that are known to participate in the protein import into mitochondria of animals and fungi are shown in grey colour. OM, outer membrane; IMS, intermembrane space; IM, inner membrane; TOM, translocase of outer membrane; SAM, sorting and assembly machinery; TIM, translocase of inner membrane; PAM, presequence translocase-associated motor; VAP, VAMP (vesicle-associated membrane protein)-associated protein; VDAC, voltage-dependent anion channel; MPP, mitochondrial processing peptidase

An intriguing question concerns the nature of the mitochondrial progenitor from which mitosomes of *G. intestinalis* have evolved. *Giardia* is a member of the Excavate group, which has recently been re-considered to belong to the basal groups of eukaryotes based on its mechanism of cytochrome c and c1 biogenesis [25], [57]. These and other data have placed the root of eukaryotes between Excavata and Euglenozoa, a group of protists that includes trypanosomatids [58]. In this respect, there is an apparent simplicity in the protein import machinery of the *Giardia* mitosomes that deserves attention (Fig. 5). The proteomics analysis detected in mitosomes a protein recently shown to be Tom40, the protein translocation channel across the outer membrane [25], [59]. The current model for the evolution of the TOM complex posits that Tom40 was derived from a beta-barrel protein in the endosymbiont's outer membrane, perhaps of an usher or autotransporter type protein translocase [60]. Because two other proteins: Tom7 and Tom22, have been found in representative species of all other eukaryotic groups [58], the model further suggests that the first TOM complex was composed of Tom40, Tom22 and Tom7. Our proteomics finds no evidence of Tom7 or Tom22 in mitosomes, and sensitive hidden Markov model searches likewise fail to find any proteins encoded in the *Giardia* genome with similarity to Tom7 or Tom22 [25], [57]. Whether reflecting a secondary gene loss or the ancestral condition, GiTom40 would appear to be a selectively simple protein translocase. In addition to Tom40, mitochondria contain one other member of the mitochondrial porin family, the voltage-dependent anion channels (VDAC), which serve to exchange metabolites [61]. The absence of VDAC in *Giardia* mitosomes might reflect the disappearance of many of the metabolic pathways, and the concomitant decrease in metabolite flux across the outer membrane. It is likely that the *Giardia* Tom40, in addition to importing proteins, exchanges ions and small metabolites across the outer mitosomal membrane as has been demonstrated for the yeast Tom40 in mutants lacking VDAC [44], [62]–[64].

Another surprising result, one that can only be explained by a secondary gene loss, is the absence of the outer membrane protein Sam50 in *Giardia*. Sam50 is a component of the SAM (sorting and assembly machinery) complex, which is required for the assembly of both Tom40 and VDAC [48], [65]. The apparent absence of Sam50 from the *Giardia* genome and from our proteomics data is unique among eukaryotes. A putative Sam50 homologue has been predicted in the genomes of all eukaryotes, including trypanosomatids [58], [65] and mitosome- and hydrogenosome-containing protists (*C. parvum*, *E. cuniculi*, *E. histolytica* and *T. vaginalis*) [66]. Numerous phylogenetic and functional analyses indicate that Sam50 was derived from the Omp85/BamA protein present in the outer membrane of the ancestral, alpha-proteobacterial endosymbiont and it must, therefore, have been present in the earliest mitochondria [44]. It is not clear how *Giardia* Tom40 is assembled within the outer membrane without the assistance of the SAM complex. It is known that even in yeast Tom40 mediates the import of new molecules of Tom40 into mitochondria [67] and it is tempting to speculate that the *Giardia* Tom40 is capable of mediating its own import and membrane insertion, given the highly simplified nature of the TOM complex in mitosomes.

Our proteomics data support the hypothesis that ISC assembly is an important and possibly the only biosynthetic function of *Giardia* mitosomes. Previous phylogenic analyses have indicated that the ISC assembly machinery was obtained from the alpha-proteobacterial endosymbiont; nearly complete ISC assembly machinery is present from trypanosomatids to higher eukaryotes. Therefore, the absence of certain components, such as IscA-1, Iba57, and Ind, in the mitosomal machines (Table 8) is apparently due to a secondary loss of specific target proteins. Noteworthy, we did not identify any proteins that would carry FeS clusters in *Giardia* mitosomes, except for components of the FeS cluster assembly machinery itself. It seems likely then that the main role of mitosomes could be to export preassembled FeS clusters, or other compounds that are essential for the biogenesis of FeS proteins, to other cellular compartments. In mitochondria, the export of these enigmatic compounds is dependent on the membrane ABC “half-transporter” Atm1 [68] and sulfhydryl oxidase Erv1 [69]. In the mitosome-enriched fraction, we identified four ABC half-transporters by mass spectrometry, and another candidate was predicted based on phyletic profiling of the *G. intestinalis* genome (Table 2). However, compared to other Atm1 homologues, these candidates lack the x-loop with the conserved arginine, which is essential for known Atm1 transporters (Fig. S9). No protein with homology to Erv1 was found by proteomics or by analysis of the *Giardia* genome.

Another remaining question pertains to the source of ATP that is required for the multiple processes identified in mitosomes including FeS cluster assembly and export, organelle division, protein import and protein folding. In *E. histolytica*, it has been shown that a mitochondrial carrier family (MCF) protein localizes to mitosomes and exchanges ATP and ADP across the inner membrane, effecting the import ATP into mitosomes [20]. *E. cuniculi* mitosomes contain a distinct bacterial nucleotide transporter that may fulfill the same function [23], [24]. However, our proteomic analysis did not revealed a candidate nucleotide transporter in the mitosomes of *Giardia* leaving open the question of ATP acquisition.

In conclusion, using iTRAQ-based mass spectrometry and bioinformatics we identified 139 candidate mitosomal proteins. Mitosomal localization was confirmed experimentally for 20 of 44 proteins tested, suggesting the complete mitosomal proteome of *Giardia* to be of the order of 50-100 proteins. Previous genome analyses failed to predict any of the novel mitosomal proteins identified here [70]; only by combining quantitative mass spectrometry and bioinformatics were these novel proteins identified. The small proteome of the *G. intestinalis* mitosome indicates a marked reduction in mitochondrial metabolic activity and reduced requirements for organelle biogenesis. These do not mirror the reductions seen in the mitosomal proteome of *Cryptosporidium*, supporting the view that lineage-specific reductions produce organelles with distinct metabolic pathways and specific “short-cut” pathways for biogenesis. Our findings provide new insight into aspects of mitochondrial evolution and the basis from which to begin reconstructing the details of precisely how these organelles are built and replicated to support *Giardia* growth and division.

## Methods

### Cell culture and fractionation

*G. intestinalis* strain WB (American Type Culture Collection) was grown in TYI-S-33 medium supplemented with 10% heat-inactivated bovine serum and 0.1% bovine bile [9]. Trophozoites were freeze-detached, washed in PBS and collected by centrifugation. Cells were then resuspended in hypotonic buffer (12 mM MOPS, pH 7.4) and incubated for 15 minutes. The cells were then pelleted at 680× g for 15 minutes, resuspended in the same buffer with DNase I (40 µg/mL) and homogenized by 10 passages through a 25G needle. After homogenization, the isotonicity was immediately restored with the addition of an equal volume of 500 mM sucrose in MOPS buffer. The homogenate was then treated with trypsin (200 µg/mL) for 10 minutes at 37°C to release the organelles from the cytoskeleton. Proteases inhibitors were then added (5 mg/mL of soybean trypsin inhibitor, leupeptin and TLCK), and the homogenate was diluted and centrifuged for 20 minutes at 2760× g to remove cellular debris. The collected supernatant was centrifuged using a Beckman rotor Ti 50 at 20,000 rpm for 30 minutes. After centrifugation, the pellet was collected and washed in SM buffer (250 mM sucrose and 12 mM MOPS, pH 7.4). Next, the pellet was resuspended in 0.5 mL of SM buffer and layered onto a discontinuous density OptiPrep (Axis-Shield PoC AS, Oslo, Norway) gradient, which consisted of 1 ml each of 15%, 20%, 25%, 30% and 50% OptiPrep diluted in 12 mM MOPS buffer. The gradient was centrifuged for 24 h in a Beckman SW 40 rotor at 120,000× g at 4°C. Fractions (1 mL each) were collected, washed and analyzed by immunoblot using a polyclonal rabbit anti-IscU antibody [71], [72].

### Mass spectrometry analysis

Samples of two selected fractions (100 µg of total protein each) were precipitated with acetone at −20°C for 2 hours and then pelleted at 13,000× g for 15 min. The proteins were trypsin digested and labeled with sample-specific iTRAQ reagents according to the manufacturer's protocol (Applied Biosystems). Labeled samples were mixed and precipitated with acetone. The pellet was dissolved in 2 M urea in HPLC grade water, and the solution was subjected to IEF using 7 cm immobilized pH 3–10 gradient strips (Bio-Rad) for 20,000 VHrs. The strips were cut into 2-mm wide slices, and peptides were extracted using 50% ACN with 1% TFA. Extracted peptides were then separated using an Ultimate 3000 HPLC system (Dionex) coupled to a Probot micro-fraction collector (Dionex). The samples were loaded onto a PepMap 100 C18 RP column (3 µm particle size, 15 cm long, 75 µm internal diameter; Dionex) and separated with a gradient of 5% (v/v) ACN and 0.1% (v/v) TFA to 80% (v/v) ACN and 0.1% (v/v) TFA over 60 min at a flow rate of 300 nl/min. The eluate was mixed 1∶3 with matrix solution (20 mg/mL α-cyano-4-hydroxycinnamic acid in 80% ACN) prior to spotting onto a MALDI target. Spectra were acquired using a 4800 Plus MALDI TOF/TOF analyzer (Applied Biosystems/MDS Sciex) equipped with a Nd:YAG laser (355 nm, 200 Hz firing rate). All spots were measured in MS mode; up to 10 of the strongest precursors were selected for MS/MS analysis, which was performed using collision energy of 1 kV and operating pressure of the collision cell of 10^−6^ Torr. Peak lists from the MS/MS spectra were generated using GPS Explorer v. 3.6 (Applied Biosystems/MDS Sciex) subtraction of baseline enabled with peak width 50, smoothing with Savitsky-Golay algorithm of polynomial order of four and three points across peak, minimum signal to noise (S/N) 3, local noise window 250 m/z, cluster area S/N optimization enabled with S/N threshold 5. Spectra were searched with locally installed Mascot v. 2.1 (Matrix Science) against the *Giardia*DB release 1.3 annotated protein database (4892 sequences, 2663813 residues) and *GiardiaDB* release 1.2 Open Reading Frame translations greater than 50 amino acids (85612 sequences, 9633221 residues). The database search criteria were as follows: trypsin; one missed cleavage site allowed; fixed modifications iTRAQ 4-plex on *N*-terminal- and lysine *ε*-amino group, methylthiolation of cysteine; variable modification methionine oxidation; peptide mass tolerance of 100 ppm; MS/MS tolerance of 0.2 Da; maximum peptide rank of 1, minimum ion score C.I. (peptide) of 95%.

### Bioinformatics

Bioinformatics searches based on simple pair-wise alignment Psi-BLAST and hidden Markov models (HMMs) were applied to verify the automatic protein annotations and estimate their functions. Protein sequences (<1000 residues) were submitted (*i*) against a 90% redundancy reduced NCBI nr database for 8 iterations at an e-value cutoff of 10^−3^ and (*ii*) against Pfam 23.0 A+B database of families represented by multiple sequence alignments and hidden Markov models at an e-value of 0.044 (http://pfam.sanger.ac.uk/search). Where noted in the text, tailored HMM libraries were used to search for components of the protein import machinery [16].

Programs based on a combination of artificial neural networks (TargetP) and hidden Markov models (SignalP, both http://www.cbs.dtu.dk/services/) together with PsortII (http://psort.ims.u-tokyo.ac.jp/) were used to predict the subcellular localizations of the proteins. The secondary structures and topologies of alpha-helical integral membrane proteins were predicted using two bioinformatics tools: TMHMM, a program based on hidden Markov models (http://www.cbs.dtu.dk/services/), and Memsat3 (http://bioinf.cs.ucl.ac.uk/memsat/).

### Transformation of *G. intestinalis* and immunofluorescence

Selected genes were amplified by PCR from genomic *Giardia* DNA and inserted into the pONDRA plasmid [73]. Table S6 contains a list of primers that were used for subcloning of genes into expression vector. Cells were transformed and selected as described previously [16]. *G. intestinalis* cells expressing the recombinant proteins fused to a hemagglutinin tag (HA) at the C-terminus were fixed and stained for immunofluorescence microscopy with a mouse monoclonal anti-HA antibody. A secondary AlexaFluor-488 (green) donkey anti-mouse antibody was used.

### Preparation of recombinant proteins and enzyme assay

The coding region of GiOR-1 and [2Fe2S]ferredoxin was subcloned into pET42b and pET3a (Invitrogen), respectively and expressed in *Escherichia coli* BL21. The bacteria were induced with 0,5 mM IPTG (isopropyl-β-D-thiogalactopyranoside) and grown at 37°C in LB medium. For expression of GiOR-1, the LB medium was supplemented with 250 µM flavin adenine dinucleotide (FAD) and 250 µM flavin mononucleotide (FMN), whereas the LB medium supplemented with 400 µM ferric ammonium citrate was used for expression of ferredoxin. After induction, the cells were incubated overnight at 4°C. The harvested cells were homogenized, and soluble fraction was obtained by centrifugation at 250,000× g, 1 h, 4°C. The his-tagged GiOR-1was affinity purified under native conditions using a Ni-NTA column (Qiagen) according to manufacture's protocol. Ferredoxin was isolated by gel filtration chromatography using a BioLogic HR system (BioRad).

Enzyme activity of GiOR-1 was assayed spectrophotometrically at 25°C in anaerobic cuvettes under nitrogen atmosphere. The activity was monitored as a rate of NADPH or NADH (0,25 mM) oxidation in the presence of dichlorophenol*-*indolephenol (0,1 mM) or ferredoxin at 340 nm, or as a rate of methyl viologen (2 mM) reduction at 600 nm. NADPH oxidase activity was measured under aerobic conditions at 340 nm. The enzymatic activity was determined in phosphate buffer (100 mM KH_2_PO_4_/KOH, 150 mM NaCl, pH 7,4). Protein concentration was determined according to Lowry method.

## Supporting Information

Figure S1 Sequence alignment of Giardia Nfu against ekaryotic and prokaryotic orthologues. Conserved thioredoxin-like CXXC motif is shown in green. Giardia, *Giardia intestinalis* EAA38809; Trichomonas, *Trichomonas vaginalis*, TVAG_146780; Trypanosoma, *Trypanosoma brucei*, XP_845796; Leishmania, *Leishmania infantum*, XP_001470367; Toxoplasma, *Toxoplasma gondii*, XP_002371042; Plasmodium, *Plasmodium falciparum*, CAX64255; Saccharomyces, *Saccharomyces cerevisiae*, NP_012884; Homo, *Homo sapiens*, AAI13695; Rickettsia, *Rickettsia prowazekii*, NP_221029; Stigmatella, *Stigmatella aurantiaca*, ZP_01463912.
(PDF)

Figure S2 Sequence alignment of *Giardia* IscA against eukaryotic and bacterial orthologs. The conserved cysteine residues are highlited in yellow. Organism names and accession numbers: Giardia, *Giardia intestinalis* GL50803_14821; Trichomonas, *Trichomonas vaginalis* TVAG_055320; Trypanosoma, *Trypanosoma cruzi* XP_806610; Saccharomyces, *Saccharomyces cerevisiae* Q12425; Homo, *Homo sapiens* NP_919255; Arabidopsis, *Arabidopsis thaliana* NP_179262; Chlamydomonas, *Chlamydomonas reinhardtii* XP_001697636; Rickettsia, *Rickettsia conorii* NP_360365; Escherichia, Escherichia coli CAQ32901; Mycobacterium, *Mycobacterium leprae* NP_301657.
(PDF)

Figure S3 Sequence alignment of *Giardia* Jac1 against eukaryotic and bacterial orthologs. The conserved HSP70 interactin site is highlited in green. Organism names and accession numbers: Giardia, *Giardia intestinalis*, GL50803_17030; Trichomonas, *Trichomonas vaginalis*, TVAG_422630; Trypanosoma, *Trypanosoma brucei*, XP_843770; Leishmania, *Leishmania infantum*, XP_001466207; Plasmodium, *Plasmodium falciparum*, CAX64223; Toxoplasma, *Toxoplasma gondii*, XP_002368309; Naegleria, *Naegleria gruberi*, EFC47366; Saccharomyces, *Saccharomyces cerevisiae*, NP_011497; Homo, *Homo sapiens*, AAN85282; Escherichia, *Escherichia* coli, YP_002408666.
(PDF)

Figure S4 Sequence alignment of *Giardia* Mge1 against eukaryotic (Mge1) and bacterial (GrpE) orthologs. The residues in yellow indicate a GrpE dimer interface. HSP70 binding sites are shown in green (Harrison CJ, Hayer-Hartl M, Di Liberto M, Hartl F, Kuriyan J, Crystal structure of the nucleotide exchange factor GrpE bound to the ATPase domain of the molecular chaperone DnaK, Science 1999, 276:431–435. *Giardia intestinalis*, GL50803_1376; *Homo sapiens*, NP_079472; *Saccharomyces cerevisae*, NP_014875; *Escherichia coli*, NP_417104; *Arabidopsis thaliana*, NP_567757; *Trichomonas vaginalis*, XP_001329309; *Trypanosoma brucei*, XP_845338; *Dictyostelium discoideum*, XP_638912; *Bacillus subtilis*, NP_390426; *Halobacterium sp*., NP_279548.
(PDF)

Figure S5 Sequence alignment of *G. intestinalis* mitosomal oxidoreductase OR-1 (GL50803_91252), against *G. intestinalis* non-mitosomal paralogue OR-2 (GL50803_15897) and structurally related proteins containing flavodoxin-like FMN-binding domain (conserved residua in blue), FAD binding pocket (residua involved in FAD binding in green) and NADP(H) binding site (residua involved in NADP(H) in red). *Saccharomyces cerevisiae* Tah18, DAA11472; *Homo sapiens* NDOR, NADPH dependent diflavin oxidoreductase, AAH15735; *Rattus norvegicus* NOS, nitric oxide synthase, AAC13747; *Rattus norvegicus* CPR, cytochrome P450 reductase, NP_113764; *Escherichia coli* SiR, sulfite reductase, YP_002330508; *Homo sapiens* MSR, methionine synthase reductase, NP_076915; *Trichomonas vaginalis* Hyd, hydrogenase, TVAG_136330; *Leptospira interrogans* FNR, ferredoxin reductase, YP_003372.
(PDF)

Figure S6 Conserved glycine which is present in all GroES and Cpn10 homologues is shown in green. Hsp60 binding site is shown in yellow (van der Giezen M, León-Avila G, Tovar J. (2005) Characterization of chaperonin 10 (Cpn10) from the intestinal human pathogen *Entamoeba histolytica*. Microbiology 151:3107-15). *Giardia intestinalis* GL50803_29500; *Trichomonas vaginalis* TVAG_191660; *Saccharomyces cerevisiae* NP_014663.1; *Homo sapiens* XP_001118014.1; *Leishmania infantum* XP_001470405.1; *Plasmodium falciparum* PFL0740c; Arabidopsis thaliana NP_563961.1; *Dictyostelium discoideum* XP_636819.1; *Mycobacterium tuberculosis* NP_217935.1; *Escherichia coli* NP_290775.1.
(PDF)

Figure S7 Sequence alignment of *Giardia* Pam16 against eukaryotic Pam 16 orthologues and giardial Pam 18 paralogue. Conserved leucin in an interacting hydrofobic pocket is shown in green (D'Silva PR, Schilke B, Hayashi M, Craig EA (2008) Interaction of the J-protein heterodimer Pam18/Pam16 of the mitochondrial import motor with the translocon of the inner membrane. Mol Biol Cell 19:424-32). The typical HPD motif (in blue) present in Pam18 is degenerated in Pam16, in yellow (Mokranjac D, Bourenkov G, Hell K, Neupert W, Groll M (2006) Structure and function of Tim14 and Tim16, the J and J-like components of the mitochondrial protein import motor. EMBO J 25:4675-85). *Giardia intestinalis* Pam 16 GL50803_19230; *Trichomonas vaginalis* TVAG_470110; *Toxoplasma gondii* XP_002367323.1; *Saccharomyces cerevisiae* NP_012431.1; *Neurospora crassa* XP_960477.1; *Pediculus humanus* XP_002428010.1; *Schistosoma japonicum* CAX74438.1; *Homo sapiens* NP_057153.8; *Mus musculus* NP_079847.1; *Xenopus laevis* NP_001084733.1; *Giardia intestinalis* Pam 18 XP_002364144.
(PDF)

Figure S8 Protein sequence alignment of VAP (VAMP-associated protein) homologues. Domain structure is depicted for each represented sequence according to HHPRED (http://toolkit.tuebingen.mpg.de/). Major sperm protein domain, yellow. Coiled-coil domain in green and dimerization motif GXXXG in red. The *G. intestinalis* VAP contains all protein characteristics as described for human homologue.
(PDF)

Figure S9 Sequence alignment of *Giardia* AbcB transporter against mitochondrial and bacterial orthologs. *Giardia intestinalis* AbcB, GL50803_17315; *Saccharomyces cerevisiae* Atm1, NP_014030; *Saccharomyces cerevisae* Mdl1, NP_013289; *Homo sapiens* AbcB7, NP_004290; *Homo sapiens* AbcB10, NP_036221; *Arabidopsis thaliana* Atm3, NP_200635; *Naegleria gruberi* Atm1,XP_002683195; *Rhodobacter sphaeroides* AbcB, YP_001168064; *Halobacterium sp.* AbcB, NP_279266. Walker A part of a conserved ATP-binding motif in yellow; Q-loop part of a conserved ATP-binding motif in green; ABC signature, a conserved sequence specific for ABC proteins in pink; Walker B part of a conserved ATP-binding motif in blue; D-loop part of a conserved ATP-binding motif in red; H-loop part of a conserved ATP-binding motif in purple; X-loop contains a conserved arginine in AbcB transporters (•), which is not present in Giardia sequence, in cyan (Dawson RJ, Locher KP (2006) Structure of a bacterial multidrug ABC transporter. Nature 443:180-185; Bernard DG, Cheng Y, Zhao Y, Balk J (2009) An allelic mutant series of ATM3 reveals its key role in the biogenesis of cytosolic iron-sulfur proteins in Arabidopsis. Plant Physiol 151: 590-602).
(PDF)

Table S1 Complete list of proteins identified by LC MS/MS in mitosomal fractions labelled by iTRAQ reagents.
(PDF)

Table S2 List of *Giardia* proteins within the mitosomal distribution range (MiD) identified by LC MS/MS.
(PDF)

Table S3 Identification of protein families using PfamA+B databases.
(PDF)

Table S4 Predictions of cellular localization.
(PDF)

Table S5 Orthology phylogenetic profililng. Genomes of *G. intestinalis* and *Rickettsia typhi* were compared using orthology phylogenetic profile tool at GiardiaDB.
(PDF)

Table S6 List of primers that were used for subcloning of genes into expression vector pONDRA to investigate subcellular localization of corresponding gene products in *G. intestinalis*.
(PDF)
