# Supplementary material for: The Minimal Proteome in the Reduced Mitochondrion of the Parasitic Protist Giardia intestinalis
Source: PLoS One. 2011 Feb 24;6(2):e17285. doi: 10.1371/journal.pone.0017285 (PMC3044749; doi:10.1371/journal.pone.0017285)
Supplement: Figure S1 — Sequence alignment of Giardia Nfu against ekaryotic and prokaryotic orthologues. Conserved thioredoxin-like CXXC motif is shown in green. Giardia, Giardia intestinalis EAA38809; Trichomonas, Trichomonas vaginalis, TVAG_146780; Trypanosoma, Trypanosoma brucei, XP_845796; Leishmania, Leishmania infantum, XP_001470367; Toxoplasma, Toxoplasma gondii, XP_002371042; Plasmodium, Plasmodium falciparum, CAX64255; Saccharomyces, Saccharomyces cerevisiae, NP_012884; Homo, Homo sapiens, AAI13695; Rickettsia, Rickettsia prowazekii, NP_221029; Stigmatella, Stigmatella aurantiaca, ZP_01463912. (PDF) [file pone.0017285.s001.pdf]

Fig S1

```
Giardia         -----------~---------------------------------------------------------M    1
Trichomonas     ----------------------------------------------------------------------    1
Trypanosoma     ------MIKFTLRYFAVSSSTFYRLPRRMSTPTTRWAAFCALYSHRELLLCTICRGVCSGKSSQRSIVVE   64
Leishmania      -MEKILRRSGVRMTSYAAWSITGASLGTRVPCSSTVRRSLTSSLVILAGGAFSGSSFAARAVQCRCIVVE   69
Toxoplasma      ----------------------------------------------------------------------    1
Plasmodium      ----------------------------------------------------------------------    1
Saccharomyces   ---------------------------------------------MFKSVAKLGKSPIFYLNSQRLIHIK   25
Homo            --------MAATARRGWGAAAVAAGLRRRFCHMLKNPYTIKKQPLHQFVQRPLFPLPAAFYHPVRYMFIQ   62
Rickettsia      ------------------------------------------------------------------MFIQ    4
Stigmatella     ----------------------------------------------------------------MSVNIQ    6

Giardia         LSLLPTHSMLGLVTRNLLITSSQSSKPRSYIMAFDGLVPEAPSLQNDLMKVKGVKKLSISPTGVTLTV-R   70
Trichomonas     ----------------------------------------------------------------------    1
Trypanosoma     KNETPNPDCLRFYSMELSFLPPG--RSLDLPDAQHAG---KSPLAELLFSISGVQSVFLADEYITVGK-V  128
Leishmania      TNETPNPDCMRFFSMDVSFLKPE--FSMDIPSPAQAY---KSPLAEALFGVAGVQAVFLADEYVTVRK-H  133
Toxoplasma      ---------------------MPEKGTGTFRCTQAHT----LSLLFCTFQIDGTSSVLIAGGYVTVVK-A   44
Plasmodium      MIHFKNAFSYRHSLCMMRTFHFNIYKNVKFSNYITKALN--KYIVTKQSTNQRFFSIINNSDYINYIN--   66
Saccharomyces   TLTTPNENALKFLSTDGEMLQTRGSKSIVIKNTDENLINHSKLAQQIFLQCPGVESLMIGDDFLTINK-D   94
Homo            TQDTPNPNSLKFIPG-KPVLET---RTMDFPTPAAAFR---SPLARQLFRIEGVKSVFFGPDFITVTKEN  125
Rickettsia      TEETPNPDAIKFFPG-QEISVD---QPVFFSELAEVKG--RSALAESLFHINNVKSVFLGSDFITVTK-Q   67
Stigmatella     LEWTPNPSTLKYVVD-RRLLSS---GAVNFTRREEAEQ--KSPLARKLMDIQGVTAVMLGLNFVTVTK-G   69

Giardia         SQGIWPSVRSAIEDKLYAAFDPST---PHTPDELR---------LAIQEMLSTGPRAPNNIRRASELLIR  128
Trichomonas     ----------------------------MLAAVKNSTFGFGNFFIRSFAKEAVKETDADKLFQRVNKIIE   42
Trypanosoma     PHVDWGSLVPQIQECIVEFAESGVGVLSEEGEA----------CFVDNNNDTDPEDDDDEVVLAVKELLS  188
Leishmania      PQADWAALIPIIKEVIVEFAESKENVLSAAGEE----------ELLGYNNDTEPDDDDDEVVLAVKELLA  193
Toxoplasma      RDTDWKDLEEPVKRCIQDHLTSGIPAVQRAVSSEDVSG-AAEGRPQVPEKKNAKSEEEEDLSEAIRELLH  113
Plasmodium      ---EINKFIDIFEKNVDNKSTNEDHIIPVLEKIK-------------NEKIYKDNEDIMEIISSIKLLIE  120
Saccharomyces   RMVHWNSIKPEIIDLLTKQLAYGEDVISKEFHAVQEE--EGEGGYKINMPKFELTEEDEEVSELIEELID  162
Homo            EELDWNLLKPDIYATIMDFFASG---LPLVTEE----------TPSG----EAGSEEDDEVVAMIKELLD  178
Rickettsia      ARGNWQVIKPEILMVIMDHFISG---FPVFNEN----------TKID--DEKHNLDMLSEIEKQIIETIE  122
Stigmatella     DEGEWDELNDAVMSTLDAHLGSDE---PVVDEA-----------AVAAARAAP-AEGGSSVEQRIREILD  124

Giardia         AAINPFLARDGGSCSYRRHEVTDKGLVVYIELHGNCSGCSKSTTTMNTFVIGEFKKYIPDIHT-VRCTNA  197
Trichomonas     EKVRPFIKQDHGDIELVDIK----NGCMIVQLEGACEGCGCKNTTLYNGVLGTVQEEIPEITN-IRQKMP  107
Trypanosoma     ARIRPLLRADGGNVRYISMD----DGTVFVLLEGACKSCPSSGVTLKNGIERMLMHWIPEVVE-VQECTD  253
Leishmania      TRIRPMLRADGGNVRFIDMD----EGTVFLLLEGSCKSCPSSHVTLKSGIERMLMHWIPEVVE-AQEVSD  258
Toxoplasma      MRARPMLQADGGDLEMMRFDE--ETGIVWVHLKGSCEGCPSSLITVKRGMKQMLQYYIPEVRTILRVDRS  181
Plasmodium      KRVRPIILNDGGDIKFICFDVD--KGIVYVQLEGACVTCAQSEVTLQYMIKNMLTYYISEIKEIKNVSKD  188
Saccharomyces   TRIRPAILEDGGDIDYRGWDP--KTGTVYLRLQGACTSCSSSEVTLKYGIESMLKHYVDEVKE-VIQIMD  229
Homo            TRIRPTVQEDGGDVIYKGFE----DGIVQLKLQGSCT-CPSSIITLKNGIQNMLQFYIPEVEG-VEQVMD  242
Rickettsia      TRVRPFVTQDGGDIIYKGFE----SGVVKLALRGACLGCPSSTITLKNGIESMLKHFIPEVQE-VKAVEE  187
Stigmatella     AEIRPAVAQDGGDITLDRYE----NGVVYLHMQGSCSGCPSSTATLKMGIEGRLREAIPEVTE-VVSI--  187

Giardia         ------------------------------------------------------  197
Trichomonas     FDDFE-------------------------------------------------  112
Trypanosoma     EMASDLLAEKELRRKLK-KDEVSASQSN--------------------------  280
Leishmania      EVAVDILSEKRLRKQLKEKGEVVKAK----------------------------  284
Toxoplasma      ELEGVVAITVASPDEYHSTQWVTLPSANALCHLTQVEDVRQCDENGDPLEDDEE  235
Plasmodium      GIIL--------------------------------------------------  192
Saccharomyces   PEQEIALKEFDKLEKKLESSKNTSHEK---------------------------  256
Homo            DESDEKEANSP-------------------------------------------  253
Rickettsia      DFK---------------------------------------------------  190
Stigmatella     ------------------------------------------------------  187
```
