# Supplementary material for: The Minimal Proteome in the Reduced Mitochondrion of the Parasitic Protist Giardia intestinalis
Source: PLoS One. 2011 Feb 24;6(2):e17285. doi: 10.1371/journal.pone.0017285 (PMC3044749; doi:10.1371/journal.pone.0017285)
Supplement: Figure S2 — Sequence alignment of Giardia IscA against eukaryotic and bacterial orthologs. The conserved cysteine residues are highlited in yellow. Organism names and accession numbers: Giardia, Giardia intestinalis GL50803_14821; Trichomonas, Trichomonas vaginalis TVAG_055320; Trypanosoma, Trypanosoma cruzi XP_806610; Saccharomyces, Saccharomyces cerevisiae Q12425; Homo, Homo sapiens NP_919255; Arabidopsis, Arabidopsis thaliana NP_179262; Chlamydomonas, Chlamydomonas reinhardtii XP_001697636; Rickettsia, Rickettsia conorii NP_360365; Escherichia, Escherichia coli CAQ32901; Mycobacterium, Mycobacterium leprae NP_301657. (PDF) [file pone.0017285.s002.pdf]

Fig. S2

```
Giardia                                                   MLPALITPLVRSLTKNGIKITDAAVSSLRNILK----PSEFLRVNVMTSGCAGLTYK  53
Trichomonas                                                 MLSQFFSNFAQKKAAFSMTPAAIKQIKELLKTD-FENKMLRITLKSGGCAGFQYD  54
Trypanosoma                                         MLRRSYLLFCVPHLSAVYGRSFTVSSRVWHRINEKNGEEGFQNDQRFLRLAVDSGGCHGYKYH  63
Saccharomyces    MQAKLLFTRLNFRRPSTTLRQFPLTCFLFHSKAFYSDLVTKEPLITPKRIINKTPGLNLSISERASNRLAEIYRNS---KENLRISVESGGCHGFQYN  95
Homo                      MAAAWGSSLTAATQRAVTPWPRGRLLTASLGPQARREASSSSPEAGEGQIRLTDSCVQRLLEITE----GSEFLRLQVEGGGCSGFQYK  85
Arabidopsis                                          MKASQILAAAAARVGPALRKQVLTLTDEAASRVHHLLQQRQKP-F-LRLGVKARGCNGLSYT  60
Chlamydomonas                                     MKSTLGLLAEALKETARSPRMRKAAVELTEAAAGRIKELLNKRHKE-Y-LKLGVKTRGCSGMSYT  63
Rickettsia                                                             MKNVISLTDSAAKQIKLLIEKRAKPTFGIRVGVKSGGCAGQTYY  44
Escherichia                                                              MSITLSDSAAARVNTFLANRGKG-FGLRLGVRTSGCSGMAYV  41
Mycobacterium                                                   MAVQNELSAKTHGVILTDVAATKAKSLLDQEGRDDLALRIAVQPGGCAGLRYN  53

Giardia       FAVDTD---RRKDDD----VTKKGGVE---------------LRVDNKALAYVKGSTIDFISEPFRQYFVLKD-NPQSSGSCSCGESFEIPGLDIVPTPCH 131
Trichomonas   FSFDSA---ARKGDH----LFQQDGAA---------------VVLDDKALLYLRGAELDYSSDIFSSYFKVNIPLESELHSCHCGKSVGTDETVGKHKCSH 133
Trypanosoma   FSFEENSALVPEEDV----VVAETDVLPVGAEGRSGVEPPPRVVVDRHSLTKLQAAVIDFHSELKGAAFVVVG-NELVDESCACAMSFSIKKRQPQK     155
Saccharomyces LTLEPATKPDIKNDVKDKEFSDDLDDDDSKDIIYVLPEDKGRVIIDSKSLNILNNTTLTYTNELIGSSFKIIN--GSLKSSCGCGSSFDIEN          185
Homo          FSLDTV---INPDDR----VFEQGGAR---------------VVVDSDSLAFVKGAQVDFSQELIRSSFQVLN-NPQAQQGCSCGSSFSIKL          154
Arabidopsis   LNYADE---KGKFDE----LVEEKGVR---------------ILVEPKALMHVIGTKMDFVDDKLRSEFVFI--NPNSQGQCGCGESFMTTSTSSAKQSAS 137
Chlamydomonas LNYADN---KGKFDE----VVEDKGVR---------------IIIEPQALMHVLGTKMDYVKDDLRQEFVFV--NPNAKGTCGCGESFTT            129
Rickettsia    VEYADS---KNQFDE----VVEEKGVR---------------ILIDPKALMYILGSEMDYVETKFKSQFTFT--NPNEKASCGCGKSFRV            110
Escherichia   LEFVDE---PTPEDI----VFEDKGVK---------------VVVDGKSLQFLDGTQLDFVKEGLNEGFKFT--NPNVKDECGCGESFHV            107
Mycobacterium LFFDDR---TLDGDL----TAEFGGVT---------------LTVDRMSAPYVEGASIDFVDTIEKQGFTID--NPNANGSCACGDSFN             118
```
