# Supplementary material for: The Minimal Proteome in the Reduced Mitochondrion of the Parasitic Protist Giardia intestinalis
Source: PLoS One. 2011 Feb 24;6(2):e17285. doi: 10.1371/journal.pone.0017285 (PMC3044749; doi:10.1371/journal.pone.0017285)
Supplement: Figure S3 — Sequence alignment of Giardia Jac1 against eukaryotic and bacterial orthologs. The conserved HSP70 interactin site is highlited in green. Organism names and accession numbers: Giardia, Giardia intestinalis, GL50803_17030; Trichomonas, Trichomonas vaginalis, TVAG_422630; Trypanosoma, Trypanosoma brucei, XP_843770; Leishmania, Leishmania infantum, XP_001466207; Plasmodium, Plasmodium falciparum, CAX64223; Toxoplasma, Toxoplasma gondii, XP_002368309; Naegleria, Naegleria gruberi, EFC47366; Saccharomyces, Saccharomyces cerevisiae, NP_011497; Homo, Homo sapiens, AAN85282; Escherichia, Escherichia coli, YP_002408666. (PDF) [file pone.0017285.s003.pdf]

Fig. S3

```
Giardia         ----------------------------------MALPTLFSANSSTILGRIERYLKHACWKCG------ 30
Trichomonas     -----------------------------------MLSPSANYIKKAFAAAVQHTCSSACWNCN------ 29
Trypanosoma     ----------------------------------------------MVVVGMNRRFLATSAWLG------ 18
Leishmania      ----------------------------MLAQCSLFARPRVFKGLVLRSHAPVACWDASLPWMA------ 36
Plasmodium      ------MKKIKYLSILWKHKKINICNHKNCYTYNLAQNFFHSNKKNNIDSNNYNGNILSNKNKVKHFKCPK 64
Toxoplasma      ----------------MQSLGFGVVGQREFSEATGGKTSREATALSPASDNLTEKLSQAGLVHSTSCTN- 53
Naegleria       MHRLPRIILNRTTSTLSTLNKSGVCLFQNLSPSSSNNSLLRRMSTCSKIPDKECPRSTQPLSSNSLHHPC 70
Saccharomyces   ---------------------------------------------------------------------- 1
Homo            -------------------------MWRGRAGALLRVWGFWPTGVPRRRPLSCDAASQAGSNYPRCWSCG 45
Escherichia     ---------------------------------------------------------------------- 1

Giardia         -----TKQPVTILCSGCGALQPISRPTN--------------YFKLMGISETYDIDLKKLKQRYNMLQAR 81
Trichomonas     --HRLNTARNSLFCESCHKIQAPTCEN---------------FFQLFNQPKQYQFDLKKLDKTYLSLQRK 82
Trypanosoma     --FSNGMTRNLHTAG------TVGRGN---------------FFAYLSLPKSPDLDAADVQRSYHKLQRR 65
Leishmania      --QQQRRCSSFASATPAAPSTDAGKGN---------------YFHFFRIAKHPDLDVAALQKKYHNLQRL 89
Plasmodium      CNNNISTDTVSFNCEVCKALFNIDIFRN------------FNIFELFNIEVNYDIDKSHLKKKFNEIQNI 122
Toxoplasma      -CKAAPARKYQIFCSNCGDALVPQYGDANKR--------DFSYFEFLQMNPTFDIDRAALEAKYKQLEKR 114
Naegleria       WNCSEELSCNDLFCAHCNKIQPPSCNNNSKVISTSGEDISIDYFTLMDMPKEFSINPKVLHEKFKELQRV 140
Saccharomyces   ------------------------MLKYLVQRRFTSTFYELFPKTFPKKLPIWTIDQSRLRKEYRQLQAQ 46
Homo            GPWGPGREDRFFCPQCRALQAPDPTRD---------------YFSLMDCNRSFRVDTAKLQHRYQQLQRL 100
Escherichia     ----------------------------------------MDYFTLFGLPARYQLDTQALSLRFQDLQRQ 30

Giardia         IHPDVIGKEVRTPNAPPG------------------LLQLAESLSSEVNHAHDVLKDDMRRAEYLASLYN 133
Trichomonas     VHPDRFYSKSEKEKE------------------------LSIKASGCINEGYHTLRNPVSRGEYLLHLFN 128
Trypanosoma     VHPDLANVQDANNSTEPPGGVVGS------VTTSTTTDVANVDDSMYANLSYETLRDPFRRCKYLSRLSR 129
Leishmania      VHPDQRHVQAQQQQAEAADVAPATSGSPLPFACTSNETKSSTDVSTYANAAYETLRAPYSRCRYLSRLVK 159
Plasmodium      YHPDKNAQNVEVDEIN--------------------------EVSSYLNNAYKTLSNDVERALYLLKMEY 166
Toxoplasma      LHPDKHVHADQEYHDR------------------------LAKHRTKVIEAVSALKNPAKRALHLLAHHS 160
Naegleria       LHPDKFAMKSEVEKK------------------------ISIMQSAIVNTAYQTLKNTKSRAEYLLKVKE 186
Saccharomyces   HHPDMAQQGSE--------------------------------QSSTLNQAYHTLKDPLRRSQYMLKLLR 84
Homo            VHPDFFSQRSQTEKD------------------------FSEKHSTLVNDAYKTLLAPLSRGLYLLKLHG 146
Escherichia     YHPDKFASGSQAEQL------------------------AAVQQSATINQAWQTLRHPLMRAEYLLSLHG 76

Giardia         VSASAASP-----------------------------------ALLIDQMVLRDQIASFHHKKNAKKLSE 168
Trichomonas     EKVLSDVP----------------------------------QDFLMEVLEFHEEMDSAT---EAEELIK 161
Trypanosoma     AEAVKGRPLDPLEEEMLMSDDDGRNVEDNKRLQDAGENMSLPEEFLAEMMAANEYVFSPE---ETN-EYR 195
Leishmania      AEEVKGSPLSAAEEEELLVEDDQR----TMKAREVRPDAPMSDDFLMEMLAMNELIFAGD---SSDEGVR 222
Plasmodium      NYMISEDECMDD------------------------------DEFLSEIIKINVEISKPD-----ANIEL 201
Toxoplasma      PHSSEHVEDEPDADR------------------------VTDGDLLQEVFELNEALEMVT---SREELDE 203
Naegleria       IDLLGPCNYKSVN------------------------DVQLPPEFLMEIMEVNEYINEIDS--DRDQLTD 230
Saccharomyces   NIDLTQEQTSNEVTTS-------------------------DPQLLLKVLDIHDELSQMD---DEAGVKL 126
Homo            IEIPERTDYEMD------------------------------RQFLIEIMEINEKLAEAE---SEAAMKE 183
Escherichia     FDLASEQHTVRD------------------------------TAFLMEQLELREELDEIE---QAKDEAR 113

Giardia         MRHALTKRFSDCSELFGKALAEKD-------SNQMASLLCEMRFLTSTLDELKGRMNTLDIEE------- 224
Trichomonas     LLQKVQKMMKDLTSQLATDLEIKDGKLLN--AAEAAKTLSKLKYLSRIRDTLKQKIPVQKL--------- 220
Trypanosoma     DRLVILIAHLEERHMECYEATKAQWDAED--FQGFRRSVLEWTYVRNILEKAKDCLGNINEAVPKGV--- 260
Leishmania      RQWSVLRFDLEDRAVGYFKDAVKSWNDGD--MGAFHHIVHEWTYVATALNNLKERMLE------------ 278
Plasmodium      LTKEYKQKYEDYSKEIKLHFKEKN-------FNNILNVLKKLKFINRVLERLQNI--------------- 249
Toxoplasma      FKRRVDALLEKDERDLSRRFREKVG------------LERALDNTIGVSSSDSGSW-------------- 247
Naegleria       YHMDFNEKKRLIVKSIDEAFKKDDLDNVRINIAELNYIERTLQLVDNKLHLLDLKEHRHFANEPCADCEH 300
Saccharomyces   LEKQNKERIQDIEAQLGQCYNDKD-------YAAAVKLTVELKYWYNLAKAFKDWAPGKQLEMNH----- 184
Homo            IESIVKAKQKEFTDNVSSAFEQDD-------FEEAKEILTKMRYFSNIEEKIKLKKIPL----------- 235
Escherichia     LESFIKRVKKMFDTRHQLMVEQLDNETWDA-AADTVRKLRFLDKLRSSAEQLEEKLLDFLISGS------ 176

Giardia         -- 224
Trichomonas     -- 220
Trypanosoma     -- 260
Leishmania      -- 278
Plasmodium      -- 249
Toxoplasma      -- 247
Naegleria       NK 302
Saccharomyces   -- 184
Homo            -- 235
Escherichia     -- 176
```
