# Supplementary material for: The Minimal Proteome in the Reduced Mitochondrion of the Parasitic Protist Giardia intestinalis
Source: PLoS One. 2011 Feb 24;6(2):e17285. doi: 10.1371/journal.pone.0017285 (PMC3044749; doi:10.1371/journal.pone.0017285)
Supplement: Figure S4 — Sequence alignment of Giardia Mge1 against eukaryotic (Mge1) and bacterial (GrpE) orthologs. The residues in yellow indicate a GrpE dimer interface. HSP70 binding sites are shown in green (Harrison CJ, Hayer-Hartl M, Di Liberto M, Hartl F, Kuriyan J, Crystal structure of the nucleotide exchange factor GrpE bound to the ATPase domain of the molecular chaperone DnaK, Science 1999, 276:431–435. Giardia intestinalis, GL50803_1376; Homo sapiens, NP_079472; Saccharomyces cerevisae, NP_014875; Escherichia coli, NP_417104; Arabidopsis thaliana, NP_567757; Trichomonas vaginalis, XP_001329309; Trypanosoma brucei, XP_845338; Dictyostelium discoideum, XP_638912; Bacillus subtilis, NP_390426; Halobacterium sp., NP_279548. (PDF) [file pone.0017285.s004.pdf]

Fig S4

```
Giardia        ----------------------------------------------------MALSALSPSSMAGTSVLMRLMRGLVKPSSSTIEQQLKL 38
Homo           --------MAAQCVRLARRSLPALALSLRPSPRLLCTATKQKNSGQNLEEDMGQ--SEQKADPPATEKTLLEEKVKLEEQLKETVEKYKR 80
Saccharomyces  MRAFSAATVRATTRKSFIPMAPRTPFVTPSFTKNVGSMRRMRFYSDEAKSEESKENNEDLTEEQSEIKKLESQLSAKTKEASELKDRLLR 90
Escherichia    -----------------------MSSKEQKTPEGQAPEEIIMDQHEEIEAVEPEASAEQVDPRDEKVANLEAQLAEAQTRE---RDGILR 64
Trichomonas    ----------------------------MLSSQQYLPSLFVRTFVEKAAESAKQATEKAVANDKKPEEKPKPTIQELEAQIKDIRNRNLF 62
Trypanosoma    ---------MRALTFRSSLCAGRTAVGAMCFGRLWASSTSATEGSEKQNVTEDSETVSVAPVSPEAYAKLEKELSDAKERIAELKKEVLY 81
Dictyostelium  ------------MNSLIRRGLVSIRTSTVIKPSSFGLMRNNRFYSTENNQEAAK--PEETENKPAPG-SLEETIEKLKEELEETKKQLLY 75
Bacillus       --------------------------------MSEEKQTVEQNETEEQEIIEEQAAADEQQEETNESELLQNQINELQGLLEEKENKLLR 58
Halobacterium  ---MSDHAEHAADAADTDAPEGDDAGGDDGEQAGDDGTSALSERVRALDADNADALADDVAALEARVETLTDELADAEDEVADLTERVQT 87

Giardia        LLAERQSAAEAQADAVAEARIEATQRCLRELLKILDAVDGLHAQAKSHPKSRGALTKHLHEIQESIAATHSLADQVLDTLEAQRIAPNRL 128
Homo           ALADTENLRQRSQKLVEEAKLYGIQAFCKDLLEVADVLEKATQCVPK--EEIKDDNPHLKNLYEGLVMTEVQIQKVFTKHGLLKLN-PVG 167
Saccharomyces  SVADFRNLQQVTKKDIQKAKDFALQKFAKDLLESVDNFGHALNAFKE--EDL-QKSKEISDLYTGVRMTRDVFENTLRKHGIEKLD-PLG 176
Escherichia    VKAEMENLRRRTELDIEKAHKFALEKFINELLPVIDSLDRALEVADK-------ANPDMSAMVEGIELTLKSMLDVVRKFGVEVIA-ETN 146
Trichomonas    LLAEVENARRRFARLEVEMETYAVSKLAKDLLPVADNMGRIINSGAK---------QNVKDVIEAVKLVDAEFHNIFKRFKIEKIV-SKG 142
Trypanosoma    RAADAENARRIGSEDVTKAKAYGITSFGKDMLDVVDTLERGLEAITKLPQAEVEGHKTLSSIHTGIKLSLKLLLNNLAKHGIEKLDVAVG 171
Dictyostelium  TAADRENVRRFAKEDNEKAKKFGIQSFTKELLEVVDQLEMATNLFPK--EKL-DENKELKDLHEGVKMTEQLFLKIMGNQGLQRFN-PIG 161
Bacillus       VQADFENYKRRSRLEMEASQKYRSQNIVTDLLPALDSFERALQVEAD--------NEQTKSLLQGMEMVHRQLVEALKKEGVEAIE-AVG 139
Halobacterium  KQADFKNYKERAKRKQEEIRERATEDLVERLLDVRDNLDRALDQEES--------ESDEDGIREGVELTRDEFDRVLETEGVTEIRPEPG 169

Giardia        DMFDSSLHNAVRVIEDSSLPS-NTVCETLQPGLLHKGTVIRPAQVVINDIY-- 178
Homo           AKFDPYEHEALFHTPVEGKEP-GTVALVSKVGYKLHGRTLRPALVGVVKEA-- 217
Saccharomyces  EPFDPNKHEATFELPQPDKEP-GTVFHVQQLGFTLNDRVIRPAKVGIVKGEEN 228
Escherichia    VPLDPNVHQAIAMVESDDVAP-GNVLGIMQKGYTLNGRTIRAAMVTVAKAKA- 197
Trichomonas    QKFDPQYHDAIQMIDTRGSAPSGTIIDCTTEGYKIDKRLLRAAKVIVAK---- 191
Trypanosoma    AKFDPNFHDALLKVPPTAEAPPGHISTVLKTGYKIQDRVLRASQVGVASDD-- 222
Dictyostelium  EKFDFNNHHAIFELNDPTKEN-NTVGHVVKQGYKLHDRLVRPAMVGVNKIKPQ 213
Bacillus       QEFDPNLHQAVMQAEDENYGS-NIVVEEMQKGYKLKDRVIRPSMVKVNQ---- 187
Halobacterium  DSVDAARHEVMMRVDSD--QPAGTIVDVYRPGYEMSGRVVRAAQVTVSEE--- 217
```
