# Supplementary material for: The Minimal Proteome in the Reduced Mitochondrion of the Parasitic Protist Giardia intestinalis
Source: PLoS One. 2011 Feb 24;6(2):e17285. doi: 10.1371/journal.pone.0017285 (PMC3044749; doi:10.1371/journal.pone.0017285)
Supplement: Figure S5 — Sequence alignment of G. intestinalis mitosomal oxidoreductase OR-1 (GL50803_91252), against G. intestinalis non-mitosomal paralogue OR-2 (GL50803_15897) and structurally related proteins containing flavodoxin-like FMN-binding domain (conserved residua in blue), FAD binding pocket (residua involved in FAD binding in green) and NADP(H) binding site (residua involved in NADP(H) in red). Saccharomyces cerevisiae Tah18, DAA11472; Homo sapiens NDOR, NADPH dependent diflavin oxidoreductase, AAH15735; Rattus norvegicus NOS, nitric oxide synthase, AAC13747; Rattus norvegicus CPR, cytochrome P450 reductase, NP_113764; Escherichia coli SiR, sulfite reductase, YP_002330508; Homo sapiens MSR, methionine synthase reductase, NP_076915; Trichomonas vaginalis Hyd, hydrogenase, TVAG_136330; Leptospira interrogans FNR, ferredoxin reductase, YP_003372. (PDF) [file pone.0017285.s005.pdf]

Fig. S5

Flavodoxin-like FMN domain

```
G.intestinalis OR-1    ------MALSIVYATVGGTARNLAHKIAA-----LLDSRGIATQLYDVKNIR-TLEDLPT 48
G.intestinalis OR-2    MTRSNKEPITVMYASATGTIQRLADQLVG-----DLCAREASVSSINMKAFAKPYNIPRG 55
S.cerevisiae Tah18     --MSSSKKIVILYGSETGNAHDFATILSH-----RLHRWHFSHTFCSIGDY-DPQDILKC 52
H.sapiens NDOR         ---MPSPQLLVLFGSQTGTAQDVSERLGR-----EARRRRLGCRVQALDSY-PVVNLINE 51
R.norvegicus NOS       -----RVRATVLFATETGKSEALARDLAA------LFSYAFNTKVVCMEQY-KANTLEEE 48
R.norvegicus CPR       -----GRNIIVFYGSQTGTAEEFANRLSKDAHRYGMRGMSADPEEYDLADL-SSLPEIDK 54
E.coli SiR            -----MPGITIISASQTGNARRVAEALRD-----DLLAAKLNVKLVNAGDY-KFKQIASE 49
H.sapiens MSR         -----MRRFLLLYATQQGQAKAIAEEICE-----QAVVHGFSADLHCISESDKYDLKTET 50
T.vaginalis Hyd       -----TSLPIVAFGSSSGTASRLARIFAS-----YFNTLPVQLNMVTMQTL-------KS 43
E.coli flavodoxin      -----MAITGIFFGSDTGNTENIAKMIQK------QLGKDVADVHDIAKSSKED-LEAYD 48

G.intestinalis OR-1    GDLLYLTSTHGLGQHPQSAHALMKSLKQQSKPGPRHWLNKREANP-QLLGSSRRIAVLGI 107
G.intestinalis OR-2    TIIYMTC-TFFAGEHPPASKE------------FIAWLQTVNPSLR--PFRDIRFAVFGM 100
S.cerevisiae Tah18     RYLFIICSTTGQGELPRNVNALKGE----RPVTFWSFLKRKNLPSN--LLNHIQTAMLGL 106
H.sapiens NDOR         PLVIFVCATTGQGDPPDNMKN------------FWRFIFRKNLPS--TALCQMDFAVLGL 97
R.norvegicus NOS       QLLLVVTSTFGNGDCPSNGQT------------LKKSLFMMKELG-----HTFRYAVFGL 91
R.norvegicus CPR       SLVVFCMATYGEGDPTDNAQD------------FYDWLQETDVD-----LTGVKFAVFGL 97
E.coli SiR            KLLIVVTSTQGEGEPPEEAVA------------LHKFLFSKKASK----LENTAFAVFSL 93
H.sapiens MSR         APLVVVVSTTGTGDPPDTARKFVK---EIQNQTLPVDFFA-----------HLRYGLLGL 96
T.vaginalis Hyd       SQIIIFCSTFGDGEFPNNAQK------------FVEMLSDSN-----EDLSHLSYGICAL 86
E.coli flavodoxin      -ILLLGIPTWYYGEAQCDWDD-----------FFPTLEE--------IDFNGKLVALFGC 88

G.intestinalis OR-1    GSI--KYHQFCAASQDAVA---VFKSHSIPTLVEPIHLDTS--ELETG-----LQLWVKK 155
G.intestinalis OR-2    GSK--NYTTFCAASKNADKSII-FGG---TRILDALHLDRDEFKSDDSA----YIHWKKD 150
S.cerevisiae Tah18     GDS--SYPKFNYGIRKLHQRIVTQLGA-NELFDRLEADDQAMAGSNKGTGL-GIESVYFE 162
H.sapiens NDOR         GDS--SYAKFNFVAKKLHRRLL-QLGG-SALLPVCLGDDQHELGPDAA-----VDPWLRD 148
R.norvegicus NOS       GSS--MYPQFCAFAHDIDQKLSH-LGA-SQLAPT--GEGDELSGQEDA-----FRSWAVQ 140
R.norvegicus CPR       GNK--TYEHFNAMGKYVDQRL-EQLGA--QRIFELGLGDDDG-NLEED-----FITWREQ 146
E.coli SiR            GDS--SYEFFCQSGKDFDSKLAE-LGG--ERLLDR-VDAD----VEYQAA---ASEWRAR 140
H.sapiens MSR         GDS--EYTYFCNGGKIIDKRLQE-LGA--RHFYDTGHADDCVG---LELV---VEPWIAG 145
T.vaginalis Hyd       GSK--DYQKYCFCGHQLDKLFVQHK--SKRLIEMVELDSS-SPDHGECLF----ELWAAK 137
E.coli flavodoxin      GDQEDYAEYFCDALGTIRDIIEPRGATIVGHWPTAGYHFEASKGLADDDHFVGLAIDEDR 148

G.intestinalis OR-1    FLTAIGTTDS-------------------------HVLDVPVRKLGEE-------NLADL 183
G.intestinalis OR-2    LFKVLGLSEQPVISTN-------------------KIIVTKNTSLPDK-------WVCDV 184
S.cerevisiae Tah18     YEKKVLSFLLSKYPNRKVNGQIIKREELDPEVYLEPASYLQLSDEHANEKFT--STKVIF 220
H.sapiens NDOR         LWDRVLGLYPPPPGLTEIPPGVPLPSKFT---LLFLQEAPSTGSEGQRVAHP--GSQEPP 203
R.norvegicus NOS       TFRAACETFDVRSKHCIQIPKRYTSNATWE----PEQYKLTQSPESLDLN----KALSSI 192
R.norvegicus CPR       FWPAVCEFFGVEATGEESSIRQYELVVH---EDMDVAKVY--TGEMGRLKSYEN-QKPPF 200
E.coli SiR            VVDALKSRAPVAAPS--------------------QSVATGTVNE---------IHTSPY 171
H.sapiens MSR         LWPALRKHFRSSRGQEEISGALPVASPASSRTDLVKSELLHIESQVELLRFDDSGRKDSE 205
T.vaginalis Hyd       CGTMLGFKMPDLAIS---------------TNYTVKVSKNPDD------IIHKEKEQPLG 176
E.coli flavodoxin      QPELTAERVEKWVKQISEELHLDEILNA-------------------------------- 176

G.intestinalis OR-1    -SENTLFDPRITESTLLSATMLSSFGWIYHLHVPSTL----------------------- 219
G.intestinalis OR-2    SPLG-YKRGIMSKVKVLSDGKVDGVVHLYEITCPCM------------------------ 219
S.cerevisiae Tah18     EGDESLKVGRVNINKRITSEGHFQDVRQFKFSNVDKI----------------------- 257
H.sapiens NDOR         SESK-PFLAPMISNQRVTGPSHFQDVRLIEFDILGS------------------------ 238
R.norvegicus NOS       HAKN-VFTMRLKSLQNLQSEKSSRTTLLVQLTFEGSR----------------------- 228
R.norvegicus CPR       DAKN-PFLAAVTANRKLNQG-TERHLMHLELDISDS-K---------------------- 235
E.coli SiR            SKDA-PLVASLSVNQKITGRNSEKDVRHIEIDLGDS------------------------ 206
H.sapiens MSR         VLK----QNAVNSNQSNVVIEDFESSLTRSVPPLSQASLNIPGLPPEYLQVHLQESLGQE 261
T.vaginalis Hyd       YEY-----GILVTSQVITP-EGFEPKMHKYQIKLPP------------------------ 206
```

```
G.intestinalis OR-1  -------------------------------------------SPMLVPGAHIAVYPRIEE 237
G.intestinalis OR-2  ---------------------------------------------KYEAGGHCAILPRNRA 235
S.cerevisiae Tah18   -------------------------------------------QENYEPGDTVTIYPCNTD 275
H.sapiens NDOR       -------------------------------------------GISFAAGDVVLIQPSNSA 256
R.norvegicus NOS     -------------------------------------------GPSYLPGEHLGIFPGNQT 246
R.norvegicus CPR     --------------------------------------------IRYESGDHVAVYPANDS 252
E.coli SiR           -------------------------------------------GLRYQPGDALGVWYQNDP 224
H.sapiens MSR        ESQVSVTSADPVFQVPISKAVQLTTNDAIKTTLLVELDISNTDFSYQPGDAFSVICPNSD 321
T.vaginalis Hyd      -------------------------------------------GVIYQTGDLVGILPENDE 224

G.intestinalis OR-1  SMINRLLDLGVLTLHDTSIDSTAFIQIDSP--HDPDLPAD---------PISIVDLLSRV 286
G.intestinalis OR-2  EDIKALLAK-GHISLDHGCKVVAEASDIVVVEHASWVRSAVNAVLPLGRPIYVLDLLSQY 294
S.cerevisiae Tah18   EDVSRFLAN-QSHWLEIADKPLNFTSG----VPNDLKDGGL------VRPMTLRNLLKYH 324
H.sapiens NDOR       AHVQRFCQV-LGLDPD-QLFMLQPRE------PDVSSPTRL------PQPCSMRHLVSHY 302
R.norvegicus NOS     GLVQGILER-VVDCSS-PDQTVCLEVLDES--GSYWVKDKR------LPPCSLRQALTYF 296
R.norvegicus CPR     ALVNQIGEI-LGADLD-VIMSLNN-LD-----EESNKKHPF------PCPTTYRTALTYY 298
E.coli SiR           ALVKELVEL-LWLKGD-EPVTVEG------------------------KTLPLNEALQWH 258
H.sapiens MSR        SEVQSLLQR-LQLEDK-REHCVLLKIKADTKKKGATLP------QHIPAGCSLQFIFTWC 373
T.vaginalis Hyd      DAVKAVLDE-LKLDPD-DIITVESSMP--------------EGYNIIPPRVTMKQLFSQY 268
```

FAD binding pocket

```
G.intestinalis OR-1  CNVNAKPSYNLISFLAPYATTSE-----------DKFKLKYLTEDAVLFD------ILSY 329
G.intestinalis OR-2  LDFSTVIDFTSFKELVPYVTDGAQ----------YRQALHMLEDPTLFKTVF------LD 338
S.cerevisiae Tah18   CDFMSIPRTSFFLKIWTFATDVTKMERGQEQLNDQREKLRQFATDQDMQDLYDY---CNR 381
H.sapiens NDOR       LDIASVPRRSFFELLACLSLHEL-----------EREKLLEFSSAQGQEELFEY---CNR 348
R.norvegicus NOS     LDITTPPTQLQLHKLARFATEET-----------HRQRLEALCQPSEYNDWK------FS 339
R.norvegicus CPR     LDITNPPRTNVLYELAQYASEPS-----------EQEHLHKMASSSGEGKE-LYLSWVVE 346
E.coli SiR           FELTVNTANIVENYATLTR---------------SETLLPLVGDKAKLQH--------YA 295
H.sapiens MSR        LEIRAIPKKAFLRALVDYTSDSAEKRRLQELCSKQGAADYSRFVRDACA----------- 422
T.vaginalis Hyd      LDLNGIPSRNLLRAFRQFCDDQFAVERLDRLLNPSDSRLFDEFVKDISISEFILEYSR-- 326
L.interrogans FRN    MHSLMKPTREPQINLFKKSNPYKAKVISNVLLTPETGTGKRPKKEGEALVHRIVLAIDHS 60

G.intestinalis OR-1  QWPTIYDFLLDFS--SLRIPLGKFLRVCPRIDPRLYSVASLPHPD-EDSNTVCTVDLLVG 386
G.intestinalis OR-2  TRLNMIDIFTKFS--SLKVPIHTLIEHMPAMTHRMYSIASAPSYVGETRLQFIVSDVDFE 396
S.cerevisiae Tah18   PRRSILEVLEDFI--SVKLPWKYVLDYLPIIKPRYYSISSG-PGD-PN-IELTVAIVKYK 436
H.sapiens NDOR       PRRTILEVLCDFPHTAAAIPPDYLLDLIPVIRPRAFSIASSLLTH-PSRLQILVAVVQFQ 407
R.norvegicus NOS     NNPTFLEVLEEFP--SLRVPAAFLLSQLPILKPRYYSISSSLDHT-PSEVHLTVAVVTYR 396
R.norvegicus CPR     ARRHILAILQDYP--SLRPPIDHLCELLPRLQARYYSIASSSKVH-PNSVHICAVAVEYE 403
E.coli SiR           ATTPIVDMVRFSP--AQLDAEALIN-LLRPLTPRLYSIASSQAEV-ENEVHVTVGVVRCD 351
H.sapiens MSR        ---CLLDLLLAFP--SCQPPLSLLLEHLPKLQPRPYSCASSSLFHPGKLHFVFNIVEFLS 477
T.vaginalis Hyd      HCKPPLDILM---------------SCIPHIWPRLYCIASA-----PTNSSVIDLIISDR 366
L.interrogans FRN    AYPYVIGQSGGVIPPGEDPEKKAKGLADVGYTVRLYSIASPSYSFGMKEDNIEFIIKRDN 120

G.intestinalis OR-1  NPKPRNGHSIQNSLGPSYLQRALISN-----------EPIRIAILQNQFLSDSGLTMLLG 435
G.intestinalis OR-2  CETLSKTIEKRPGLSTGYLSRLQEGS---------------AVFFQTFSSPVRCGDEFRG 441
S.cerevisiae Tah18   TI----LRKIRRGICTNYIARLQEGE---------------QIRYKLQNNH-IIKKEFLN 476
H.sapiens NDOR       TR----LKEPRRGLCSSWLASLDPGQ------------GPVRVPLWVRPGS-LAFPETPD 450
R.norvegicus NOS     TRD--GQGPLHHGVCSTWINNLKPED---------------PVPCFVRSVSGFQLPEDPS 439
R.norvegicus CPR     AK----SGRVNKGVATSWLRAKEPAG---------ENGGRALVPMFVRK-SQFRLPFKST 449
E.coli SiR           V-----EGRARAGGASSFLADRVEEE--------------GEVRVFIEHNDNFRLPTNPE 392
H.sapiens MSR        TATTEVLRKGVCTGWLALLVASVLQPNIHASHEDSGKALAPKISISPRTTNSFHLPDDPS 537
T.vaginalis Hyd      IFG---DGNSRNGLCTSYLKRIPEMS----------------KIALKTQHGIFYYPKNVD 407
L.interrogans FRN    IYDENGNIQF-KGVCSNYMCDLKPGD--------------EVTMTGPSGKKFLLPNTDFS 165
```

NADP(H)binding side

```
G.intestinalis OR-1   GKTALIGVAFGSGFAPFRAYHELRSAIASMDGVETLA------PYLLLLSIPHAEP--QL 487
G.intestinalis OR-2   VPSITVGLGTGLAPCRSRLQHRLALRLEQLKRAAPGEFIQPLDPYMTFIGLRRESDLKEL 501
S.cerevisiae Tah18    KPMILVGPGVGLAPLLSVVKAEISKDIK------------------LLFGCRYKDKDYIY 518
H.sapiens NDOR        TPVIMVGPGTGVAPFRAAIQERVAQGQTGNF---------------LFFGCRWRDQDFYW 495
R.norvegicus NOS      QPCILIGPGTGIAPFRSFWQQRLHDSQHRGLKG-G--------RMTLVFGCRHPEEDHLY 490
R.norvegicus CPR      TPVIMVGPGTGIAPFMGFIQERAWLREQGKE--VG--------ETLLYYGCRRSDEDYLY 499
E.coli SiR           TPVIMIGPGTGIAPFRAFMQQRAADEAPGKN--------------WLFFGNPHFTEDFLY 438
H.sapiens MSR        IPIIMVGPGTGIAPFIGFLQHREKLQEQHPDGNFG--------AMWLFFGCRHKDRDYLF 589
T.vaginalis Hyd      TPIIMVAIGCGIAPMMSMLQHRQALIYEDKLN-IG--------SASLFFGCRNKGTYPYL 458
L.interrogans FRN    GDIMFLATGTGIAPFIGMSEELLEHKLIKFTG-----------NITLVYGAPYSDELVMM 214

G.intestinalis OR-1   LEELRRDVENGVIN-VVLCLTRDDGTD--NQGKGLDIDS------FTSAHGTFLHIYKGK 538
G.intestinalis OR-2   IDELRDFVRKG-IATIFVAFSREEKSREWFEIIPNDADGHALADVDKTAFCAEYKCYVTR 560
S.cerevisiae Tah18    KDMLEDWFRKGKIALHS-SFSRDEENS-------------------------PGVKYVQD 552
H.sapiens NDOR        EAEWQELEKRDCLTLIP-AFSREQEQ----------------------------KIYVQH 526
R.norvegicus NOS      QEEMQEMVRKGVLFQVHTGYSRLPGKP---------------------------KVYVQD 523
R.norvegicus CPR      REELARFHKDGALTQLNVAFSREQAH----------------------------KVYVQH 531
E.coli SiR           QVEWQRYVKEGVLTRIDLAWSRDQKE----------------------------KIYVQD 470
H.sapiens MSR        RKELRHFLKHGILTHLKVSFSRDAPVG----------------------EEEAPAKYVQD 627
T.vaginalis Hyd      DDVLNNFVENKCLQNLFVAYSREGTT----------------------------NPYVTS 490
L.interrogans FRN    DYLKGLESKHKNFKLVT-AISREEKNSFD-----------------------GGRMYISH 250

G.intestinalis OR-1   RMGDVIHSSSIPDRLLRDLNRPDLSVYYCGPANDAVDTSNKVIENMVGAEKYRTMRSENR 598
G.intestinalis OR-2   LMGEYANNIRNFVTSKDT-CVI----TYCGKAGNVPQEIEGVILRSLVSSGMTE--KDAK 613
S.cerevisiae Tah18    YLWRLGEEITNLVVNKD--AVF----FLCGSSGKMPIQVRLTFIEMLKKWGNFSDEETAK 606
H.sapiens NDOR        RLRELGSLVWELLDRQG--AYF----YLAGNAKSMPADVSEALMSIFQEEGGLC-SPDAA 579
R.norvegicus NOS      ILQKELADEVFSVLHGEQ-GHL----YVCGDVR-MARDVATTLKKLVAAKLNLS-EEQVE 576
R.norvegicus CPR      LLKRDREHLWKLIHEGG--AHI----YVCGDARNMAKDVQNTFYDIVAEFGPME-HTQAV 584
E.coli SiR           KLREQGAELWRWINDG---AHI----YVCGDANRMAKDVEQALLEVIAEFGGMD-TEAAD 522
H.sapiens MSR        NIQLHGQQVARILLQEN--GHI----YVCGDAKNMAKDVHDALVQIISKEVGVE-KLEAM 680
T.vaginalis Hyd      ALMDRMDEVWESWKDPN--CVIM---Y-CGPPIGVPDQIKTIMVRISMKKGEMSREEAEK 544
L.interrogans FRN    RVREQ-AEAVKKILNGG--GRF----YICGGPKGMEKGVIEEIQKISGNTGTY-EEFKHH 302

G.intestinalis OR-1   VHVEAY--------------------------------- 604
G.intestinalis OR-2   ARWEKLRADQNLIFEAW---------------------- 630
S.cerevisiae Tah18    KYLKEMEKSDRYIQETW---------------------- 623
H.sapiens NDOR        AYLARLQQTRRFQTETWA--------------------- 597
R.norvegicus NOS      DYFFQLKSQKRYHEDIFGAVFSYGAKKGNTLEEPKGTRL 615
R.norvegicus CPR      DYVKKLMTKGRYSLDVWS--------------------- 602
E.coli SiR           EFLSELRVERRYQRDVY---------------------- 539
H.sapiens MSR        KTLATLKEEKRYLQDIWS--------------------- 698
T.vaginalis Hyd      FCALHP----HLFESF----------------------- 556
L.interrogans FRN    LEGAHQLFVETY--------------------------- 314
```
