# Supplementary material for: The Minimal Proteome in the Reduced Mitochondrion of the Parasitic Protist Giardia intestinalis
Source: PLoS One. 2011 Feb 24;6(2):e17285. doi: 10.1371/journal.pone.0017285 (PMC3044749; doi:10.1371/journal.pone.0017285)
Supplement: Figure S6 — Conserved glycine which is present in all GroES and Cpn10 homologues is shown in green. Hsp60 binding site is shown in yellow (van der Giezen M, León-Avila G, Tovar J. (2005) Characterization of chaperonin 10 (Cpn10) from the intestinal human pathogen Entamoeba histolytica. Microbiology 151:3107-15). Giardia intestinalis GL50803_29500; Trichomonas vaginalis TVAG_191660; Saccharomyces cerevisiae NP_014663.1; Homo sapiens XP_001118014.1; Leishmania infantum XP_001470405.1; Plasmodium falciparum PFL0740c; Arabidopsis thaliana NP_563961.1; Dictyostelium discoideum XP_636819.1; Mycobacterium tuberculosis NP_217935.1; Escherichia coli NP_290775.1. (PDF) [file pone.0017285.s006.pdf]

Fig. S6

```
Giardia                        MSLLVL GPRFLLERAV EA-----AGD VYTG--AGGL QEYVVRRVGT GVG-----R-  43
Trichomonas     MLALTSRNFAVTAATLFKPL DDRVLVKRVD R-PNKTASGI IIPDALKGKH NEATVIAVGP GHREKDGTI-  68
Saccharomyces         MSTLLKSAKSIVPL MDRVLVQRIK A-QAKTASGL YLPEKNVEKL NQAEVVAVGP GFTDANGNK-  62
Homo                    MAGQAFRKFLPL FDRVLVERSA A-ETVTKGGI MLPEKSQGKV LQATVVAVGS GSKGKGGEI-  60
Leishmania           MFRFTIPALKKLQPL GQRVLVKRVQ P-AKQTKAGI LIPEQVAAKV NEGTVVAVAA GSKD------  58
Plasmodium              MSSTITRKFIPL MDRILISKIV P-KTTTKSGL FLPESATEPS YTGKVLAVGP GRVTSNGTK-  60
Arabidopsis                 MMKRLIPT FNRILVQRVI Q-PAKTESGI LLPEKS-SKL NSGKVIAVGP GSRDKDGKL-  55
Dictyostelium             MSGVKKFIPL LDRILVEKIS NQATKTSGGI FIPTNKDAPT NNAKVIAVGT GSVKLDGSF-  59
Entamoeba                    MAKIKPT GDMVLVQHYT TQ---TVNGI ILAEQKNDKF QQGMVVSINT D-------N-  46
Escherichia                   MNIRPL HDRVIVKRKE V-ETKSAGGI VLTGSAAAKS TRGEVLAVGN GRILENGEV-  54
Mycobacterium              MAKVNIKPL EDKILVQANE A-ETTTASGL VIPDTAKEKP QEGTVVAVGP GRWDEDGEKR  58

Giardia         NYESITEGDH VLVP-ASVGQ ELQIPGIQGL VLVDEEDVLI KTDREKCLLG GFSGTFTASSH 103
Trichomonas     TPMTLQVGDR VVLA-DWSGS EVKLD-GKEF IVYREDDILA VLE                    109
Saccharomyces   VVPQVKVGDQ VLIP-QFGGS TIKLGNDDEV ILFRDAEILA KIAKD                  106
Homo            QPVSVKVGDK VLLP-EYGGT KVVLD-DKDY FLFRDGDILG KYVD                   102
Leishmania      WTPTVKVGDT VLLP-EYGGS SVKVE-GEEL FLYDESVLLG VLSS                   100
Plasmodium      ISPSVKEGDV VVLP-EYGGS SLKID-GEEF FVYRDDDIIG IIKDE                  103
Arabidopsis     IPVSVKEGDT VLLP-EYGGT QVKLG-ENEY HLFRDEDVLG TLHED                   98
Dictyostelium   IDPIVKEGDI VLINPKARSN TVPWG-DKTY HLLSENDILG IIEN                   102
Entamoeba       NPMKLKIGNH VIFG-GSPAT TFIADK-KSY SLLKQHDIFA KIE                     87
Escherichia     KPLDVKVGDI VIFNDGYGVK SEKID-NEEV LIMSESDILA IVEA                    97
Mycobacterium   IPLDVAEGDT VIYS-KYGGT EIKYN-GEEY LILSARDVLA VVSK                   100
```
