# Supplementary material for: The Minimal Proteome in the Reduced Mitochondrion of the Parasitic Protist Giardia intestinalis
Source: PLoS One. 2011 Feb 24;6(2):e17285. doi: 10.1371/journal.pone.0017285 (PMC3044749; doi:10.1371/journal.pone.0017285)
Supplement: Figure S7 — Sequence alignment of Giardia Pam16 against eukaryotic Pam 16 orthologues and giardial Pam 18 paralogue. Conserved leucin in an interacting hydrofobic pocket is shown in green (D'Silva PR, Schilke B, Hayashi M, Craig EA (2008) Interaction of the J-protein heterodimer Pam18/Pam16 of the mitochondrial import motor with the translocon of the inner membrane. Mol Biol Cell 19:424-32). The typical HPD motif (in blue) present in Pam18 is degenerated in Pam16, in yellow (Mokranjac D, Bourenkov G, Hell K, Neupert W, Groll M (2006) Structure and function of Tim14 and Tim16, the J and J-like components of the mitochondrial protein import motor. EMBO J 25:4675-85). Giardia intestinalis Pam 16 GL50803_19230; Trichomonas vaginalis TVAG_470110; Toxoplasma gondii XP_002367323.1; Saccharomyces cerevisiae NP_012431.1; Neurospora crassa XP_960477.1; Pediculus humanus XP_002428010.1; Schistosoma japonicum CAX74438.1; Homo sapiens NP_057153.8; Mus musculus NP_079847.1; Xenopus laevis NP_001084733.1; Giardia intestinalis Pam 18 XP_002364144. (PDF) [file pone.0017285.s007.pdf]

# Fig. S7

```
Giardia Pam16   MLLPKAGVEIAKGLTAGVR SSLSVLA--D NIVESSLALA DIWHHVRAEV RSL---AKAP NWAKVRIAPM  64
Trichomonas             MVIVELAGLVI SSTKVVC--S TFINGFKHAA AA-NAPNGNA FQK---FAGA VFGIQFQTRM  55
Saccharomyces           MAHRAFIQVII TGTQVFG--K AFAEAYRQAA SQ---SVKQG ATNASRR--- GTGKGEYGGI  53
Neurospora              MAYRLITQVVV VGSRVLG--R AFAEAYKQAA AS-SQYQRAQ QKNGNAA--- TGRASLTSGM  55
Pediculus               MAK-YVAQIII VGAQVIG--K AFARAVRQEL NA-SREAARR -GGGGKTGAK RAQENIRSGI  56
Schistosoma             MAK-YFVQLVI SGARVLG--R AFAQAVKEEY AS-SQRVADA RRNSTSSGSE QNTYVQNAGI  57
Homo                    MAK-YLAQIIV MGVQVVG--R AFARALRQEF AA-SRAAADA RGR---AGHR SAAASNLSGL  54
Mus                     MAK-YLAQIIV MGVQVVG--R AFARALRQEF AA-SQAAADA RGR---AGHQ SAAASNLSGL  54
Xenopus                 MAK-YLAQIVV MGMQVVG--R AFTRALRQEF AA-SKVAAEA RGR---AGTE SAAVSSLSGI  54
Giardia Pam18           MLR-VLSENRF PLSLVAGVVA GFYSYLRKDP RV-IVTSPP- RSW----K-- --GGEISSPL  50

Giardia Pam16  PLSQASQILD IESD-----T SLDKIRAQRD KL----LGQL SLSPFLQVKV NEAYERIKKS KCGRYAHRRE  125
Trichomonas    MPDEARQILG FEQKD---KL DIKSIKEHLD RMIKLNDLEK GGSPYLNERF IAASHVLAK              111
Saccharomyces  TLDESCKILN IEESKG--DL NMDKINNRFN YLFEVNDKEK GGSFYLQSKV YRAAERLKWE LAQREKNAKA  121
Neurospora     TLDEACKILN VNKPADGTAA NMEEVMERFK RLFDANDPEK GGSFYLQSKV VRARERLEAE IKPKMEEKQA  125
Pediculus      TLEEAQQILN VSKM------ DPVEIKEKYE HLFNANDKTK GGSFYLQSKV VRAKERLEQE LENMKSEKHK  120
Schistosoma    SLDEAKQILN VKDIH----- DSSTLNKQFE HLFSSNSKDK GGSFYLQSKV FRAKERIDEE LQFEKSERRR  122
Homo           SLQEAQQILN VSKL------ SPEEVQKNYE HLFKVNDKSV GGSFYLQSKV VRAKERLDEE LKIQAQEDRE  118
Mus            SLQEAQQILN VSKL------ SPEEVQKNYE HLFKVNDKSV GGSFYLQSKV VRAKERLDEE LRIQAQEDRE  118
Xenopus        SLQEAQQILN VSKL------ TPEEIQKNYE HLFKVNDKGL GGSFYLQSKV VRAKERLDQE MEIQSKTHKP  118
Giardia Pam18  SSHEARLVLN TSRFS----- TDAEVTKNYR SLLAKAHPDR GGSKYIAAII GEAHEKLRNR C            106

Giardia Pam16   SRRERCRESK IQILNKNI             143
Trichomonas                                     111
Saccharomyces  KAGDASTAKP PPNSTNSSGA DNSASSNQ   149
Neurospora     EEEVKEGW NPKIYKDR                141
Pediculus      MSQQDRKQN EKCFNIRF               137
Schistosoma    NPGSENRQSRT                      133
Homo           KGQMPHT                          125
Mus            KGQKPKT                          125
Xenopus        KEETTQT                          125
Giardia Pam18                                   106
```
