# Supplementary material for: The Minimal Proteome in the Reduced Mitochondrion of the Parasitic Protist Giardia intestinalis
Source: PLoS One. 2011 Feb 24;6(2):e17285. doi: 10.1371/journal.pone.0017285 (PMC3044749; doi:10.1371/journal.pone.0017285)
Supplement: Figure S8 — Protein sequence alignment of VAP (VAMP-associated protein) homologues. Domain structure is depicted for each represented sequence according to HHPRED (http://toolkit.tuebingen.mpg.de/). Major sperm protein domain, yellow. Coiled-coil domain in green and dimerization motif GXXXG in red. The G. intestinalis VAP contains all protein characteristics as described for human homologue. (PDF) [file pone.0017285.s008.pdf]

Fig. S8

```
G.intestinalis  ------------MNTLFSVSPMTMPFYRAYKAKQGSFFVVSSTFD----LPILVKISSTASQDYIVYPPIFVLAPHTAQE 64
D. simulans     -------------MELLSVGPQVLTFYPPYDDRQRRMVTLLNPTDRRVLFKLLLQVTSNAWLNYTVNPNTGRIAPYSSSE 67
E. cuniculi     ------------MLDIKIVPQTTIRVK----RNKRSSMFIVQNNSM---ESVGYKVKTTKPRDYIVRPNMGLIVPMQHAE 61
H. sapiens      -MASASGAMAKHEQILVLDPPTDLKFKGPFTDVVTTNLKLRNPSD----RKVCFKVKTTAPRRYCVRPNSGIIDPGSTVT 75
C. briggsae     --------MSEKHSLLQVSPHRELVFTGPFSDVVTAHMTLKNITA----NPVCFKVKTTAPKQYCVRPNSGLLKNGESKQ 68
S. pombe        ---------------MALECDSTIVFPRPLTRLVKCDLELRNTAP----YPIGFKVKTTAPKQYCVRPNGGRIEANSAVS 61
P. falciparum   ------------MKLLRVTPEKNIEFPLVHFQAVTQVVKLENVSD----KKVAFKIKTTAPNNYLVRPSFGLISVRETIE 64
T. brucei       ------------MRSKTAAPSVIVTPDRLHMTAKRKEECLVQITNIS-FEKVLFRMLTTTPERYLVKPTKGVIEPSASAS 67
P. infestans    MASSGVDVALAPNSSVILEPADSLSFHLQPQTAPQAVLTIRNVADD---RQIAFKVKTTRPLRYLVRPNQGLLGPNGSAS 77

G.intestinalis  ICVSLTL---------KEELKPHEDHRYSVDAFPLEQGAYASLSTDASPQ-PTALRIYWNSHYNDACRKGRLFTQKFTVS 134
D. simulans     IIISLKS--------FDFNTDERYNHHFVIKSMFEPQDHKN----------GQTILAIFR-----DVSRRDISSNTLLVR 124
E. cuniculi     VEVTLSE-----------DTVPDSSHKFLIEIYRFDWRRS-----------LSDFKQHLK-----SSSPKPCWTSRIGIL 114
H. sapiens      VSVMLQP--------FDYDPNEKSKHKFMVQTIFAPPN-------------TSDMEAVWK-----EAKPDELMDSKLRCV 129
C. briggsae     ITVMLQP--------LEGIPTDAGRHKFMVQSCVAPAED------------LQDLESIWK-----IVDPAELTYSKLMVT 123
S. pombe        VEVILQP------LDHEPAPGTKCRDKFLVQSTELKPELQG-----------MDIADIWT-----QVSKANISERKIRCV 119
P. falciparum   IQIILQP---------LSDKDNISNDKFQVQCLNVDDN-------------TTVDKQFWI-----TVNKNEIQDHKLIVV 117
T. brucei       VLITLSP----TTARGEDVSDVNATDDFRLEYCLQEPEDCIEPR-------CTNVPALIKE--KKQQDRRLVHSKTVRCT 134
P. infestans    IMVILQQKDCDELLRLDPAERQLANDKFLVQSIYVDDSFYELVKTKSTKEMADELTNMWA-----RTDKRALSNKKLRCR 152

G.intestinalis  FVDG--------------------APPAAVQKLARHVPAEILDSKDLD-------------------TELGNISHKKARY 175
D. simulans     LEA---------------------------QPFPLDCHGLDSLCDFSK-------------------------------- 145
E. cuniculi     YEE----------------------------------------------------------------------------- 117
H. sapiens      FEM------------PNEND-KLGITPPGNAPTVTSMSSINNTVATPASYHTKDDPRGLSVLKQEKQKNDMEPSKAVPLN 196
C. briggsae     FVD------------KKN-------PASGDDNKTFTVNGNEETFASAG------------------QAQELGSSFSAPS- 165
S. pombe        YSEGPSTANAHANAHHQPAQTTTTSIPTSATDNYTTVNGNVNQSYSKGIDGTALPSTHANPVAAPSTATTQHTQLPKTSA 199
P. falciparum   LND------------ENNSKLNHSYIPSNNVPLSEMNNKNIHNMGYVD---------------------NNNINQDDPNL 164
T. brucei       VDLT-------------------------------AVNGKWGEVRLRD-------------------------------- 151
P. infestans    FVQ------------DGEDTGRPASPPSPQRPSSTNVSPAKTTATSESRFQTRQSDEAREEVTTPMQLSSALLQDDKPKV 220

G.intestinalis  IAEKAS--------------------------LLHSIQLQQGKIDLLKNTISRDEAEILRFNREIDELQRR--------- 220
D. simulans     ----------------------------------SSVNTELQLRQLYENCANRLEEAPAKQNTPSGSRHLS--------- 182
E. cuniculi     ---------------------------------------------------EEISKEVVECSEDRG-------------- 132
H. sapiens      ASKQDG----------------------PMPKP-HSVSLNDTETRKLMEECKRLQGEMMKLSEENRHLRDEGL--RLRKV 251
C. briggsae     -SQQDG----------------------TVASLRKSLKSTIDEKEELQKKVHGLEQEIEVMLKKNRKLQQ---------- 212
S. pombe        VSHQKP-HEAPSTAVKAPTATVAENEPYPKPQSVPTTTSPNNENNALRSTANVINNTRQSTATSPSMFAGNSGNQIGLAR 278
P. falciparum   ADGLKG-----------------------------GLPGMQRKYHELLNYCVFVDKQKAALEKENESLKN---------- 205
T. brucei       ---------------------------------DGNGTGRGSKGKNVISAVLNSRKREEVPGQVKASLAGQKQ------- 191
P. infestans    AESREGGKDIQEVAALRKKYDELVAFTVQLTAQRDVLMSDLDKTRQLLQKANTDAQRVKKISEESTGLRHRKTGGASGVS 300
```

```
G.intestinalis   -LKELGNHDTLSKYIIGTRLELRVVHYILAIFVGLICGRLLHKF-- 263
D. simulans      ------KIVIIGSLTLVVLTAFVQRRLMFETFMDTQYADIHFQ--- 219
E. cuniculi      ------------------AAVLLVEMYILLNILYLFYRFFE----- 155
H. sapiens       AHSDKPGSTSTASFR---DNVTSPLPSLLVVIAAIFIGFFLGKFIL 294
C. briggsae      -------NQSDGALV---EGAFPTLQVVLIAVAALLIGLIFGHLF- 247
S. pombe         VSSSFGRPTSGAKVVPQIHNTVTVQTAFLLAIICFLIGLLF----- 319
P. falciparum    -QLKAYNSNSNKFLI---DNKLIPIIIVMLAIITKYMGYW------ 241
T. brucei        ------------------AGGGNSLMWIIGGAATLFCWWWFAY--- 216
P. infestans     GTEDSQAITSKGPKD---QGAFGPLHLLVCAIIFFLVGRYY----- 338
```
