# Supplementary material for: The Minimal Proteome in the Reduced Mitochondrion of the Parasitic Protist Giardia intestinalis
Source: PLoS One. 2011 Feb 24;6(2):e17285. doi: 10.1371/journal.pone.0017285 (PMC3044749; doi:10.1371/journal.pone.0017285)
Supplement: Figure S9 — Sequence alignment of Giardia AbcB transporter against mitochondrial and bacterial orthologs. Giardia intestinalis AbcB, GL50803_17315; Saccharomyces cerevisiae Atm1, NP_014030; Saccharomyces cerevisae Mdl1, NP_013289; Homo sapiens AbcB7, NP_004290; Homo sapiens AbcB10, NP_036221; Arabidopsis thaliana Atm3, NP_200635; Naegleria gruberi Atm1,XP_002683195; Rhodobacter sphaeroides AbcB, YP_001168064; Halobacterium sp. AbcB, NP_279266. Walker A part of a conserved ATP-binding motif in yellow; Q-loop part of a conserved ATP-binding motif in green; ABC signature, a conserved sequence specific for ABC proteins in pink; Walker B part of a conserved ATP-binding motif in blue; D-loop part of a conserved ATP-binding motif in red; H-loop part of a conserved ATP-binding motif in purple; X-loop contains a conserved arginine in AbcB transporters (•), which is not present in Giardia sequence, in cyan (Dawson RJ, Locher KP (2006) Structure of a bacterial multidrug ABC transporter. Nature 443:180-185; Bernard DG, Cheng Y, Zhao Y, Balk J (2009) An allelic mutant series of ATM3 reveals its key role in the biogenesis of cytosolic iron-sulfur proteins in Arabidopsis. Plant Physiol 151: 590-602). (PDF) [file pone.0017285.s009.pdf]

Fig S9

```
Giardia_AbcB         MLEEGGLCSAVNVPGEVNGKVGCTGGGQYEARRRASEKDGPCIYGLIVKIAKNRRKPVALTSGRTIMSKAATAPSVSEDK  80
Saccharomyces_Atm1   --------------------------------------------------------------MLLLPRCPVIGRIVRSKF  18
Saccharomyces_Mdl1   ---------------------------------------------------------------------------MIVRM   5
Homo_AbcB7           --------------------------------MALLAMHSWRWAAAAAAFEKRRHSAILIRPLVSVSGSGPQWRPHQLGA  48
Homo_AbcB10          -MRGPPAWPLRLLEPPSPAEPGRLLPVACVWAAASRVPGSLSPFTGLRPARLWGAGPALLWGVGAARRWSGCRGGGPGA  79
Arabidopsis_Atm3     ------------------------------MSRGSRFVRAPGLLLCRVNLQPQPKIPSFSYSLRSDYRLHNGFSNYIRRN  50
Naegleria_Atm1       --------------------------------------------------------------------------------   1
Rhodobacter_AbcB     --------------------------------------------------------------------------------   1
Halobacterium_AbcB   --------------------------------------------------------------------------------   1

Giardia_AbcB         SIVQHSTFRASPFHKRQRRESILTMLAILYMDAGKILLYLLLKILQSILSFWYMSTLSITINAYAIASQNTGFYRLTRRH 160
Saccharomyces_Atm1   RSGLIRNHSPVIFTVSKLSTQRPLLFNSAVNLWNQAQKDITHKKSVEQFSSAPKVKTQVKKTSKAPTLSELKILKDLFRY  98
Saccharomyces_Mdl1   IRLCKGPKLLRSQFASASALYSTKSLFKPMYQKAEINLIIPHRKRSIRLQSDIAQGKSTKPTLKLSNANSKSSGFKDIKR  85
Homo_AbcB7           LGTARAYQQIPESLKSITWQRLGKGNSGQFLDAAKALQVWPLIEKRTCWHGHAGGGLHTDPKEGLKDVDTRKIIKAMLSY 128
Homo_AbcB10          SRGVLGLARLLGLWARGPGSCRCGAFAGPGAPRLPRARFPGGPAAAAWAGDEAWRRGPAAPPGDKGRLRPAAAGLPEARK 159
Arabidopsis_Atm3     SIRTSPVINAFLSDNSPSPSPSPSPIRFVQRSSMLNGRLFSTSTPNPDQTTTKTKEIKTTSSDSDSAMADMKILRTLAGY 130
Naegleria_Atm1       --------------------------------------------------------------------------------   1
Rhodobacter_AbcB     --------------------------------------------------MSEARQRPGPTPADPPELSGWATMKRMAPY  30
Halobacterium_AbcB   -----------------------------------------------------MSTAPDNEGDDDPFEEQRADVDNAMVQ  27

Giardia_AbcB         IHQEYFKTFEEGYRAGFSWLVYRMIDRFLNQKSLAVHLSNEVVENPFG------DTVCGMEHALVYMCVQFFILSFWSFC 234
Saccharomyces_Atm1   IWPKGNNKVRIRVLIALGLLISAKILNVQVPFFFKQTIDSMN------------IAWDDPTVALPAAIGLTILCYGVAR- 165
Saccharomyces_Mdl1   LFVLSKPESKY-IGLALLLILISSSVSMAVPSVIGKLLDLASESDGEDE------EGSKSNKLYGFTKKQFFTALGAVF- 157
Homo_AbcB7           VWPKDRPDLRARVAISLGFLGGAKAMNIVVPFMFKYAVDSLNQMSGN------MLNLSDAPNTVATMATAVLIGYGVSR- 201
Homo_AbcB10          LLGLAYPERRR-LAAAVGFLTMSSVISMSAPFFLGKIIDVIYTNPTVD---------------YSDNLTRLCLGLSAVF- 222
Arabidopsis_Atm3     LWMRDNPEFRFRVIAALGFLVGAKVLNVQVPFLFKLAVDWLASATGTGASLTTFAATNPTLLTVFATPAAVLIGYGIAR- 209
Naegleria_Atm1       ----------------MGLLIGAKIFNVSVPFFFKRAVDSFGPATET-------IIQDPAVFMTMG-PISMIIGYGIAK-  55
Rhodobacter_AbcB     LWPKGQTWVKRRVIIAMVMLVLAKVISVSTPFFYKAAVDALAG------------DAPSPAFMLGLGAVGMTVAYGLAR-  97
Halobacterium_AbcB   LFDEYGRDHSFQAVVAVLASVFARVLDLAPPVLLGLAIDSVIQGNKA------FLPFLPQSVVPSSKPDRLLFMGGLIAG 101

Giardia_AbcB         YSILQAYIQRISNVIKDIINNRIYISFLSVLLTRHLSQPYQQLEQESSGERASTLNEYSQTMANLITGLLEKAIPSVSYF 314
Saccharomyces_Atm1   --FGSVLFGELRNAVFAKVAQNAIRTVSLQTFQHLMKLDLGWHLSRQTGGLTRAMDRGTKGISQVLTAMVFHIIP----- 238
Saccharomyces_Mdl1   --IIGAVANASRIIILKVTGERLVARLRTRTMKAALDQDATFLDTNRVGDLISRLSSDASIVAKSVTQNVSDGTR----- 230
Homo_AbcB7           --AGAAFFNEVRNAVFGKVAQNSIRRIAKNVFLHLHNLDLGFHLSRQTGALSKAIDRGTRGISFVLSALVFNLLP----- 274
Homo_AbcB10          --LCGAAANAIRVYLMQTSGQRIVNRLRTSLFSSILRQEVAFFDKTRTGELINRLSSDTALLGRSVTENLSDGLR----- 295
Arabidopsis_Atm3     --TGSSAFNELRTAVFSKVALRTIRSVSRKVFSHLHDLDLRYHLSRETGGLNRIIDRGSRAINFILSAMVFNVVP----- 282
Naegleria_Atm1       --TLSSLFTELRGAVFAKVTQASIRLISLGVFSKLLNMDLNFHLQRKTGALTSTMDRGKRGINFLMTSLLFNIVP----- 128
Rhodobacter_AbcB     --LGAVAFGELRDAIFVRVGQRALRQLALETFTHIHRLSLRYHISRKTGGLSRIIERGVKGVDFLLRFMLFSIGP----- 170
Halobacterium_AbcB   SFLLGAAFHWIRNWGFNSFSQHIQHRVRTDTYDKMQRLNMDFFATKQTGEMMSILSNDVNRLERFLNDGLNSAFR----- 176

Giardia_AbcB         LIMLVTTLLREKPSLNSLNDEKTNVANRKPKRKDSKKPQFSSIASTIHDRHALALILIALLPVPFNVFVVASVERQRFAL 394
Saccharomyces_Atm1   -----------------------------------ISFEISVVCGILTYQFGASFAAITFSTMLLYSIFTIKTTAWRTHF 283
Saccharomyces_Mdl1   -----------------------------------AIIQGFVGFGMMSFLS-WKLTCVMMILAPPLGAMALIYGRKIRNL 274
Homo_AbcB7           -----------------------------------IMFEVMLVSGVLYYKCGAQFALVTLGTLGTYTAFTVAVTRWRTRF 319
Homo_AbcB10          -----------------------------------AGAQASVGISMMFFVS-PNLATFVLSVVPPVSIIAVIYGRYLRKL 339
Arabidopsis_Atm3     -----------------------------------TILEISMVSGILAYKFGAAFAWITSLSVGSYIVFTLAVTQWRTKF 327
Naegleria_Atm1       -----------------------------------TMLELSIVTAIFYINYGPSFALTALGSVTLYAAWTITVTQWRTKI 173
Rhodobacter_AbcB     -----------------------------------LILELTLVAIIFAVVFGLSYMAVVVATIALYVAFTFKVTEWRVQI 215
Halobacterium_AbcB   -----------------------------------LSVMVLAIG-VYLFVVNWQLAVLTMLPVPIIALFTYRFVNAIQPK 220

Giardia_AbcB         SSSRNSAKMQSVALLTEVTSRYRLIKLYDAVSYEISNVCKLMHKFKSVDIVNILLRSTSRKVKSLTKRYTSSVLTLGSAI 474
Saccharomyces_Atm1   RRDANKADNKAASVALDSLINFEAVKYFNNEKYLADKYNGSLMNYR----------DSQIKVSQSLAFLNSGQNLIFTTA 353
Saccharomyces_Mdl1   SRQLQTSVGGLTKVAEEQLNATRTIQAYGGEKNEVRRYAKEVRNVF----------HIGLKEAVTSGLFFGSTGLVGNTA 344
Homo_AbcB7           RIEMNKADNDAGNAAIDSLLNYETVKYFNNERYEAQRYDGFLKTYE----------TASLKSTSTLAMLNFGQSAIFSVG 389
Homo_AbcB10          TKVTQDSLAQATQLAEERIGNVRTVRAFGKEMTEIEKYASKVDHVM----------QLARKEAFARAGFFGATGLSGNLI 409
Arabidopsis_Atm3     RKAMNKADNDASTRAIDSLINYETVKYFNNEGYEAEKYDQFLKKYE----------DAALQTQRSLAFLNFGQSIIFSTA 397
Naegleria_Atm1       RKQMNKAENEASGKVVDSLINYETVKYFQNEKYESEKYDEHLKKYE----------QTSLSIASSLSALNFGQTFIFSCA 243
Rhodobacter_AbcB     RRQMNEQDTDANQKAIDSLLNFETVKYFGAEQREAERYDVAMAGYE----------KAAVKTGQSLAFLNAGQSILITAG 285
Halobacterium_AbcB   YADVRSSVGHLNSRLENNLGGIQVIKTSNTERYESDRVDDVSQGYF----------DANWGAITIRIKFFPALRIISGVG 290

Giardia_AbcB         YLSLNILSYRQIQQIRYIVSTVTTQITKLLEIPGVIVDIHEHFGVIIKELSKPQEAGLSSDNTVGFAATCAYLQQHWTDI 554
Saccharomyces_Atm1   LTAMMYMGCTGVIGG-------NLTVGDLVLINQLVFQLSVPL---------------------------NFLGSVYRDL 399
Saccharomyces_Mdl1   MLSLLLVGTSMIQSG-------SMTVGELSSFMMYAVYTGSSL---------------------------FGLSSFYSEL 390
Homo_AbcB7           LTAIMVLASQGIVAG-------TLTVGDLVMVNGLLFQLSLPL---------------------------NFLGTVYRET 435
Homo_AbcB10          VLSVLYKGGLLMGSA-------HMTVGELSSFLMYAFWVGISI---------------------------GGLSSFYSEL 455
Arabidopsis_Atm3     LSTAMVLCSQGIMNG-------QMTVGDLVMVNGLLFQLSLPL---------------------------NFLGSVYRET 443
Naegleria_Atm1       LTYIMYITANKITTG-------ELTVGDLVMVNTLLFQLSIPL---------------------------NFLGTMYRET 289
Rhodobacter_AbcB     LVAVMVMAAYGVQAG-------QLTVGDFVMVNAYMIQITLPL---------------------------GFLGTVYREI 331
Halobacterium_AbcB   FVLTFVIGGIWVATGPPMFFSGTLDPGEFVTFILLSQQFIWPM---------------------------AQFGQIINMY 343
```

```
Giardia_AbcB          TRSSCKADSSFSLKAWTRSFFKRAACYINIGIIKIIPPVFRYKYPASLVTSTSPDINERMYVQELMAESQQYVTLQEPFP 634
Saccharomyces_Atm1    KQSLIDMETLFKLRKNE------------------------------------VKIKNAER--PLMLPEN---------- 431
Saccharomyces_Mdl1    MKGAGAAARVFELNDRK------------------------------------PLIRPTIGKDP-VSLAQ---------- 423
Homo_AbcB7            RQALIDMNTLFTLLKVD------------------------------------TQIKDKVMASPLQITP----------- 468
Homo_AbcB10           MKGLGAGGRLWELLERE------------------------------------PKLPFNEGVILNEKSFQ---------- 489
Arabidopsis_Atm3      IQSLVDMKSMFQLLEEK------------------------------------SDITNTSDAKPLVLKG----------- 476
Naegleria_Atm1        SQAITDIENLFTLLDSK------------------------------------NDTQDSSD-KELIIGGP---------- 322
Rhodobacter_AbcB      RQALVDMGQMFGLLGQP------------------------------------AEVTDAPDAKPLAVAG----------- 364
Halobacterium_AbcB    QRARASSERIFGLMNEP------------------------------------SRIEENPDADELVVDD----------- 376

Giardia_AbcB          LTRPIQPCTPTSIEFRDVCFSY--KTGCDILHGLTLSVHPGQKVAIVGRSGIGKSTIINLLTRLYTTPQPQDTSSPGIYL 712
Saccharomyces_Atm1    --------VPYDITFENVTFGY--HPDRKILKNASFTIPAGWKTAIVGSSGSGKSTILKLVFRFYDP------ESGRILI 495
Saccharomyces_Mdl1    ----------KPIVFKNVSFTYPTRPKHQIFKDLNITIKPGEHVCAVGPSGSGKSTIASLLLRYYDV------NSGSIEF 487
Homo_AbcB7            --------QTATVAFDNVHFEY--IEGQKVLSGISFEVPAGKKVAIVGGSGSGKSTIVRLLFRFYEP------QKGSIYL 532
Homo_AbcB10           ----------GALEFKNVHFAYPARPEVPIFQDFSLSIPSGSVTALVGPSGSGKSTVLSLLLRLYDP------ASGTISL 553
Arabidopsis_Atm3      ----------GNIEFENVHFSY--LPERKILDGISFVVPAGKSVAIVGTSGSGKSTILRMLFRFFDT------DSGNIRI 538
Naegleria_Atm1        --------TDTEIEFRNVSFYY--DPNRKILDNVSFKVKAGTTMGICGPSGSGKSTILKLIYRFYDP------TEGQIFI 386
Rhodobacter_AbcB      ----------GTVELENVHFGY--DPGRIILKGISLRVEAGETVALVGPSGSGKSTIGRLLFRFYDV------NEGAIRI 426
Halobacterium_AbcB    ----------GGVVYDDVRFGY--DDDDAIVEHIDFQVDGGDTLALVGPTGAGKSTVLKLLLRMYDV------DAGAVRV 438

Giardia_AbcB          NGRAIGSIPREELRRLITVVPQDNILIRGSATDNIAYGQPRKLPGSHAEEEQRRYIREVIRLCYEASRVAAAEELLGDAP 792
Saccharomyces_Atm1    NGRDIKEYDIDALRKVIGVVPQDTPLFNDTIWENVKFGR-----IDATDEEVITVVEKAQLAPLIKKLPQGFDTIVGERG 570
Saccharomyces_Mdl1    GDEDIRNFNLRKYRRLIGYVQQEPLLFNGTILDNILYCIPP--EIAEQDDRIRRAIGKANCTKFLANFPDGLQTMVGARG 565
Homo_AbcB7            AGQNIQDVSLESLRRAVGVVPQDAVLFHNTIYYNLLYGN-----ISASPEEVYAVAKLAGLHDAILRMPHGYDTQVGERG 607
Homo_AbcB10           DGHDIRQLNPVWLRSKIGTVSQEPILFSCSIAENIAYGADD--PSSVTAEEIQRVAEVANAVAFIRNFPQGFNTVVGEKG 631
Arabidopsis_Atm3      DGQDIKEVRLDSLRSSIGVVPQDTVLFNDTIFHNIHYGR-----LSATEEEVYEAARRAAIHETISNFPDKYSTIVGERG 613
Naegleria_Atm1        NGQDIKTVSLESLRKHIGVIPQDCVLFNDTLRHNIEYGR-----LGCSEEDIKMAASKAKLSEIIERLPKGLETPVGERG 461
Rhodobacter_AbcB      DGQDLRSVTQDSLHARIGVVPQDTVLFNDTIFYNIAYGR-----PDATPEEVEAAARAAKIHDFILRLPDGYRTMVGERG 501
Halobacterium_AbcB    DGQDISGVTLPSLRRHVGYVSQDTFLFYGTVEENITYGT-----FDADREAVVDAAKAAEAHEFIQNLPDGYDTEVGERG 513

Giardia_AbcB          YSGTLQGLSGGQRQRVGIARAVARGGSVLVLDEATSALDEETEKRVLQQLFRSLDR-NQSVVIISHRLSTLRHVDLIYIL 871
Saccharomyces_Atm1    LM-----ISGGEKQRLAIARVLLKNARIMFFDEATSALDTHTEQALLRTIRDNFTSGSRTSVYIAHRLRTIADADKIIVL 645
Saccharomyces_Mdl1    AQ-----LSGGQKQRIALARAFLLDPAVLILDEATSALDSQSEEIVAKNLQRRVER-GFTTISIAHRLSTIKHSTRVIVL 639
Homo_AbcB7            LK-----LSGGEKQRVAIARAILKDPPVILYDEATSSLDSITEETILGAMKDVVK--HRTSIFIAHRLSTVVDADEIIVL 680
Homo_AbcB10           VL-----LSGGQKQRIAIARALLKNPKILLLDEATSALDAENEYLVQEALDRLMD--GRTVLVIAHRLSTIKNANMVAVL 704
Arabidopsis_Atm3      LK-----LSGGEKQRVALARTFLKSPAILLCDEATSALDSTTEAEILNALKALAS--NRTSIFIAHRLTTAMQCDEIVVL 686
Naegleria_Atm1        LK-----LSGGEKQRTAIARAILKSPKILCCDESTSSLDSNTEKEIMKSIEELFG--KTTSIMIAHRLSTIQRADQIIVL 534
Rhodobacter_AbcB      LK-----LSGGEKQRVGIARTLLKNPPILLLDEATSALDTQTERDIQDSLREMGE--GRTVITIAHRLSTIADADRIVVL 574
Halobacterium_AbcB    VK-----LSGGQRQRIDIARAILKDPEILVLDEATSDVDTETEMLIQRSLDRLTE--DRTTFSIAHRLSTIKDADQIVVL 586

Giardia_AbcB          DNPDGSGARVVESGSYDWFVAESQYLKDAGQDISDLVSNCGCSSAGVTPGFSHATLDAKGDL---------------- 933
Saccharomyces_Atm1    DN-----GRVREEGKHLELLAMPGSLYRELWTIQEDLDHLENELKDQQEL---------------------------- 690
Saccharomyces_Mdl1    GKH----GSVVETGSFRDLIAIPNSELNALLAEQQDEEGKGGVIDLDNSVAREV------------------------ 689
Homo_AbcB7            DQ-----GKVAERGTHHGLLANPHSIYSEMWHTQSSRVQNHDNPKWEAKKENISKEEERKKLQEEIVNSVKGCGNCSC 753
Homo_AbcB10           DQ-----GKITEYGKHEELLSKPNGIYRKLMNKQS-------FISA-------------------------------- 738
Arabidopsis_Atm3      EN-----GKVVEQGPHDELLGK-SGRYAQLWTQQNSSVDMLDAAIKLE------------------------------ 728
Naegleria_Atm1        NNT----GSIAECGSHEELISKHEGIYVEMWKRQVH------------------------------------------ 566
Rhodobacter_AbcB      EE-----GRIIEEGRHEQLLAR-GGRYAAMWLRQSAEEEEAA------------------------------------ 610
Halobacterium_AbcB    ED-----GEIVERGTHADLLGD-EGLYANLWGVQAGEIDELPDEFIERAAKRQATVDDQDDD---------------- 642
```
