# Supplementary material for: The Minimal Proteome in the Reduced Mitochondrion of the Parasitic Protist Giardia intestinalis
Source: PLoS One. 2011 Feb 24;6(2):e17285. doi: 10.1371/journal.pone.0017285 (PMC3044749; doi:10.1371/journal.pone.0017285)
Supplement: Table S1 — Complete list of proteins identified by LC MS/MS in mitosomal fractions labelled by iTRAQ reagents. (PDF) [file pone.0017285.s010.pdf]

Table S1. Complete list of proteins identified by LC MS/MS in mitosomal fractions labelled by iTRAQ reagents.

MiD, mitosomal distribution. "Yes" indicates proteins with iTRAQ distribution ratio in the range of limiting markers extended from both sides of the spectrum by half distance between the limiting markers.

GiardiaDB r. 1.1		Experiment 1			Experiment 2			Experiment 3			Experiment 4		
Name	Accession number	MiD	Mascot score	Peptides	MiD	Mascot score	Peptides	MiD	Mascot score	Peptides	MiD	Mascot score	Peptides
Hypothetical protein	GL50803_10013				NO	107	2				NO	78	1
Hypothetical protein	GL50803_10016	NO	27	1	YES	265	5						
Hypothetical protein	GL50803_10079				NO	23	1						
Zinc finger protein	GL50803_101011										NO	25	1
VSP with INR	GL50803_101074	NO	488	7	NO	367	6				NO	1012	14
Dynein heavy chain	GL50803_101138				NO	70	3						
Kinesin-9	GL50803_10137										NO	26	1
Coiled-coil protein	GL50803_10167	NO	1088	18	NO	1123	18	NO	447	9	NO	58	2
Phospholipid-transporting ATPase IIB, putative	GL50803_101810				NO	35	1	NO	41	1	NO	114	2
High cysteine protein	GL50803_101832	NO	173	3	NO	164	2	NO	40	1	NO	425	6
Hypothetical protein	GL50803_102056				NO	23	1						
Kinesin-3	GL50803_102101							YES	24	1	YES	85	1
Hypothetical protein	GL50803_102184										NO	27	1
Protein 21.1	GL50803_10219										NO	23	1
Plasma membrane calcium-transporting ATPase 2	GL50803_102438				NO	87	2	NO	25	1	NO	111	2
Ornithine carbamoyltransferase	GL50803_10311	NO	360	5	NO	665	8	YES	155	2	NO	595	7
VSP, putative	GL50803_103142	NO	28	1									
Hypothetical protein	GL50803_10315	NO	103	1									
Tenascin precursor	GL50803_10330	NO	212	3	NO	330	4	NO	55	1	YES	283	4
Alpha-7.1 giardin	GL50803_103373	NO	1096	14	NO	1154	18	NO	654	11			
Alpha-9 giardin	GL50803_103437				NO	32	1						
High cysteine membrane protein Group 1	GL50803_103454	YES	270	4	NO	396	9	YES	93	2	NO	1038	14
Hypothetical protein	GL50803_103709				NO	26	1						
Protein disulfide isomerase PDI4	GL50803_103713	NO	45	1	NO	507	8				NO	605	9
Hypothetical protein	GL50803_103849				NO	27	1						
Chaperonin 60	GL50803_103891	YES	114	2	YES	336	6				YES	161	2
Hypothetical protein	GL50803_104139				NO	23	1						
Isoleucyl-tRNA synthetase	GL50803_104173	NO	36	1	NO	36	1						
Hypothetical protein	GL50803_10510							NO	21	1			
Arginyl-tRNA synthetase	GL50803_10521										NO	33	1
Hypothetical protein	GL50803_10568				NO	22	1						
Hypothetical protein	GL50803_10569				NO	33	1				NO	36	1
Hypothetical protein	GL50803_10572	NO	52	1	NO	151	2				NO	254	4
Phosphoenolpyruvate carboxykinase	GL50803_10623	NO	272	5	NO	143	3						
Histone acetyltransferase GCN5	GL50803_10666				NO	25	1						
Kinase, NEK	GL50803_10744				NO	23	1						
Splicing factor 3A subunit 2	GL50803_10755										NO	23	1
Alpha-13 giardin	GL50803_1076				NO	23	1						
Hypothetical protein	GL50803_10804										NO	35	1
Hypothetical protein	GL50803_10808										NO	34	1
WD-40 repeat protein family	GL50803_10822	NO	40	1									
Thymus-specific serine protease precursor	GL50803_10843	NO	663	8	NO	1040	15	NO	365	5	NO	171	3
Alpha-snap	GL50803_10856	NO	174	3	NO	374	7				NO	25	1
Hypothetical protein	GL50803_10879							NO	24	1			
4-alpha-glucanotransferase, amylo-alpha-1,6-glucosidase	GL50803_10885										NO	64	2
Hypothetical protein	GL50803_10943				NO	23	1						
Protein 21.1	GL50803_11099										NO	31	1
Hypothetical protein	GL50803_11105	NO	24	1									
Enolase	GL50803_11118	NO	308	6	NO	478	9	NO	50	1			
Hypothetical protein	GL50803_11129	NO	23	1									
Hypothetical protein	GL50803_11131				NO	23	1						
Protein 21.1	GL50803_11165										NO	32	1
Hypothetical protein	GL50803_11170	NO	23	1									
Hypothetical protein	GL50803_111809	NO	32	1	YES	34	1				NO	23	1
VSP	GL50803_111873				NO	22	1						
Hypothetical protein	GL50803_11196				NO	34	1						
Hypothetical protein	GL50803_111973	NO	156	3	NO	120	2				NO	193	4
Alpha-tubulin	GL50803_112079	NO	185	3	NO	358	5	YES	68	1	NO	394	7
Hypothetical protein	GL50803_112080				NO	23	1				NO	25	1
Arginine deiminase	GL50803_112103	NO	123	2									
VSP with INR	GL50803_112208	NO	220	2							NO	68	2
Elongation factor 1-alpha	GL50803_112312	YES	424	10	NO	346	7	NO	312	7	NO	408	9

GiardiaDB r. 1.1			Experiment 1			Experiment 2			Experiment 3			Experiment 4	
Name	Accession number	MiD	Mascot score	Peptides	MiD	Mascot score	Peptides	MiD	Mascot score	Peptides	MiD	Mascot score	Peptides
Hypothetical protein	GL50803_11237	YES	22	1							NO	24	1
Ribosomal protein L13a	GL50803_11247										NO	40	1
Hypothetical protein	GL50803_112489	NO	48	1	NO	66	1	NO	21	1	NO	163	3
High cysteine membrane protein VSP-like	GL50803_112584										NO	164	3
High cysteine membrane protein EGF-like	GL50803_112633	NO	258	5									
Hypothetical protein	GL50803_112784	NO	30	1	NO	34	1				NO	30	1
High cysteine membrane protein Group 4	GL50803_112828	NO	366	5	NO	137	2				NO	738	11
Kinesin-3	GL50803_112846	NO	115	3	NO	22	1						
Hypothetical protein	GL50803_112938	NO	153	3									
Chromodomain helicase-DNA-binding protein, putative	GL50803_112978										NO	25	1
Amino acid transporter, putative	GL50803_11299	NO	182	3	NO	154	3				NO	192	3
Hypothetical protein	GL50803_113130										NO	195	3
Hypothetical protein	GL50803_113133	NO	659	12	NO	805	14	NO	312	7	NO	1484	24
High cysteine membrane protein EGF-like	GL50803_113213	NO	161	4	NO	227	5						
Hypothetical protein	GL50803_113219				NO	22	1				NO	51	2
Msh2-like protein	GL50803_113252				NO	23	1						
Hypothetical protein	GL50803_11328	NO	22	1									
Kinase, AGC MAST	GL50803_113522	NO	27	1									
High cysteine membrane protein EGF-like	GL50803_113531	NO	293	5	NO	255	5				NO	436	8
Ribosomal protein S4	GL50803_11359	YES	29	1	NO	31	1						
Hypothetical protein	GL50803_113625							NO	26	1			
Cysteine protease	GL50803_113656				YES	73	2						
Coiled-coil protein	GL50803_113677	NO	2628	43	NO	1324	24	NO	695	14	NO	21	1
Hypothetical protein	GL50803_113723	NO	111	2	NO	172	3				NO	239	4
ABC transporter, ATP-binding protein, putative	GL50803_113876										NO	30	1
Long chain fatty acid CoA ligase, putative	GL50803_113892	NO	153	2							YES	224	4
Kinase, NEK	GL50803_11390	NO	495	8	NO	393	7	NO	46	2	NO	54	1
High cysteine membrane protein Group 3	GL50803_113987	NO	179	4	NO	221	4				NO	392	7
High cysteine membrane protein Group 4	GL50803_114042							YES	28	1	NO	330	5
Alpha-7.2 giardin	GL50803_114119	NO	1146	14	NO	1180	17	NO	885	14			
Hypothetical protein	GL50803_114210	NO	135	1							NO	174	3
High cysteine membrane protein Group 6	GL50803_114470				NO	22	1				NO	23	1
Hypothetical protein	GL50803_114498				NO	23	1				NO	23	1
Pyruvate-flavodoxin oxidoreductase	GL50803_114609	NO	46	1	NO	44	1	NO	22	1	NO	55	1
Hypothetical protein	GL50803_114623	NO	83	2	NO	30	1	NO	47	1	NO	168	3
High cysteine membrane protein EGF-like	GL50803_114626	NO	332	5	NO	283	6				NO	536	10
Hypothetical protein	GL50803_114674										NO	43	1
VSP with INR	GL50803_11470	YES	220	3									
NSF	GL50803_114776	NO	24	1									
Hypothetical protein	GL50803_114777	NO	165	3	YES	140	3				NO	658	8
Alpha-7.3 giardin	GL50803_114787	NO	1191	15	NO	1230	19	NO	970	15	NO	230	4
VSP	GL50803_114813	NO	43	1							NO	43	1
Tenascin precursor	GL50803_114815	NO	84	2	NO	71	2						
High cysteine membrane protein VSP-like	GL50803_114852										NO	189	4
Cysteine protease	GL50803_114915	NO	218	4							NO	442	8
Reverse transcriptase	GL50803_114973										NO	26	1
High cysteine membrane protein EGF-like	GL50803_114991										NO	93	2
Multidrug resistance-associated protein 1	GL50803_115052	NO	106	2									
High cysteine membrane protein VSP-like	GL50803_115066	NO	127	2	NO	84	3				NO	600	9
High cysteine protein	GL50803_115158	NO	25	1									
Hypothetical protein	GL50803_115159	NO	494	8	NO	528	9	NO	313	3	NO	1144	15
VSP	GL50803_11521	YES	198	3							NO	31	1
Coiled-coil protein	GL50803_115245				NO	36	1				NO	24	1
Hexose transporter	GL50803_11540	NO	378	4	NO	484	6	NO	225	2	NO	605	7
Hypothetical protein	GL50803_11557	YES	30	1	NO	41	1				NO	40	1
Lysophosphatidic acid phosphatase	GL50803_11599	NO	88	2	NO	52	2	NO	24	1			
Hypothetical protein	GL50803_11640	NO	31	1									
Alpha-1 giardin	GL50803_11654	NO	753	11	NO	934	17	YES	198	5	NO	238	5
Alpha-3 giardin	GL50803_11683	NO	648	9	NO	701	10	NO	394	6	NO	61	1
Leucine-rich repeat protein	GL50803_11684	NO	371	6	NO	523	9	NO	115	2	NO	884	14
Hypothetical protein	GL50803_11720	NO	657	9	NO	533	9	NO	207	2	NO	605	10
Hypothetical protein	GL50803_11749										NO	49	1
Hypothetical protein	GL50803_11752										NO	39	1
Hypothetical protein	GL50803_11772				NO	37	1				NO	155	3
Kinase, NEK-frag	GL50803_11775				NO	23	1				YES	50	2
Hypothetical protein	GL50803_11866										YES	25	1

GiardiaDB r. 1.1			Experiment 1			Experiment 2			Experiment 3			Experiment 4	
Name	Accession number	MiD	Mascot score	Peptides	MiD	Mascot score	Peptides	MiD	Mascot score	Peptides	MiD	Mascot score	Peptides
Coatomer alpha subunit	GL50803_11953				YES	31	1						
Hypothetical protein	GL50803_12035				NO	73	1				NO	167	2
Variant-specific surface protein	GL50803_12063				NO	35	1						
Elongation factor 1-gamma	GL50803_12102	YES	126	3	NO	89	2	YES	59	1	NO	158	3
Protein 21.1	GL50803_12139							NO	21	1			
Hypothetical protein	GL50803_12213	NO	22	1	NO	23	1				NO	23	1
Hypothetical protein	GL50803_12217	NO	32	1	NO	35	1						
Hypothetical protein	GL50803_12224	NO	30	1									
Hypothetical protein	GL50803_12830										NO	99	2
Hypothetical protein	GL50803_12999	NO	321	3	NO	329	3	YES	130	2	YES	414	5
Vacuolar ATP synthase subunit d	GL50803_13000	NO	198	4	NO	209	4	YES	85	3	NO	342	5
VSP AS8	GL50803_13194										NO	33	1
ATP-dependent RNA helicase	GL50803_13220				NO	25	1						
Dynein light intermediate chain	GL50803_13273	NO	30	1	NO	36	1						
Hypothetical protein	GL50803_13288	YES	35	1									
Alcohol dehydrogenase lateral transfer candidate	GL50803_13350	NO	63	2				NO	93	1			
T-complex protein-10	GL50803_13352							NO	24	1			
Hypothetical protein	GL50803_13413	NO	95	2	YES	60	2						
Ribosomal protein L10a	GL50803_1345				NO	54	1				NO	56	1
Axoneme-associated protein GASP-180	GL50803_13475	NO	68	2	NO	26	1						
Hypothetical protein	GL50803_13555										NO	29	1
Translation elongation factor	GL50803_13561	YES	36	1									
Beta tubulin	GL50803_136020	NO	239	5	NO	729	11	YES	68	2	NO	841	13
Hypothetical protein	GL50803_13603	NO	25	1									
Hypothetical protein	GL50803_1376							YES	29	1			
TMP52	GL50803_137603	NO	473	7	NO	552	9	NO	169	3	NO	893	13
VSP with INR	GL50803_137608				NO	810	9	NO	498	11			
VSP	GL50803_137612				NO	204	4				NO	311	5
VSP AS12	GL50803_137614	NO	31	1									
VSP	GL50803_137617	NO	246	3	NO	256	3				NO	517	6
VSP	GL50803_137618				NO	259	5	YES	67	1	NO	530	9
TMP55	GL50803_137641	NO	261	4	NO	445	6	NO	81	2	NO	761	10
High cysteine membrane protein VSP-like	GL50803_137672				NO	22	1						
Hypothetical protein	GL50803_137685	NO	32	1	YES	131	2				YES	200	4
Hypothetical protein	GL50803_137689				NO	41	1						
Kinase, NEK	GL50803_137706										NO	24	1
High cysteine membrane protein Group 5	GL50803_137715				NO	32	1				NO	36	1
Axoneme-associated protein GASP-180	GL50803_137716	NO	550	14	NO	200	6	NO	321	10			
High cysteine protein	GL50803_137732	NO	28	1									
SMC3-like protein	GL50803_137745				NO	28	1				NO	28	1
Hypothetical protein	GL50803_137746				NO	25	1				YES	23	1
Hypothetical protein	GL50803_137747							NO	32	1			
Hypothetical protein	GL50803_137754				NO	26	1						
Hypothetical protein	GL50803_13838				NO	23	1						
Hypothetical protein	GL50803_13922	YES	236	2	NO	373	6				NO	1121	14
Hypothetical protein	GL50803_13924				NO	23	1						
Hypothetical protein	GL50803_13945				NO	31	1						
Hypothetical protein	GL50803_14003										NO	29	1
Long-flagella protein, kinase, CMGC RCK	GL50803_14004				NO	40	1						
High cysteine membrane protein Group 2	GL50803_14017				NO	176	5				NO	343	5
Cathepsin B precursor	GL50803_14019	NO	528	5	NO	608	8	NO	405	5	NO	256	2
Hypothetical protein	GL50803_14164				YES	23	1						
Amino acid transporter system N2, putative	GL50803_14168							NO	37	1	NO	30	1
Molybdenum cofactor sulfurase	GL50803_14200				YES	56	1				NO	43	1
Kinase, NEK	GL50803_14223	NO	61	2	YES	124	2	NO	94	2	YES	33	1
CXC-rich protein	GL50803_14225	NO	379	7	NO	174	4	YES	63	1	NO	494	8
Hypothetical protein	GL50803_14242				NO	25	1						
Hypothetical protein	GL50803_14252				NO	23	1						
Hypothetical protein	GL50803_14278				NO	28	1				YES	31	1
Tenascin-X	GL50803_14360										NO	28	1
Dynamin	GL50803_14373	NO	510	10	NO	841	12	NO	183	4	NO	1227	16
Protein 21.1	GL50803_14434	NO	154	2									
Synaptobrevin-like protein	GL50803_14469	YES	45	1	NO	45	1				NO	36	1
Cysteine desulfurase	GL50803_14519	YES	296	4	YES	100	2	YES	52	1	YES	168	3
Hypothetical protein	GL50803_14543										NO	24	1
Alpha-6 giardin	GL50803_14551	NO	297	4	NO	86	1						

GiardiaDB r. 1.1			Experiment 1			Experiment 2			Experiment 3			Experiment 4	
Name	Accession number	MiD	Mascot score	Peptides	MiD	Mascot score	Peptides	MiD	Mascot score	Peptides	MiD	Mascot score	Peptides
Dipeptidyl-peptidase I precursor	GL50803_14566	NO	260	4	NO	271	6	NO	149	3	NO	449	6
Tenascin-X	GL50803_14573	NO	167	3	NO	96	2						
Chaperone protein DnaK HSP70	GL50803_14581	YES	345	5	YES	404	7	YES	130	5	NO	86	2
Hypothetical protein	GL50803_14637				NO	24	1						
Hypothetical protein	GL50803_14660	YES	105	2									
Protein disulfide isomerase PDI3	GL50803_14670				NO	69	1				YES	60	1
RRNA biogenesis protein RRP5	GL50803_14702	NO	26	1									
Kinase, NEK	GL50803_14742	NO	28	1									
hemagglutinin protein-like protein	GL50803_14802							NO	25	1			
HesB domain-containing protein	GL50803_14821	NO	132	3	YES	198	3	YES	21	1	YES	104	2
Hypothetical protein	GL50803_14845	YES	47	1	NO	69	2				YES	46	1
Protein 21.1	GL50803_14859				NO	22	1						
Hypothetical protein	GL50803_14863	NO	25	1									
Hypothetical protein	GL50803_14874				NO	25	1						
Hypothetical protein	GL50803_14939	YES	55	1	NO	90	2	NO	114	2	YES	122	2
Vacuolar ATP synthase subunit H	GL50803_14961										NO	31	1
Cathepsin L precursor	GL50803_14983	NO	366	8	NO	516	9	NO	101	2	NO	23	1
Pyrophosphate-fructose 6-phosphate 1-phosphotransferase	GL50803_14993	YES	56	1									
Hypothetical protein	GL50803_14994										NO	24	1
Hypothetical protein	GL50803_15011							NO	30	1			
Kinase, NEK-like	GL50803_15035	NO	136	3	NO	173	4	NO	36	1			
Hypothetical protein	GL50803_15084				YES	22	1						
Alpha-14 giardin	GL50803_15097	NO	548	9	NO	643	8	YES	113	1	NO	271	4
NifU-like protein	GL50803_15196	YES	167	3	YES	243	5	YES	23	1	YES	89	2
ERP3	GL50803_15204				YES	29	1				YES	95	2
WD-40 repeat protein	GL50803_15218										NO	23	1
Grp94	GL50803_15247				NO	62	2						
Hypothetical protein	GL50803_15289	NO	26	1	NO	25	1				NO	98	2
Hypothetical protein	GL50803_15292				NO	39	1				NO	46	1
Zinc finger protein, putative	GL50803_15295				NO	26	1						
High cysteine membrane protein Group 1	GL50803_15317	NO	279	6	NO	578	11	NO	67	1	NO	687	10
Hypothetical protein	GL50803_15343							NO	21	1			
Hypothetical protein	GL50803_15347	NO	114	1									
Peroxiredoxin 1	GL50803_15383				NO	315	5						
Replication factor C, subunit 1	GL50803_15392	NO	23	1									
Chaperone protein dnaJ	GL50803_15398				NO	144	4	NO	21	1	NO	103	2
Kinase, NEK	GL50803_15409	NO	673	9	NO	958	13	NO	176	4	NO	107	2
Kinase, NEK-frag	GL50803_15411	NO	731	10	NO	729	11	NO	372	5	NO	112	2
Hypothetical protein	GL50803_15461										NO	48	1
Protein 21.1	GL50803_15476				NO	26	1						
Hypothetical protein	GL50803_15499										NO	22	1
Rab2a	GL50803_15567				NO	27	1				NO	30	1
Alanyl dipeptidyl peptidase	GL50803_15574	NO	248	3	NO	388	6	NO	246	3			
Aminoacyl-histidine dipeptidase	GL50803_15832										NO	27	1
Serine peptidase, putative	GL50803_15871	NO	661	11	NO	766	11	NO	132	2	NO	190	3
UDP-N-acetylglucosamine-dolichyl-phosphateN-acetylgluco	GL50803_15889										YES	36	1
Hypothetical protein	GL50803_15918				NO	254	3						
Protein 21.1	GL50803_15965										NO	27	1
Hypothetical protein	GL50803_15985				YES	35	1						
Nucleotide-binding head-stalk protein, putative	GL50803_15995				NO	24	1				NO	30	1
Spindle pole protein, putative	GL50803_16013										NO	22	1
Hypothetical protein	GL50803_16039	NO	27	1							NO	22	1
Hypothetical protein	GL50803_16044										NO	31	1
Hypothetical protein	GL50803_16054				NO	23	1				NO	26	1
Peroxiredoxin 1	GL50803_16076	NO	217	4	NO	293	5	YES	56	2	NO	279	5
Hypothetical protein	GL50803_16135				NO	23	1						
Coiled-coil protein	GL50803_16152				YES	57	2						
Cathepsin B precursor	GL50803_16160	NO	333	3	NO	281	3	NO	226	2	NO	393	4
Kinase, NEK	GL50803_16167				NO	22	1						
Protein 21.1	GL50803_16220							NO	22	1	NO	24	1
Ribosomal protein S3a	GL50803_16265				NO	42	1				NO	51	1
Hypothetical protein	GL50803_16267				NO	21	1	NO	20	1			
Hypothetical protein	GL50803_16273				NO	24	1						
Sda1, severe depolymerization of actin	GL50803_16299				NO	27	1				NO	36	1
Protein 21.1	GL50803_16300				NO	22	1				NO	26	1
Hypothetical protein	GL50803_16313				YES	24	1						

GiardiaDB r. 1.1		Experiment 1			Experiment 2			Experiment 3			Experiment 4		
Name	Accession number	MiD	Mascot score	Peptides	MiD	Mascot score	Peptides	MiD	Mascot score	Peptides	MiD	Mascot score	Peptides
High cysteine membrane protein Group 1	GL50803_16318	NO	130	2	NO	72	2				NO	129	2
Neurogenic locus Notch protein precursor	GL50803_16322	NO	359	5	NO	604	8	NO	69	1	NO	209	3
Coiled-coil protein	GL50803_16332				NO	21	1				NO	21	1
Protein 21.1	GL50803_16354				YES	25	1						
Protein 21.1	GL50803_16355				NO	35	1				NO	35	1
Gamma adaptin	GL50803_16364	NO	83	1	NO	45	1	NO	70	1	NO	52	2
Hypothetical protein	GL50803_16367				NO	22	1				NO	30	1
Transcriptional activator, putative	GL50803_16370	NO	52	2	NO	30	1				NO	23	1
ATP-dependent RNA helicase p47, putative	GL50803_16376							NO	46	1			
Cathepsin L precursor	GL50803_16380	NO	228	4	NO	408	7				NO	138	2
Hypothetical protein	GL50803_16404	NO	64	1	NO	56	1				NO	76	1
Hypothetical protein	GL50803_16424	NO	117	3	YES	113	3	NO	52	2	NO	88	3
Hypothetical protein	GL50803_16430	YES	32	1	NO	30	1				NO	30	1
Carbamate kinase	GL50803_16453	NO	67	2				NO	95	2			
Protein kinase, putative	GL50803_16454	NO	27	1	NO	105	2				NO	137	3
Cathepsin B precursor	GL50803_16468	NO	149	2	NO	103	2						
Tenascin-37	GL50803_16477	YES	40	1	NO	178	4	NO	22	1			
Hypothetical protein	GL50803_16484										NO	22	1
Hypothetical protein	GL50803_16492										NO	24	1
VSP with INR	GL50803_16501							NO	54	2			
Hypothetical protein	GL50803_16502				NO	24	1						
Hypothetical protein	GL50803_16507	NO	881	15	NO	1446	21	NO	356	6	NO	1919	26
Ribosomal protein L3	GL50803_16525				NO	41	1						
Dynein regulatory complex	GL50803_16540										NO	46	2
Hypothetical protein	GL50803_16544										NO	30	1
IFT complex A	GL50803_16547	NO	22	1									
ABC transporter family protein	GL50803_16575	NO	24	1	NO	23	1				NO	444	7
Hypothetical protein	GL50803_16585				NO	25	1						
Hypothetical protein	GL50803_16588							NO	21	1			
ABC transporter family protein	GL50803_16592	NO	252	5	NO	260	5	NO	93	2	NO	769	12
Liver stage antigen-like protein	GL50803_16595							NO	30	1			
Hypothetical protein	GL50803_16596	NO	119	1	NO	177	3						
Hypothetical protein	GL50803_16599				NO	22	1						
Hypothetical protein	GL50803_16601				NO	22	1						
Hypothetical protein	GL50803_16602										NO	25	1
Hypothetical protein	GL50803_16604										NO	47	2
Hypothetical protein	GL50803_16648				NO	28	1				NO	27	1
Hypothetical protein	GL50803_16677				NO	21	1						
Hypothetical protein	GL50803_16686				NO	23	1						
High cysteine membrane protein Group 5	GL50803_16716	NO	320	7	NO	280	7	NO	28	1	NO	697	11
High cysteine membrane protein Group 2	GL50803_16721										NO	281	4
Axoneme-associated protein GASP-180	GL50803_16745	NO	563	11	NO	491	10	NO	196	4	NO	34	1
Hypothetical protein	GL50803_16751	NO	26	1									
Phenylalanyl-tRNA synthetase beta chain	GL50803_16760				NO	23	1						
Hypothetical protein	GL50803_16761				NO	24	1	NO	26	1	NO	23	1
Cathepsin B precursor	GL50803_16779	NO	457	7	NO	301	4	NO	163	3	NO	115	2
Dynein heavy chain	GL50803_16804										NO	22	1
Kinase, NEK	GL50803_16824	NO	37	1	NO	51	1	YES	87	1	NO	45	2
Tenascin-like	GL50803_16833				YES	96	2						
Kinase, STE Dicty2	GL50803_16834				NO	21	1						
High cysteine membrane protein Group 2	GL50803_16842										NO	53	1
Hypothetical protein	GL50803_16844				NO	231	4				NO	32	1
Hypothetical protein	GL50803_16861				NO	27	1						
Hypothetical protein	GL50803_16888				NO	83	2						
Phosphatidate cytidylyltransferase	GL50803_16906										YES	48	2
Hypothetical protein	GL50803_16910				NO	41	1						
Hypothetical protein	GL50803_16916	NO	355	7	NO	270	5	NO	54	1	NO	178	3
Tetratricopeptide repeat protein	GL50803_16934										NO	23	1
High cysteine membrane protein EGF-like	GL50803_16936	NO	29	1									
Hypothetical protein	GL50803_16949	NO	22	1							NO	23	1
Hypothetical protein	GL50803_16963	NO	31	1	NO	28	1				NO	34	1
FtsJ cell division protein, putative	GL50803_16993				YES	24	1						
Hypothetical protein	GL50803_16998				YES	24	1				NO	24	1
Peptidyl-prolyl cis-trans isomerase B precursor	GL50803_17000				NO	39	1						
Zinc finger domain	GL50803_17003										NO	23	1
Hypothetical protein	GL50803_17006				NO	36	1						

GiardiaDB r. 1.1		Experiment 1			Experiment 2			Experiment 3			Experiment 4		
Name	Accession number	MiD	Mascot score	Peptides	MiD	Mascot score	Peptides	MiD	Mascot score	Peptides	MiD	Mascot score	Peptides
Spindle pole protein, putative	GL50803_17058				NO	23	1						
Hypothetical protein	GL50803_17089							NO	21	1			
Protein 21.1	GL50803_17097										NO	23	1
Vacuolar protein sorting 11	GL50803_17109				NO	40	1				NO	93	2
U2 small nuclear ribonucleoprotein A', putative	GL50803_17118										NO	23	1
Bip	GL50803_17121	NO	448	8	NO	1626	24	NO	111	2	YES	1250	19
Kinase, CMGC CMGC-GL1	GL50803_17139	NO	44	1									
Alpha-11 giardin	GL50803_17153	NO	699	10	NO	952	14	NO	429	7	NO	172	4
Phosphatase subunit gene g4-1	GL50803_17157	NO	33	1									
ABC transporter, ATP-binding protein	GL50803_17165	YES	64	1	NO	81	2				NO	113	2
Hypothetical protein	GL50803_17166										NO	27	1
Leucine-rich repeat protein	GL50803_17198	NO	196	3	NO	271	5	NO	82	1	NO	366	7
5' nucleotidase family protein	GL50803_17205				NO	124	3				NO	325	5
Alpha-SNAP, putative	GL50803_17224	NO	585	7	NO	392	5	NO	49	1			
Gamma giardin	GL50803_17230				NO	1260	16				NO	601	9
Hypothetical protein	GL50803_17236	YES	69	1							NO	29	1
Coiled-coil protein	GL50803_17249	NO	4704	71	NO	4520	78	NO	2399	40	NO	241	5
Hypothetical protein	GL50803_17253										NO	22	1
Phosphomannomutase-2	GL50803_17254	NO	134	3	NO	60	2	NO	31	1			
Phospholipase B	GL50803_17277	NO	149	3	NO	182	5	NO	174	3	NO	629	9
Hypothetical protein	GL50803_17283	NO	45	1							NO	45	1
Protein 21.1	GL50803_17285										NO	26	1
Protein 21.1	GL50803_17288	NO	23	1	NO	54	2	YES	28	1	NO	38	1
Hypothetical protein	GL50803_17296				YES	29	1						
Kinase, NEK-like	GL50803_17299										NO	24	1
Coiled-coil protein	GL50803_17320				NO	29	1						
Xaa-Pro dipeptidase	GL50803_17327	NO	606	9	NO	440	8	NO	82	2			
High cysteine membrane protein Group 2	GL50803_17328				YES	56	2				NO	113	3
Hypothetical protein	GL50803_17342	NO	151	3	YES	149	3						
Hypothetical protein	GL50803_17385										NO	22	1
Hypothetical protein	GL50803_17396										NO	23	1
Hypothetical protein	GL50803_17404							NO	28	1			
TCP-1 chaperonin subunit gamma	GL50803_17411	YES	22	1							NO	24	1
Heat shock protein 70	GL50803_17432				NO	54	1				NO	31	1
ATP-dependent DNA helicase recQ	GL50803_17438	NO	32	1	NO	33	1				NO	24	1
Hypothetical protein	GL50803_17468				NO	27	1						
CXC-rich protein	GL50803_17476	YES	116	3	NO	111	3				NO	255	7
Kinase, NEK	GL50803_17510				YES	25	1						
Hypothetical protein	GL50803_17530										NO	77	3
Coiled-coil protein	GL50803_17574										NO	23	1
Kinase, NEK-frag	GL50803_17578	NO	32	1	NO	28	1						
Hypothetical protein	GL50803_17593				NO	25	1						
Kinase, NEK	GL50803_17622				NO	28	1				NO	28	1
Vacuolar proton-ATPase subunit, putative	GL50803_18470	NO	296	4	NO	314	6	YES	27	1	NO	608	8
Hypothetical protein	GL50803_19230	YES	35	1									
Hypothetical protein	GL50803_1937	YES	51	2	NO	58	2				NO	75	2
Glutaredoxin-related protein	GL50803_2013	YES	67	1	YES	249	3	YES	54	2	YES	84	1
Kinase, CMGC CMGC-GL1	GL50803_21116				NO	23	1						
Long chain fatty acid CoA ligase 5	GL50803_21118				YES	25	1						
Hypothetical protein	GL50803_21234										NO	28	1
High cysteine membrane protein Group 5	GL50803_21321	NO	127	2	NO	150	3				NO	509	10
ABC transporter, ATP-binding protein	GL50803_21411	NO	249	5	NO	369	7	YES	110	2	NO	429	10
Spindle pole protein, putative	GL50803_21444				YES	63	2						
Hypothetical protein	GL50803_21474				NO	30	1						
Protein 21.1	GL50803_21603				NO	24	1						
Hypothetical protein	GL50803_21628	NO	26	1									
Coiled-coil protein	GL50803_21662				NO	31	1						
NADP-specific glutamate dehydrogenase	GL50803_21942	NO	30	1									
Hypothetical protein	GL50803_22136	NO	47	1	NO	25	1				NO	177	3
Hypothetical protein	GL50803_221692				NO	29	1						
Hypothetical protein	GL50803_221693	NO	1461	15	NO	1508	16	NO	1095	17	NO	1195	14
Hypothetical protein	GL50803_22291	NO	308	4	NO	199	3	NO	234	3	NO	254	4
Hypothetical protein	GL50803_22394	NO	26	1									
Hypothetical protein	GL50803_22573				NO	26	1				NO	23	1
Hypothetical protein	GL50803_23389										YES	33	1
Protein 21.1	GL50803_23492	YES	130	1									

GiardiaDB r. 1.1			Experiment 1			Experiment 2			Experiment 3			Experiment 4	
Name	Accession number	MiD	Mascot score	Peptides	MiD	Mascot score	Peptides	MiD	Mascot score	Peptides	MiD	Mascot score	Peptides
Hypothetical protein	GL50803_2352										NO	23	1
Vacuolar protein sorting 35	GL50803_23833				NO	26	1	YES	22	1			
Protein 21.1	GL50803_24842										NO	25	1
High cysteine membrane protein Group 2	GL50803_24880	NO	211	3	NO	303	4	NO	48	1	NO	590	9
Hypothetical protein	GL50803_25205	NO	110	3	NO	81	1				NO	130	2
High cysteine protein	GL50803_25238	NO	49	1	NO	27	1				NO	383	6
Kinase, NEK	GL50803_26199	NO	101	3	NO	99	2				NO	37	1
Hypothetical protein	GL50803_2692				NO	24	1						
[2Fe-2S] ferredoxin	GL50803_27266	YES	182	2				YES	135	1			
High cysteine membrane protein Group 3	GL50803_27717	NO	281	5	NO	270	5	NO	28	1	NO	528	9
Hypothetical protein	GL50803_28337							NO	22	1			
Multidrug resistance-associated protein 1	GL50803_28379	YES	71	1	NO	122	3	NO	30	1	NO	210	4
Hypothetical protein	GL50803_28433										NO	24	1
Dipeptidyl-peptidase I precursor	GL50803_28651										NO	112	2
Hypothetical protein	GL50803_28699										NO	62	1
Hypothetical protein	GL50803_28962	YES	39	1									
Hypothetical protein	GL50803_2906				NO	29	1						
Hypothetical protein	GL50803_29327	NO	111	2				YES	65	1			
Hypothetical protein	GL50803_29486							NO	21	1			
Protein disulfide isomerase PDI1	GL50803_29487	NO	153	2	NO	322	4	NO	58	1	NO	278	4
Copine I	GL50803_29490	NO	55	1							NO	29	1
Hypothetical protein	GL50803_29500	YES	68	1	NO	57	1	NO	24	1			
Hypothetical protein	GL50803_3021							YES	21	1			
Hypothetical protein	GL50803_3062	NO	53	1	NO	107	3				NO	165	3
DinF protein	GL50803_31295				NO	27	1						
Cathepsin L precursor	GL50803_3169				NO	64	2				NO	101	2
Hypothetical protein	GL50803_32419				NO	28	1				NO	37	1
Hypothetical protein	GL50803_32531										NO	30	1
Plasma membrane calcium-transporting ATPase 2	GL50803_32658	NO	367	8	NO	315	5	NO	51	2	NO	523	7
Hypothetical protein	GL50803_32681				NO	23	1				NO	30	1
Hypothetical protein	GL50803_32999	NO	26	1	NO	64	2	NO	87	3	YES	98	2
Hypothetical protein	GL50803_33513				NO	22	1						
Spindle pole protein, putative	GL50803_33660				NO	48	1						
NADH oxidase lateral transfer candidate	GL50803_33769	NO	780	12	NO	890	13	NO	299	5	NO	638	10
Hypothetical protein	GL50803_33809				NO	22	1				NO	27	1
Hypothetical protein	GL50803_33978				NO	22	1						
Coiled-coil protein	GL50803_3409				NO	23	1						
Hypothetical protein	GL50803_34094	NO	25	1	NO	27	1						
DEAD box RNA helicase Vasa	GL50803_34684				NO	31	1				NO	33	1
ABC transporter family protein	GL50803_3470	YES	43	1	YES	56	1				NO	95	2
Hypothetical protein	GL50803_3491	YES	25	1									
GTOR	GL50803_35180				NO	28	1				NO	27	1
Hypothetical protein	GL50803_35332										NO	21	1
Hypothetical protein	GL50803_35341										NO	22	1
Hypothetical protein	GL50803_3549										NO	22	1
Hypothetical protein	GL50803_3564	NO	41	1									
Hypothetical protein	GL50803_36426	NO	41	1							NO	193	4
DNA pol epsilon, sub B	GL50803_3706				NO	28	1				NO	28	1
Hypothetical protein	GL50803_37212				NO	22	1						
Hypothetical protein	GL50803_37350				NO	24	1						
Hypothetical protein	GL50803_3746										NO	22	1
Protein 21.1	GL50803_3762				NO	29	1						
Hypothetical protein	GL50803_37711										NO	34	1
Hypothetical protein	GL50803_38462										NO	26	1
Spindle pole protein, putative	GL50803_38517										NO	21	1
Hypothetical protein	GL50803_3867							NO	22	1			
Hypothetical protein	GL50803_38794				NO	23	1						
5' nucleotidase family protein	GL50803_3983				NO	65	1				NO	227	5
Polyadenylate-binding protein, putative	GL50803_3993				NO	23	1						
Hypothetical protein	GL50803_40006				NO	25	1						
Hypothetical protein	GL50803_40067										NO	100	1
Hypothetical protein	GL50803_4018				NO	24	1						
Hypothetical protein	GL50803_40228										NO	30	1
P24, putative	GL50803_40244				NO	30	1				YES	53	1
Alpha-19 giardin	GL50803_4026	NO	25	1							NO	26	1
Leucine-rich repeat protein	GL50803_4039	NO	560	6	NO	572	9	NO	162	3			

GiardiaDB r. 1.1			Experiment 1			Experiment 2			Experiment 3			Experiment 4	
Name	Accession number	MiD	Mascot score	Peptides	MiD	Mascot score	Peptides	MiD	Mascot score	Peptides	MiD	Mascot score	Peptides
Hypothetical protein	GL50803_4044	NO	26	1	NO	68	2	NO	43	1	NO	124	2
Dynein heavy chain	GL50803_40496				NO	24	1				NO	30	1
VSP	GL50803_40571							NO	22	1	NO	24	1
Actin related protein	GL50803_40817	NO	46	1	NO	24	1				NO	24	1
Hypothetical protein	GL50803_41212	NO	734	17	NO	985	21	NO	513	13			
Mucin-like protein	GL50803_41288	NO	25	1									
Protein 21.1	GL50803_41369				NO	25	1						
Hypothetical protein	GL50803_41451										NO	36	1
Kinase, CMGC CDK	GL50803_4191				NO	22	1						
High cysteine membrane protein Group 6	GL50803_41942										NO	89	3
Coiled-coil protein	GL50803_42000				NO	27	1						
Ciliary dynein heavy chain 11	GL50803_42285				YES	23	1						
Hypothetical protein	GL50803_42357				NO	48	2						
Protein 21.1	GL50803_4383				NO	22	1						
SALP-1	GL50803_4410				NO	953	15				NO	273	5
Hypothetical protein	GL50803_4622				NO	24	1						
Hypothetical protein	GL50803_4627										NO	30	1
Hypothetical protein	GL50803_4705				NO	48	1						
Hypothetical protein	GL50803_4768										YES	21	1
Beta-giardin	GL50803_4812				NO	1815	24				NO	1260	18
Hypothetical protein	GL50803_4816							NO	28	1	NO	99	1
Biglycan precursor	GL50803_5249				NO	26	1						
Hypothetical protein	GL50803_5258										NO	31	1
Hypothetical protein	GL50803_5274										NO	24	1
Kinase, NEK	GL50803_5375										YES	46	1
DUB-1	GL50803_5533										NO	26	1
Alpha-10 giardin	GL50803_5649	YES	294	5	NO	52	1	NO	139	3	NO	84	3
Sec61-alpha	GL50803_5744	NO	166	2	NO	163	2	YES	32	1	YES	175	3
Hypothetical protein	GL50803_5785	NO	103	1							NO	51	1
Leucine-rich repeat protein 1 virus receptor protein	GL50803_5795	NO	630	11	NO	626	14	NO	177	3	NO	1273	21
Hypothetical protein	GL50803_5809				NO	25	1						
Hypothetical protein	GL50803_5927	NO	31	1									
Protein 21.1	GL50803_6007				NO	33	1						
Protein 21.1	GL50803_6081				NO	30	1						
Hypothetical protein	GL50803_6082				NO	22	1	NO	20	1			
Kinase, NEK	GL50803_6140										NO	24	1
Alanyl dipeptidyl peptidase	GL50803_6148	NO	201	4	NO	182	4	NO	162	3			
Glycogen phosphorylase	GL50803_6226				NO	28	1				NO	58	1
Kinesin-3	GL50803_6262				NO	21	1						
High cysteine protein	GL50803_6372	NO	472	8	NO	531	8	NO	91	2	NO	877	12
14-3-3 protein	GL50803_6430	NO	78	2				YES	52	1			
Hypothetical protein	GL50803_6464	NO	111	2	NO	33	1						
Hypothetical protein	GL50803_6469				NO	38	1						
Metal-dependent hydrolase	GL50803_6497							YES	30	1			
Neurogenic locus notch-like protein	GL50803_6509										NO	175	2
Hypothetical protein	GL50803_6535				NO	22	1						
Hypothetical protein	GL50803_6617				YES	51	1				YES	350	5
Hypothetical protein	GL50803_6671										NO	44	1
Hypothetical protein	GL50803_6679	NO	34	1							NO	157	2
Glyceraldehyde 3-phosphate dehydrogenase	GL50803_6687	NO	81	2	NO	71	2						
Hypothetical protein	GL50803_6725				NO	26	1				NO	26	1
Zinc finger domain	GL50803_6733	YES	55	1									
Spindle pole protein, putative	GL50803_7031	NO	68	1	NO	47	2	NO	68	2			
Ubiquitin	GL50803_7110	NO	202	3	NO	283	5	YES	121	2	NO	360	5
Hypothetical protein	GL50803_7130				NO	41	1						
Kinase, NEK-frag	GL50803_7183							YES	22	1			
Hypothetical protein	GL50803_7188	YES	224	4	NO	473	8	YES	78	1	NO	926	11
Hypothetical protein	GL50803_7242	YES	69	1	YES	47	1				NO	47	1
Hypothetical protein	GL50803_7244				NO	79	2				YES	144	3
CDP-diacylglycerol-glycerol-3-phosphate 3-phosphatidyltrar	GL50803_7259										NO	43	1
Protein 21.1	GL50803_7268							NO	69	1			
Syntaxin-like protein 1	GL50803_7309				NO	81	1	NO	22	1			
Protein 21.1	GL50803_7373										NO	22	1
Vacuolar ATP synthase catalytic subunit A	GL50803_7532	YES	146	2									
Lysosomal acid phosphatase precursor	GL50803_7556	NO	130	3	NO	286	5	NO	87	2			
SMC6 protein	GL50803_7648										NO	27	1

GiardiaDB r. 1.1			Experiment 1			Experiment 2			Experiment 3			Experiment 4	
Name	Accession number	MiD	Mascot score	Peptides	MiD	Mascot score	Peptides	MiD	Mascot score	Peptides	MiD	Mascot score	Peptides
Kinase, NEK-like	GL50803_7653										NO	22	1
Hypothetical protein	GL50803_7661				NO	21	1						
Protein 21.1	GL50803_7680	NO	26	1									
High cysteine membrane protein Group 1	GL50803_7715	NO	89	2	NO	41	1				NO	76	1
ADP-ribosylation factor	GL50803_7789	NO	79	1									
Alpha-2 giardin	GL50803_7796	NO	427	5	NO	478	8	YES	212	5			
Coiled-coil protein	GL50803_7829										NO	54	2
Kinesin-16	GL50803_7874				NO	23	1						
Ribosomal protein S14	GL50803_7878				NO	24	1						
Amino acid transporter family	GL50803_7909	NO	159	2	NO	66	2				NO	123	2
Cleavage and polyadenylation specificity factor, 73 kDa sub	GL50803_8026										NO	23	1
Seven transmembrane protein 1	GL50803_8044	NO	135	2	NO	176	3	NO	145	2	NO	220	3
Protein disulfide isomerase PDI5	GL50803_8064	YES	28	1	YES	58	1	NO	33	1			
Kinase, NEK	GL50803_8152	NO	28	1									
Hypothetical protein	GL50803_8160	NO	37	1									
Manganese-dependent inorganic pyrophosphatase, putative	GL50803_8163				YES	25	1						
Phospholipid-transporting ATPase IA, putative	GL50803_8182							NO	44	1			
ATP-binding cassette protein 5	GL50803_8227	NO	25	1	NO	25	1				NO	121	2
Hypothetical protein	GL50803_8243										NO	26	1
Kinase, NEK	GL50803_8350										NO	25	1
Acid sphingomyelinase-like phosphodiesterase 3b precurso	GL50803_8360				NO	66	2						
MDR-type permease	GL50803_8444	NO	65	1	NO	50	2	NO	178	4	NO	95	3
Ribosomal protein L27	GL50803_8462				NO	28	1				NO	43	1
Gp49, putative	GL50803_8471										NO	123	1
Rac	GL50803_8496	NO	48	1									
Hypothetical protein	GL50803_8505	NO	187	2	NO	49	1				NO	85	2
Kinase, NEK	GL50803_8556				NO	34	1						
Vacuolar ATP synthase 16 kDa proteolipid subunit	GL50803_8559	YES	90	1	NO	34	1				NO	77	1
Coiled-coil protein	GL50803_8564	YES	74	3							NO	22	1
Kinase, AGC NDR	GL50803_8587										YES	22	1
Suppressor of actin 1	GL50803_8589	NO	72	1	YES	81	2	NO	66	2	NO	51	1
Hypothetical protein	GL50803_86468				NO	220	3	NO	172	2	NO	603	8
Acyl-CoA synthetase	GL50803_86511	NO	176	2									
Delta giardin	GL50803_86676				NO	707	12				NO	263	5
Protein 21.1	GL50803_86821	NO	23	1									
Protein 21.1	GL50803_86855										YES	22	1
Tenascin precursor	GL50803_8687				NO	81	2						
Putative serine	GL50803_8733				NO	24	1						
Dipeptidyl-peptidase I precursor	GL50803_8741	NO	211	4	NO	475	9	NO	83	2	NO	709	12
ABC transporter family protein	GL50803_87446	YES	92	3	NO	191	4				NO	554	7
Hypothetical protein	GL50803_87519	NO	240	3									
Tubby superfamily protein	GL50803_87817	NO	23	1	NO	29	1						
Kinase, SCY1	GL50803_8805	YES	83	1	NO	159	2				YES	156	2
Spindle pole protein, putative	GL50803_8820				NO	21	1						
Protein 21.1	GL50803_88245										YES	23	1
ATPase	GL50803_88438	NO	29	1	NO	23	1						
Cytosolic HSP70	GL50803_88765							YES	22	1			
Hypothetical protein	GL50803_17161				YES	208	2						
Copine I	GL50803_8903	NO	121	2	NO	190	4	NO	75	1	YES	87	2
MCM5	GL50803_89112				NO	23	1						
Mu adaptin	GL50803_8917				NO	24	1						
Hypothetical protein	GL50803_8955				NO	23	1						
Mu adaptin	GL50803_89622							NO	23	1			
Hypothetical protein	GL50803_89828	NO	23	1	NO	31	1						
Hypothetical protein	GL50803_9036										NO	39	1
Hypothetical protein	GL50803_90425										NO	23	1
Hypothetical protein	GL50803_9045	NO	69	1	NO	60	1						
Long chain fatty acid CoA ligase 5	GL50803_9062	NO	173	3	YES	279	3	NO	25	1	YES	157	2
Hypothetical protein	GL50803_90710				NO	33	1						
ERP2	GL50803_9079										NO	53	1
High cysteine membrane protein Group 2	GL50803_91099				YES	47	1				YES	65	1
Nitric oxide synthase, inducible	GL50803_91252				NO	40	1						
Hypothetical protein	GL50803_9143										NO	22	1
Hypothetical protein	GL50803_91611	NO	24	1	NO	33	1						
Nuclear LIM interactor-interacting factor 1	GL50803_9162										NO	24	1
Amino acid transporter family	GL50803_91712	NO	76	2	NO	31	1				NO	40	1

GiardiaDB r. 1.1		Experiment 1			Experiment 2			Experiment 3			Experiment 4		
Name	Accession number	MiD	Mascot score	Peptides	MiD	Mascot score	Peptides	MiD	Mascot score	Peptides	MiD	Mascot score	Peptides
Hypothetical protein	GL50803_92132	NO	39	1	NO	21	1						
ABC transporter family protein	GL50803_92223	NO	85	2	NO	63	1	NO	80	2	NO	260	4
Cation-transporting ATPase 2, putative	GL50803_92246				NO	26	1						
Hypothetical protein	GL50803_9259										NO	24	1
5' nucleotidase family protein	GL50803_92645	NO	162	3	NO	171	3	NO	112	1	NO	429	7
Kinase, CMGC CLK	GL50803_92741	NO	26	1	NO	27	1				NO	24	1
Protein phosphatase 2C-like protein	GL50803_9293	NO	22	1							NO	23	1
Hypothetical protein	GL50803_9296	YES	31	1	YES	178	4				YES	35	1
Alcohol dehydrogenase	GL50803_93358	NO	716	10	NO	293	7	NO	76	2			
Phospholipase B	GL50803_93548	NO	487	9	NO	597	9	NO	372	6	NO	870	12
Casein kinase II beta chain	GL50803_93671										NO	25	1
Dynein heavy chain	GL50803_93736										YES	29	1
Protein disulfide isomerase PDI2	GL50803_9413	NO	91	2	NO	570	9	NO	79	2	NO	659	10
Hypothetical protein	GL50803_94287				NO	23	1						
Spastin	GL50803_94322	YES	23	1	NO	42	1				NO	66	2
ABC-type transport system ATP-binding chain, putative	GL50803_94478										NO	93	3
Hypothetical protein	GL50803_94542	NO	29	1									
Hypothetical protein	GL50803_94658										YES	27	1
Alpha mitochondrial protein peptidase like protein	GL50803_9478	YES	30	1									
Hypothetical protein	GL50803_9485				NO	63	2						
Hypothetical protein	GL50803_9503	NO	35	1	NO	92	2				YES	206	3
Metalloprotease, insulinase family	GL50803_9508	NO	22	1	NO	23	1				NO	23	1
Coiled-coil protein	GL50803_9515				YES	61	2						
Tenascin-like	GL50803_95162	NO	795	9	NO	660	11				NO	266	5
Hypothetical protein	GL50803_95406				NO	26	1						
Cathepsin L precursor	GL50803_9548										NO	38	1
Rab1a	GL50803_9558	NO	62	1				NO	42	1			
Hypothetical protein	GL50803_9573				NO	30	1						
Hypothetical protein	GL50803_95908										NO	24	1
E3 ubiquitin ligase for Rad6p required for the ubiquitination	GL50803_95918										NO	24	1
Spindle pole protein, putative	GL50803_96018	NO	29	1									
Hypothetical protein	GL50803_9611										NO	22	1
High cysteine membrane protein Group 2	GL50803_9620				NO	91	2				NO	175	3
Hypothetical protein	GL50803_9659				NO	23	1	NO	21	1			
Potassium-transporting ATPase alpha chain 1	GL50803_96670	YES	354	8	NO	270	5	NO	162	3	NO	473	9
Hypothetical protein	GL50803_96818	NO	60	2	NO	54	1				NO	99	2
Hypothetical protein	GL50803_9697				NO	23	1						
Hypothetical protein	GL50803_97017				NO	23	1						
Kinase, NEK	GL50803_97130				NO	23	1						
NADH oxidase	GL50803_9719	NO	271	5	YES	222	5	YES	31	1	YES	166	3
Protein 21.1	GL50803_9720				NO	27	1						
ABC transporter, ATP-binding protein	GL50803_9741				NO	21	1				NO	22	1
Chaperone protein dnaJ	GL50803_9751				NO	34	1				YES	28	1
Hypothetical protein	GL50803_9780				NO	252	5	NO	48	1	YES	333	5
Dynein light chain	GL50803_9848	NO	49	1							NO	105	2
Hypothetical protein	GL50803_9861										YES	92	2
Pyruvate, phosphate dikinase	GL50803_9909										NO	57	1
PAM18	AACB02000023-5-32114-31794										YES	68	1
