# Supplementary material for: The Minimal Proteome in the Reduced Mitochondrion of the Parasitic Protist Giardia intestinalis
Source: PLoS One. 2011 Feb 24;6(2):e17285. doi: 10.1371/journal.pone.0017285 (PMC3044749; doi:10.1371/journal.pone.0017285)
Supplement: Table S2 — List of Giardia proteins within the mitosomal distribution range (MiD) identified by LC MS/MS. (PDF) [file pone.0017285.s011.pdf]

Table S2. List of *Giardia* proteins within the mitosomal distribution range (MiD) identified by LC MS/MS.

GiardiaDB r1.1		PSI BLAST				PFAM				
Annotation	Accession No.	ACCESSION	DEFINITION	Score	E-Value	start	end	ID No.	E-value	Protein families
PAM18	XP_002364144	ref:XP_001581820.1	DnaJ domain containing protein [Trichomonas vaginalis G3]	54	0.000005					
Hypothetical protein	GL50803_10016					154	230	PF00085.13	0.0011	Thioredoxin
Kinesin-3	GL50803_102101	gb:EFA85017.1	kinesin-3 [Polysphondylium pallidum PN500]	400	e-109	591	829	PB004124	2.6E-73	Pfam-B_4124
Ornithine carbamoyltransferase	GL50803_10311	ref:XP_001315726.1	ornithine carbamoyltransferase family protein [Trichomonas vaginalis G3]	328	6E-88	159	319	PF00185.17	2.3E-46	OTCace
Tenascin precursor	GL50803_10330	gb:EDL26780.1	mCG134590, isoform CRA_c [Mus musculus]	81	1E-13	24	67	PF01826.10	0.00017	TIL
High cysteine membrane protein Group 1	GL50803_103454	gb:AAW83026.1	proprotein convertase 6C [Xenopus lae...	57	0.000008	334	717	PF03302.6	3.2E-38	VSP
Chaperonin 60	GL50803_103891	ref:ZP_01548078.1	chaperonin GroEL [Stappia aggregata IAM 12614]	365	5E-99	25	535	PF00118.17	1.9E-101	Cpn60_TCP1
Hypothetical protein	GL50803_111809									
Alpha-tubulin	GL50803_112079	gb:AAN78301.1	alpha-tubulin [Encephalitozoon intestinalis]	872	0	263	392	PF03953.10	2.4E-51	Tubulin_C
Elongation factor 1-alpha	GL50803_112312	pir:S70634	translation elongation factor eEF-1 alpha chain - Hexamita sp. (strain ATC(	719	0	5	216	PF00009.20	6.6E-56	GTP_EFTU
Hypothetical protein	GL50803_11237					230	427	PB011818	3.7E-49	Pfam-B_11818
Ribosomal protein S4	GL50803_11359	dbj:BAE20168.1	ribosomal protein S4 [Panax ginseng]	314	6E-84	95	169	PF00900.13	3.4E-29	Ribosomal_S4e
Cysteine protease	GL50803_113656	ref:XP_001741082.1	cysteine proteinase ACP1 precursor [Entamoeba dispar SAW760]	76	2E-11	449	518	PF00112.16	2E-19	Peptidase_C1
Long chain fatty acid CoA ligase, putative	GL50803_113892	ref:XP_002449702.1	hypothetical protein SORBIDRAFT_05g021840 [Sorghum bicolor]	214	3E-53	79	642	PF00501.21	1.5E-80	AMP-binding
High cysteine membrane protein Group 4	GL50803_114042	ref:XP_001021017.1	hypothetical protein TTHERM_00305400 [Tetrahymena thermophila]	73	2E-10	778	810	PF03302.6	0.00000022	VSP
VSP with INR	GL50803_11470	ref:XP_001021653.1	hypothetical protein TTHERM_00151170 [Tetrahymena thermophila]	102	2E-19	532	673	PF03302.6	2.1E-50	VSP
Hypothetical protein	GL50803_114777	gb:ABI15615.1	hypothetical protein [Spironucleus barkhanus]	251	3E-64	2	192	PB000845	1.4E-33	Pfam-B_845
VSP	GL50803_11521	ref:XP_214004.4	PREDICTED: similar to Fras1 protein [Rattus norvegicus]	108	3E-21	487	628	PF03302.6	1.7E-52	VSP
Hypothetical protein	GL50803_11557					61	123	PB011995	0,00	Pfam-B_11995
Alpha-1 giardin	GL50803_11654	gb:ACQ58091.1	Annexin A5 [Anoplopoma fimbria]	57	0.000002	8	67	PF00191.13	7.1E-09	Annexin
Kinase, NEK-frag	GL50803_11775	ref:XP_001198470.1	PREDICTED: similar to ankyrin 2,3/unc44 [Strongylocentrotus purpuratus]	91	2E-15	169	282	PF00069.18	0.0001	Pkinase
Hypothetical protein	GL50803_11866					78	130	PF00583.17	0.000089	Acetyltransf_1
Coatomer alpha subunit	GL50803_11953	ref:XP_660630.1	hypothetical protein AN3026.2 [Aspergillus nidulans FGSC A4]	342	2E-91	47	80	PF00400.25	1.8E-11	WD40
Elongation factor 1-gamma	GL50803_12102	ref:XP_002610271.1	hypothetical protein BRAFLDRAFT_115434 [Branchiostoma floridae]	110	3E-22	235	340	PF00647.12	3.8E-30	EF1G
Hypothetical protein	GL50803_12999									
Vacuolar ATP synthase subunit d	GL50803_13000	ref:NP_189513.1	H+-transporting two-sector ATPase, putative [Arabidopsis thaliana]	167	2E-39	17	346	PF01992.9	1.6E-32	vATP-synt_AC39
Hypothetical protein	GL50803_13288	gb:AAI24607.1	Zgc:152917 [Danio rerio]	56	0.00001					
Hypothetical protein	GL50803_13413									
Translation elongation factor	GL50803_13561	ref:XP_002407515.1	elongation factor 1-beta [Ixodes scapularis]	140	1E-31	131	219	PF00736.12	2.5E-28	EF1_GNE
Beta tubulin	GL50803_136020	gb:AAI53757.1	LOC100037222 protein [Xenopus laevis]	921	0	3	222	PF00091.18	7.4E-72	Tubulin
VSP	GL50803_137618	ref:XP_001028021.1	Protein kinase domain containing protein [Tetrahymena thermophila]	116	1E-23	455	853	PF03302.6	1.2E-130	VSP
Hypothetical protein	GL50803_137685	ref:XP_002055439.1	GJ18789 [Drosophila virilis]	471	e-130	15	674	PF02516.7	9.1E-80	STT3
Hypothetical protein	GL50803_137746									
Hypothetical protein	GL50803_13922					1	334	PB001790	2.4E-33	Pfam-B_1790
Hypothetical protein	GL50803_14164	ref:XP_654729.1	molybdenum cofactor sulfurase [Entamoeba histolytica HM-1:IMSS]	401	e-109					
Molybdenum cofactor sulfurase	GL50803_14200	ref:XP_001907166.1	unnamed protein product [Podospora anserina]	113	3E-23	352	598	PF00266.12	1.8E-20	Aminotran_5
Kinase, NEK	GL50803_14223	ref:XP_001907166.1	unnamed protein product [Podospora anserina]	113	3E-23	19	271	PF00069.18	1.6E-36	Pkinase
CXC-rich protein	GL50803_14225	gb:EFC39035.1	predicted protein [Naegleria gruberi]	79	6E-12	53	246	PB001790	0.0000034	Pfam-B_1790
Hypothetical protein	GL50803_14278									
Synaptobrevin-like protein	GL50803_14469	ref:XP_002273961.1	PREDICTED: hypothetical protein [Vitis vinifera]	55	0.000009	150	234	PF00957.14	2E-17	Synaptobrevin
Cysteine desulfurase	GL50803_14519	emb:CAL52881.1	Cysteine desulfurase NFS1 (ISS) [Ostreococcus tauri]	417	e-114	2	394	PF00266.12	1.3E-71	Aminotran_5
Chaperone protein DnaK HSP70	GL50803_14581	ref:YP_001491913.1	molecular chaperone DnaK [Rickettsia canadensis str. McKiel]	464	e-128	9	602	PF00012.13	2.1E-162	HSP70
Hypothetical protein	GL50803_14660	ref:XP_002498711.1	ZYRO0G16786p [Zygosaccharomyces rouxii]	50	0.00006	18	116	PF03179.8	1.6E-12	V-ATPase_G
Protein disulfide isomerase PDI3	GL50803_14670	ref:XP_651032.1	thioredoxin [Entamoeba histolytica HM-1:IMSS]	101	3E-20	19	114	PF00085.13	0,00	Thioredoxin
HesB domain-containing protein	GL50803_14821	ref:NP_001102748.1	hypothetical protein LOC500694 [Rattus norvegicus]	100	6E-20	18	103	PF01521.13	2.2E-17	Fe-S_biosyn
Hypothetical protein	GL50803_14845									
Hypothetical protein	GL50803_14939									
Pyrophosphate-fructose 6-phosphate 1-pho	GL50803_14993	gb:AAL16943.1	putative pyrophosphate-dependent phosphofructokinase [Hexamita inflata]	778	0	69	315	PF00365.13	3.6E-21	PFK
Hypothetical protein	GL50803_15084									
Alpha-14 giardin	GL50803_15097	ref:NP_001079765.1	hypothetical protein LOC379455 [Xenopus laevis]	93	5E-17	88	143	PF00191.13	0.000000052	Annexin
NifU-like protein	GL50803_15196	ref:ZP_02901680.1	FeS cluster assembly scaffold IscU [Escherichia albertii TW07627]	184	5E-45	80	203	PF01592.9	4.5E-45	NifU_N
ERP3	GL50803_15204	ref:XP_002492913.1	Protein with similarity to Emp24p and Erv25p, member of the p24 family [P	55	0.000005	17	201	PF01105.17	3.6E-25	EMP24_GP25L
Peroxiredoxin 1	GL50803_16076	gb:AAP33385.1	thioredoxin peroxidase [Spironucleus barkhanus]	273	6E-72	161	196	PF10417.2	0.000000045	1-cysPrx_C
Coiled-coil protein	GL50803_16152					263	535	PF10174.2	0.000097	Cast
Hypothetical protein	GL50803_16313	emb:CAD98642.1	conserved BRCA1 domain protein [Cryptosporidium parvum]	296	4E-78	7	249	PF06732.4	4.7E-81	Pescadillo_N
Protein 21.1	GL50803_16354	ref:XP_001829840.1	hypothetical protein CC1G_06049 [Coprinopsis cinerea okayama7#130]	64	0.00000005	651	672	PF00023.23	0.000025	Ank
Hypothetical protein	GL50803_16424	ref:XP_002006769.1	GI21251 [Drosophila mojavensis]	50	0.0003	45	169	PF10248.2	0.00031	Mlf1IP
Hypothetical protein	GL50803_16430									
Tenascin-37	GL50803_16477	gb:EFC46548.1	hypothetical protein NAEGRDRAFT_79129 [Naegleria gruberi]	117	3E-24	352	377	PF07974.6	0.000018	EGF_2
Kinase, NEK	GL50803_16824	ref:XP_001180091.1	PREDICTED: similar to ankyrin 2,3/unc44 [Strongylocentrotus purpuratus]	148	2E-33	17	288	PF00069.18	1.4E-39	Pkinase
Tenascin-like	GL50803_16833	gb:EDL26779.1	mCG134590, isoform CRA_b [Mus musculus]	98	3E-18	579	603	PF07974.6	0.000039	EGF_2
Phosphatidate cytidylyltransferase	GL50803_16906	ref:XP_001631675.1	predicted protein [Nematostella vectensis]	122	9E-26	29	373	PF01148.13	1.1E-38	CTP_transf_1
FtsJ cell division protein, putative	GL50803_16993	ref:XP_001259487.1	rRNA methyltransferase Spb1, putative [Neosartorya fischeri NRRL 181]	323	5E-86	854	1054	PF07780.5	4.8E-55	Spb1_C
Hypothetical protein	GL50803_16998	gb:EEE19788.1	conserved hypothetical protein [Toxoplasma gondii GT1]	112	1E-22					
Bip	GL50803_17121	sp:Q03684.1	RecName: Full=Luminal-binding protein 4; Short=BiP 4; AltName: Full=78	771	0	30	652	PF00012.13	6.2E-232	HSP70
Hypothetical protein	GL50803_17161					13	242	PF01459.15	0.0000018	Porin_3
ABC transporter, ATP-binding protein	GL50803_17165	ref:XP_001023593.1	ABC transporter family protein [Tetrahymena thermophila]	147	6E-33	623	733	PF00005.20	0.00000045	ABC_tran

GiardiaDB r1.1		PSI BLAST				PFAM				
**Annotation**	**Accession No.**	**ACCESSION**	**DEFINITION**	**Score**	**E-Value**	**start**	**end**	**ID No.**	**E-value**	**Protein families**
Hypothetical protein	GL50803_17236					26	206	PF07786.5	0.027	DUF1624
Protein 21.1	GL50803_17288	gb:EDL26002.1	uveal autoantigen with coiled-coil d...	53	0.0003	1596	1618	PF00023.23	0.00048	Ank
Hypothetical protein	GL50803_17296	ref:XP_001014709.1	major facilitator superfamily protein [Tetrahymena thermophila]	64	0.00000007	84	466	PF07690.9	0,00	MFS_1
High cysteine membrane protein Group 2	GL50803_17328	ref:XP_976535.1	hypothetical protein TTHERM_01178660 [Tetrahymena thermophila]	404	e-110	807	1127	PF03302.6	2.5E-10	VSP
Hypothetical protein	GL50803_17342	gb:ABI15615.1	hypothetical protein [Spironucleus barkhanus]	256	5E-66	2	192	PB000845	8.1E-34	Pfam-B_845
TCP-1 chaperonin subunit gamma	GL50803_17411	gb:AAL35373.1	CCT chaperonin gamma subunit [Physarum polycephalum]	565	e-159	31	558	PF00118.17	1.7E-142	Cpn60_TCP1
CXC-rich protein	GL50803_17476	ref:XP_651577.2	tyrosine kinase [Entamoeba histolytica HM-1:IMSS]	150	3E-33	955	1264	PB001790	8.1E-30	Pfam-B_1790
Kinase, NEK	GL50803_17510	ref:XP_001649474.1	ankyrin 2,3/unc44 [Aedes aegypt...	96	1E-17	30	282	PF00069.18	4.6E-28	Pkinase
Vacuolar proton-ATPase subunit, putative	GL50803_18470	ref:XP_570271.1	vacuolar (H+)-ATPase subunit [Cryptococcus neoformans var. neoformans	424	e-116	425	919	PF01496.12	7.2E-139	V_ATPase_I
Hypothetical protein	GL50803_19230					42	115	PF03656.6	0.0063	Pam16
Hypothetical protein	GL50803_1937					2	93	PB004452	6.7E-20	Pfam-B_4452
Glutaredoxin-related protein	GL50803_2013	ref:ZP_06171702.1	glutaredoxin-like protein [Brevundimonas subvibrioides ATCC 15264]	126	2E-27	115	179	PF00462.17	8.1E-16	Glutaredoxin
Long chain fatty acid CoA ligase 5	GL50803_21118	ref:XP_001761279.1	predicted protein [Physcomitrella patens subsp. patens]	206	7E-51	95	649	PF00501.21	4.5E-80	AMP-binding
ABC transporter, ATP-binding protein	GL50803_21411	ref:XP_001032330.2	ABC transporter family protein [Tetrahymena thermophila]	146	2E-32	5	271	PB002445	2.6E-18	Pfam-B_2445
Spindle pole protein, putative	GL50803_21444									
Hypothetical protein	GL50803_23389	gb:EEE27081.1	cAMP-specific phosphodiesterase, putative [Toxoplasma gondii GT1]	53	0.0002	549	731	PB015975	1.6E-64	Pfam-B_15975
Protein 21.1	GL50803_23492	ref:XP_001200736.1	PREDICTED: similar to ankyrin 2,3/unc44, partial [Strongylocentrotus purp	192	1E-46	658	681	PF00023.23	0.000031	Ank
Vacuolar protein sorting 35	GL50803_23833	ref:XP_001179974.1	PREDICTED: similar to vacuolar protein sorting 35, partial [Strongylocentrc	115	2E-23	169	349	PF03635.10	6.1E-24	Vps35
[2Fe-2S] ferredoxin	GL50803_27266	ref:ZP_01864211.1	Ferredoxin, 2Fe-2S [Erythrobacter sp. SD-21]	80	6E-14	17	97	PF00111.20	1.6E-10	Fer2
Multidrug resistance-associated protein 1	GL50803_28379	gb:ABI15579.1	ATP-binding cassette subfamily C (CFTR/MRP) member 5 [Spironucleus b	652	0	900	1146	PF00664.16	4E-18	ABC_membrane
Hypothetical protein	GL50803_28962					141	222	PF02480.9	0.0000018	Herpes_gE
Hypothetical protein	GL50803_29327	ref:XP_001746337.1	hypothetical protein [Monosiga brevicollis MX1]	77	1E-12	18	170	PF03357.14	8.4E-15	Snf7
Hypothetical protein	GL50803_29500					33	82	PF00166.14	0.018	Cpn10
Hypothetical protein	GL50803_3021									
Hypothetical protein	GL50803_32999	gb:EFA79563.1	zipper-like domain-containing protein [Polysphondylium pallidum PN500]	70	4E-10	91	173	PB000039	0.000054	Pfam-B_39
ABC transporter family protein	GL50803_3470	ref:XP_001016586.1	ABC transporter family protein [Tetrahymena thermophila]	249	1E-63	801	922	PF00005.20	3.9E-17	ABC_tran
Hypothetical protein	GL50803_3491									
P24, putative	GL50803_40244					8	94	PF01105.17	0.000000036	EMP24_GP25L
Ciliary dynein heavy chain 11	GL50803_42285	ref:XP_001634257.1	predicted protein [Nematostella vectensis]	191	3E-45	2594	2895	PF08393.6	3.9E-38	DHC_N2
Hypothetical protein	GL50803_4768									
Kinase, NEK	GL50803_5375	dbj:BAA77339.1	Nek2A [Xenopus laevis]	285	7E-75	37	299	PF00069.18	4.7E-68	Pkinase
Alpha-10 giardin	GL50803_5649	pdb:1BC1	Chain A, Recombinant Rat Annexin V, Quadruple Mutant (T72k, S144k, S2	59	0.0000008	51	99	PF00191.13	0.00033	Annexin
Sec61-alpha	GL50803_5744	ref:XP_002285514.1	PREDICTED: hypothetical protein [Vitis vinifera]	417	e-114	93	479	PF00344.13	1.5E-68	SecY
14-3-3 protein	GL50803_6430	gb:EFC43173.1	predicted protein [Naegleria gruberi]	323	8E-87	7	241	PF00244.13	2.1E-104	14.3.2003
Metal-dependent hydrolase	GL50803_6497	ref:ZP_02962021.2	hypothetical protein PROSTU_04109 [Providencia stuartii ATCC 25827]	99	7E-19	27	79	PF00753.20	0.00000066	Lactamase_B
Hypothetical protein	GL50803_6617									
Zinc finger domain	GL50803_6733	ref:XP_002007529.1	GI12340 [Drosophila mojavensis]	112	8E-23	129	177	PF01529.13	2.5E-20	zf-DHHC
Ubiquitin	GL50803_7110	emb:CAI83757.1	Polyubiquitin 4 [Polyplastron multivesiculatum]	144	2E-33	11	79	PF00240.16	3.5E-34	ubiquitin
Kinase, NEK-frag	GL50803_7183	ref:XP_001580653.1	ankyrin repeat protein [Trichomonas vaginalis G3]	103	9E-20	59	266	PF00069.18	1.7E-16	Pkinase
Hypothetical protein	GL50803_7188	ref:YP_656868.1	halomucin [Haloquadratum walsbyi DSM 16790]	55	0.00006	6	329	PB001790	6.5E-31	Pfam-B_1790
Vacuolar ATP synthase catalytic subunit A	GL50803_7532	sp:P48414.1	RecName: Full=V-type proton ATPase catalytic subunit A; Short=V-ATPas	627	e-177	234	501	PF00006.18	2.2E-96	ATP-synt_ab
Alpha-2 giardin	GL50803_7796	gb:AAL25093.1	annexin [Artemia franciscana]	57	0.000002	18	67	PF00191.13	0.000000052	Annexin
Protein disulfide isomerase PDI5	GL50803_8064	gb:EEH57167.1	predicted protein [Micromonas pusilla CCMP1545]	115	2E-24	37	132	PF00085.13	3.1E-31	Thioredoxin
Manganese-dependent inorganic pyrophos	GL50803_8163	ref:YP_900441.1	putative manganese-dependent inorganic pyrophosphatase [Pelobacter pro	176	1E-41	37	515	PF01368.13	5.6E-22	DHH
Vacuolar ATP synthase 16 kDa proteolipid	GL50803_8559	gb:EEY61709.1	V-type proton ATPase 16 kDa proteolipid subunit, putative [Phytophthora ir	174	4E-42	97	162	PF00137.14	1.5E-20	ATP-synt_C
Coiled-coil protein	GL50803_8564					642	699	PF10211.2	0.0034	Ax_dynein_light
Kinase, AGC NDR	GL50803_8587	emb:CAN61876.1	hypothetical protein [Vitis vinifera]	322	6E-86	74	237	PF00069.18	8.9E-33	Pkinase
Suppressor of actin 1	GL50803_8589	ref:XP_964812.1	hypothetical protein NCU00896 [Neurospora crassa OR74A]	120	4E-25	53	336	PF02383.11	6.2E-44	Syja_N
Protein 21.1	GL50803_86855	ref:ZP_01618981.1	hypothetical protein L8106_01562...	62	0.0000004	899	949	PF04102.5	0.00059	SlyX
ABC transporter family protein	GL50803_87446	ref:XP_001846619.1	ATP-binding cassette sub-family A member 3 [Culex quinquefasciatus]	153	1E-34	664	769	PF00005.20	7E-16	ABC_tran
Kinase, SCY1	GL50803_8805	ref:XP_797681.1	PREDICTED: similar to SCY1-like 2 protein, partial [Strongylocentrotus pur	83	2E-13	756	834	PB000079	0.0000048	Pfam-B_79
Protein 21.1	GL50803_88245	ref:XP_001329422.1	ankyrin repeat protein [Trichomonas vaginalis G3]	308	4E-81	382	402	PF00023.23	0.00017	Ank
Cytosolic HSP70	GL50803_88765	gb:AAN08152.1	cytosolic heat shock protein 70 [Spironucleus barkhanus]	890	0	6	615	PF00012.13	3.7E-249	HSP70
Copine I	GL50803_8903	gb:EFA85793.1	hypothetical protein PPL_01023 [Polysphondylium pallidum PN500]	215	4E-54	50	189	PF07002.9	1.2E-32	Copine
Long chain fatty acid CoA ligase 5	GL50803_9062	gb:ABA96461.2	AMP-binding enzyme family protein, expressed [Oryza sativa (japonica cul	312	1E-82	360	688	PF00501.21	1.7E-65	AMP-binding
High cysteine membrane protein Group 2	GL50803_91099	gb:AAH71562.2	NOTCH2 protein [Homo sapiens]	83	3E-13	538	561	PF00008.20	0.00085	EGF
Hypothetical protein	GL50803_9296									
Dynein heavy chain	GL50803_93736	ref:XP_828326.1	dynein heavy chain [Trypanosoma brucei TREU927]	452	e-124	201	621	PB001786	1.9E-116	Pfam-B_1786
Spastin	GL50803_94322	ref:XP_002572778.1	hypothetical protein [Schistosoma mansoni]	123	1E-25	784	857	PF00004.22	3.7E-10	AAA
Hypothetical protein	GL50803_94658	ref:XP_002186415.1	predicted protein [Phaeodactylum tricornutum CCAP 1055/1]	74	6E-11	252	515	PF05021.8	8.5E-13	NPL4
Alpha mitochondrial protein peptidase like p	GL50803_9478	ref:ZP_05534614.1	protease [Streptomyces viridochromogenes DSM 40736]	60	0.0000004	13	149	PF00675.13	7.6E-10	Peptidase_M16
Hypothetical protein	GL50803_9503	ref:YP_002008055.1	putative hydrolase or acyltransferase [Cupriavidus taiwanensis]	63	0.00000003	71	303	PF00561.13	1E-11	Abhydrolase_1
Coiled-coil protein	GL50803_9515	ref:XP_001309423.1	hypothetical protein [Trichomonas vaginalis G3]	68	0.000000008					
Potassium-transporting ATPase alpha chai	GL50803_96670	ref:NP_001117930.1	Na/K ATPase alpha subunit isoform 2 [Oncorhynchus mykiss]	661	0	222	454	PF00122.13	6.4E-58	E1-E2_ATPase
NADH oxidase	GL50803_9719	ref:YP_001713634.1	putative oxidoreductase, FAD/FMN-binding [Acinetobacter baumannii AYE	132	6E-29	14	215	PF00724.13	0.00000015	Oxidored_FMN
Chaperone protein dnaJ	GL50803_9751	ref:XP_952078.1	hypothetical protein [Theileria annulata strain Ankara]	67	0.000000002	264	317	PF00226.24	1.7E-15	DnaJ
Hypothetical protein	GL50803_9780					9	89	PF08400.3	0.022	phage_tail_N
Hypothetical protein	GL50803_9861									
