# Supplementary material for: The Minimal Proteome in the Reduced Mitochondrion of the Parasitic Protist Giardia intestinalis
Source: PLoS One. 2011 Feb 24;6(2):e17285. doi: 10.1371/journal.pone.0017285 (PMC3044749; doi:10.1371/journal.pone.0017285)
Supplement: Table S3 — Identification of protein families using PfamA+B databases. (PDF) [file pone.0017285.s012.pdf]

Table S3. Identification of protein families using PfamA+B databases.

GiardiaDB r1.1	PFAM A+B identification (cut off 0.05)				
accession No.	start	end	ID No.	E-value	Protein families
GL50803_10016	38	125	PF09794.2	2.20E-02	Avl9
GL50803_10016	154	230	PF00085.13	1.10E-03	Thioredoxin
GL50803_102101	11	351	PF00225.16	0,00	Kinesin
GL50803_102101	591	829	PB004124	2.60E-73	Pfam-B_4124
GL50803_103891	25	535	PF00118.17	1.90E-101	Cpn60_TCP1
GL50803_113656	168	208	PF00112.16	3.90E-07	Peptidase_C1
GL50803_113656	449	518	PF00112.16	2.00E-19	Peptidase_C1
GL50803_113892	79	642	PF00501.21	1.50E-80	AMP-binding
GL50803_113892	660	745	PB001299	2.40E-04	Pfam-B_1299
GL50803_114042	405	607	PF03302.6	0,00	VSP
GL50803_114042	778	810	PF03302.6	2.20E-07	VSP
GL50803_114042	778	805	PF08693.3	1.80E-02	SKG6
GL50803_114777	2	192	PB000845	1.40E-33	Pfam-B_845
GL50803_114777	215	537	PF07690.9	5.90E-25	MFS_1
GL50803_11775	169	282	PF00069.18	1.00E-04	Pkinase
GL50803_11775	1250	1275	PF00023.23	3.90E-04	Ank
GL50803_11775	1429	1452	PF00023.23	4.60E-02	Ank
GL50803_11775	1472	1491	PF00023.23	1.30E-02	Ank
GL50803_11775	1502	1524	PF00023.23	3.30E-03	Ank
GL50803_11866	78	130	PF00583.17	8.90E-05	Acetyltransf_1
GL50803_11953	47	80	PF00400.25	1.80E-11	WD40
GL50803_11953	85	123	PF00400.25	2.80E-11	WD40
GL50803_11953	130	165	PF00400.25	7.60E-09	WD40
GL50803_11953	198	231	PF00400.25	9.50E-07	WD40
GL50803_11953	648	869	PF04053.7	2.10E-08	Coatomer_WDAD
GL50803_11953	1069	1272	PF06957.4	1.10E-10	COPI_C
GL50803_12102	106	180	PF00043.18	5.70E-06	GST_C
GL50803_12102	235	340	PF00647.12	3.80E-30	EF1G
GL50803_137685	15	674	PF02516.7	9.10E-80	STT3
GL50803_14200	130	248	PF00266.12	2.00E-06	Aminotran_5
GL50803_14200	352	598	PF00266.12	1.80E-20	Aminotran_5
GL50803_14223	19	271	PF00069.18	1.60E-36	Pkinase
GL50803_14519	2	394	PF00266.12	1.30E-71	Aminotran_5
GL50803_14581	9	602	PF00012.13	2.10E-162	HSP70
GL50803_14670	19	114	PF00085.13	0,00	Thioredoxin
GL50803_14821	18	103	PF01521.13	2.20E-17	Fe-S_biosyn
GL50803_15196	80	203	PF01592.9	4.50E-45	NifU_N
GL50803_15204	17	201	PF01105.17	3.60E-25	EMP24_GP25L
GL50803_15889	85	259	PF00953.14	3.70E-29	Glycos_transf_4
GL50803_16152	263	535	PF10174.2	9.70E-05	Cast
GL50803_16313	7	249	PF06732.4	4.70E-81	Pescadillo_N
GL50803_16354	38	62	PF00023.23	4.60E-03	Ank
GL50803_16354	69	90	PF00023.23	3.10E-02	Ank
GL50803_16354	614	636	PF00023.23	1.90E-02	Ank
GL50803_16354	651	672	PF00023.23	2.50E-05	Ank
GL50803_16833	85	110	PF07974.6	3.80E-02	EGF_2
GL50803_16833	134	164	PF07974.6	2.40E-02	EGF_2
GL50803_16833	187	219	PF07974.6	3.90E-02	EGF_2
GL50803_16833	224	261	PF07974.6	5.50E-03	EGF_2
GL50803_16833	579	603	PF07974.6	3.90E-05	EGF_2
GL50803_16906	29	373	PF01148.13	1.10E-38	CTP_transf_1
GL50803_16993	25	201	PF01728.12	6.80E-54	FtsJ
GL50803_16993	257	304	PF11861.1	3.30E-03	DUF3381
GL50803_16993	306	460	PF11861.1	6.10E-29	DUF3381
GL50803_16993	854	1054	PF07780.5	4.80E-55	Spb1_C
GL50803_17161	13	242	PF01459.15	1.80E-06	Porin_3
GL50803_17296	84	466	PF07690.9	0,00	MFS_1
GL50803_17342	2	192	PB000845	8.10E-34	Pfam-B_845

GiardiaDB r1.1	PFAM A+B identification (cut off 0.05)				
accession No.	start	end	ID No.	E-value	Protein families
GL50803_17342	215	516	PF07690.9	1.30E-17	MFS_1
GL50803_17510	30	282	PF00069.18	4.60E-28	Pkinase
GL50803_17510	279	407	PF08317.4	5.40E-04	Spc7
GL50803_17510	297	409	PF10473.2	4.00E-04	Cenp-F_leu_zip
GL50803_17510	301	377	PF04582.5	3.90E-03	Reo_sigmaC
GL50803_17510	455	474	PF00023.23	2.70E-03	Ank
GL50803_17510	481	502	PF00023.23	3.30E-04	Ank
GL50803_17510	523	545	PF00023.23	4.90E-02	Ank
GL50803_17510	554	575	PF00023.23	8.80E-04	Ank
GL50803_17510	586	610	PF00023.23	2.20E-04	Ank
GL50803_17510	615	637	PF00023.23	1.10E-03	Ank
GL50803_19230	42	115	PF03656.6	6.30E-03	Pam16
GL50803_2013	115	179	PF00462.17	8.10E-16	Glutaredoxin
GL50803_21118	95	649	PF00501.21	4.50E-80	AMP-binding
GL50803_21118	673	752	PB001299	2.10E-04	Pfam-B_1299
GL50803_23389	549	731	PB015975	1.60E-64	Pfam-B_15975
GL50803_23389	576	614	PF04513.5	4.20E-02	Baculo_PEP_C
GL50803_23389	851	998	PF00233.12	2.20E-05	PDEase_I
GL50803_27266	17	97	PF00111.20	1.60E-10	Fer2
GL50803_29500	33	82	PF00166.14	1.80E-02	Cpn10
GL50803_3470	382	628	PB009274	1.50E-12	Pfam-B_9274
GL50803_3470	382	625	PB001165	5.10E-10	Pfam-B_1165
GL50803_3470	801	922	PF00005.20	3.90E-17	ABC_tran
GL50803_3470	880	978	PB000817	6.30E-07	Pfam-B_817
GL50803_40244	8	94	PF01105.17	3.60E-08	EMP24_GP25L
GL50803_40244	11	91	PF05134.6	2.30E-02	GspL
GL50803_40244	22	58	PF07756.5	1.70E-02	DUF1612
GL50803_5375	37	299	PF00069.18	4.70E-68	Pkinase
GL50803_5375	333	383	PB008998	7.50E-04	Pfam-B_8998
GL50803_5375	358	389	PF12072.1	6.10E-03	DUF3552
GL50803_5744	58	92	PF10559.2	3.20E-13	Plug_translocon
GL50803_5744	93	479	PF00344.13	1.50E-68	SecY
GL50803_7183	59	266	PF00069.18	1.70E-16	Pkinase
GL50803_7183	380	401	PF00023.23	4.00E-02	Ank
GL50803_7183	645	667	PF00023.23	2.30E-03	Ank
GL50803_7183	708	731	PF00023.23	3.90E-03	Ank
GL50803_7188	6	329	PB001790	6.50E-31	Pfam-B_1790
GL50803_7188	324	690	PB001790	0,00	Pfam-B_1790
GL50803_8064	2	43	PF10318.2	3.70E-02	7TM_GPCR_Srh
GL50803_8064	37	132	PF00085.13	3.10E-31	Thioredoxin
GL50803_8163	37	515	PF01368.13	5.60E-22	DHH
GL50803_8163	112	157	PF00571.21	3.50E-06	CBS
GL50803_8163	388	431	PF00571.21	1.30E-03	CBS
GL50803_8163	550	685	PF02833.7	2.00E-08	DHHA2
GL50803_8587	74	237	PF00069.18	8.90E-33	Pkinase
GL50803_8587	287	391	PF00069.18	9.10E-18	Pkinase
GL50803_86855	67	89	PF00023.23	2.00E-03	Ank
GL50803_86855	97	123	PF00023.23	2.00E-03	Ank
GL50803_86855	807	952	PF08614.4	5.90E-03	ATG16
GL50803_86855	899	949	PF04102.5	5.90E-04	SlyX
GL50803_87446	664	769	PF00005.20	7.00E-16	ABC_tran
GL50803_8805	751	825	PB018484	1.30E-04	Pfam-B_18484
GL50803_8805	756	834	PB000079	4.80E-06	Pfam-B_79
GL50803_88245	37	61	PF00023.23	2.30E-02	Ank
GL50803_88245	351	376	PF00023.23	3.50E-02	Ank
GL50803_88245	382	402	PF00023.23	1.70E-04	Ank
GL50803_88245	473	495	PF00023.23	7.20E-03	Ank
GL50803_88245	597	619	PF00023.23	3.60E-03	Ank
GL50803_88245	627	649	PF00023.23	1.50E-03	Ank

GiardiaDB r1.1 accession No.	PFAM A+B identification (cut off 0.05) start	end	ID No.	E-value	Protein families
GL50803_88245	658	681	PF00023.23	2.20E-03	Ank
GL50803_88245	690	709	PF00023.23	3.20E-02	Ank
GL50803_88245	752	774	PF00023.23	4.60E-02	Ank
GL50803_88245	783	803	PF00023.23	5.40E-03	Ank
GL50803_88245	930	954	PF00023.23	3.10E-02	Ank
GL50803_88245	960	986	PF00023.23	1.60E-03	Ank
GL50803_9062	74	196	PF00501.21	3.30E-23	AMP-binding
GL50803_9062	360	688	PF00501.21	1.70E-65	AMP-binding
GL50803_9062	698	803	PB001299	9.70E-10	Pfam-B_1299
GL50803_9062	744	808	PB000908	6.10E-07	Pfam-B_908
GL50803_9062	745	809	PB016442	4.60E-06	Pfam-B_16442
GL50803_91099	164	191	PF07974.6	4.40E-03	EGF_2
GL50803_91099	434	458	PF07974.6	4.30E-02	EGF_2
GL50803_91099	538	561	PF00008.20	8.50E-04	EGF
GL50803_91099	695	723	PF07974.6	2.30E-03	EGF_2
GL50803_91099	754	777	PF07974.6	1.60E-02	EGF_2
GL50803_91099	962	993	PF07974.6	7.30E-03	EGF_2
GL50803_91099	1216	1239	PF00008.20	2.00E-02	EGF
GL50803_91099	1313	1337	PF07974.6	5.90E-03	EGF_2
GL50803_94322	617	640	PF01391.11	1.60E-02	Collagen
GL50803_94322	784	857	PF00004.22	3.70E-10	AAA
GL50803_94658	2	81	PF11543.1	6.50E-08	UN_NPL4
GL50803_94658	252	515	PF05021.8	8.50E-13	NPL4
GL50803_9478	13	149	PF00675.13	7.60E-10	Peptidase_M16
GL50803_9719	14	215	PF00724.13	1.50E-07	Oxidored_FMN
GL50803_9719	225	344	PF00724.13	8.60E-05	Oxidored_FMN
GL50803_9719	300	375	PF00189.13	1.20E-02	Ribosomal_S3_C
GL50803_9751	264	317	PF00226.24	1.70E-15	DnaJ
GL50803_10330	15	61	PF01683.11	2.70E-04	EB
GL50803_10330	24	67	PF01826.10	1.70E-04	TIL
GL50803_10330	37	56	PF07974.6	1.40E-02	EGF_2
GL50803_10330	120	146	PF07974.6	3.80E-03	EGF_2
GL50803_10330	227	251	PF07974.6	6.90E-04	EGF_2
GL50803_103454	14	123	PF03302.6	6.80E-09	VSP
GL50803_103454	112	230	PF03302.6	8.30E-04	VSP
GL50803_103454	237	376	PF03302.6	1.40E-03	VSP
GL50803_103454	334	717	PF03302.6	3.20E-38	VSP
GL50803_103454	679	721	PB016930	5.80E-07	Pfam-B_16930
GL50803_103454	689	714	PF08693.3	3.40E-03	SKG6
GL50803_112079	3	225	PF00091.18	0,00	Tubulin
GL50803_112079	263	392	PF03953.10	2.40E-51	Tubulin_C
GL50803_112312	5	216	PF00009.20	6.60E-56	GTP_EFTU
GL50803_112312	249	315	PF03144.18	2.10E-16	GTP_EFTU_D2
GL50803_112312	324	430	PF03143.10	1.40E-28	GTP_EFTU_D3
GL50803_11237	230	427	PB011818	3.70E-49	Pfam-B_11818
GL50803_11359	3	40	PF08071.5	1.00E-18	RS4NT
GL50803_11359	47	89	PF01479.18	2.00E-05	S4
GL50803_11359	95	169	PF00900.13	3.40E-29	Ribosomal_S4e
GL50803_11359	178	201	PF00467.22	1.20E-03	KOW
GL50803_11470	68	209	PF03302.6	3.30E-03	VSP
GL50803_11470	213	299	PF03302.6	1.70E-05	VSP
GL50803_11470	346	534	PF03302.6	3.10E-26	VSP
GL50803_11470	532	673	PF03302.6	2.10E-50	VSP
GL50803_11470	622	661	PF05808.4	1.30E-02	Podoplanin
GL50803_11470	622	668	PB016930	3.90E-04	Pfam-B_16930
GL50803_11470	624	663	PB020000	1.40E-04	Pfam-B_20000
GL50803_11521	16	184	PF03302.6	9.20E-06	VSP
GL50803_11521	171	257	PF03302.6	1.50E-05	VSP
GL50803_11521	308	491	PF03302.6	9.20E-30	VSP

GiardiaDB r1.1	PFAM A+B identification (cut off 0.05)				
accession No.	start	end	ID No.	E-value	Protein families
GL50803_11521	487	628	PF03302.6	1.70E-52	VSP
GL50803_11521	561	618	PB020000	2.60E-06	Pfam-B_20000
GL50803_11521	570	615	PF05808.4	3.80E-02	Podoplanin
GL50803_11521	584	625	PB016930	1.50E-04	Pfam-B_16930
GL50803_11557	61	123	PB011995	0,00	Pfam-B_11995
GL50803_13561	7	162	PB001387	1.40E-16	Pfam-B_1387
GL50803_13561	95	122	PF10587.2	2.70E-08	EF-1_beta_acid
GL50803_13561	131	219	PF00736.12	2.50E-28	EF1_GNE
GL50803_136020	3	222	PF00091.18	7.40E-72	Tubulin
GL50803_136020	261	382	PF03953.10	2.10E-48	Tubulin_C
GL50803_13922	1	334	PB001790	2.40E-33	Pfam-B_1790
GL50803_13922	326	546	PB001790	0,00	Pfam-B_1790
GL50803_14469	150	234	PF00957.14	2.00E-17	Synaptobrevin
GL50803_14469	174	232	PF02009.9	1.60E-02	Rifin_STEVOR
GL50803_14469	192	238	PF08412.3	4.20E-02	Ion_trans_N
GL50803_14660	9	115	PF03710.8	2.00E-02	GlnE
GL50803_14660	18	116	PF03179.8	1.60E-12	V-ATPase_G
GL50803_14993	69	315	PF00365.13	3.60E-21	PFK
GL50803_15097	31	78	PF00191.13	8.80E-05	Annexin
GL50803_15097	88	143	PF00191.13	5.20E-08	Annexin
GL50803_15097	171	226	PF00191.13	1.50E-03	Annexin
GL50803_16076	6	140	PF00578.14	0,00	AhpC-TSA
GL50803_16076	161	196	PF10417.2	4.50E-08	1-cysPrx_C
GL50803_16424	45	169	PF10248.2	3.10E-04	Mlf1IP
GL50803_16477	114	140	PF00008.20	1.10E-03	EGF
GL50803_16477	217	240	PF07974.6	4.90E-03	EGF_2
GL50803_16477	352	377	PF07974.6	1.80E-05	EGF_2
GL50803_16477	457	480	PF07974.6	3.00E-04	EGF_2
GL50803_16477	554	578	PF07974.6	4.00E-02	EGF_2
GL50803_16824	17	288	PF00069.18	1.40E-39	Pkinase
GL50803_16824	415	437	PF00023.23	2.00E-05	Ank
GL50803_16824	451	471	PF00023.23	3.60E-03	Ank
GL50803_16824	512	530	PF00023.23	1.20E-03	Ank
GL50803_16824	610	632	PF00023.23	6.20E-03	Ank
GL50803_16824	640	664	PF00023.23	9.90E-04	Ank
GL50803_16824	683	702	PF00023.23	4.70E-03	Ank
GL50803_16824	712	732	PF00023.23	2.70E-04	Ank
GL50803_16824	745	775	PF00023.23	3.80E-03	Ank
GL50803_17121	30	652	PF00012.13	6.20E-232	HSP70
GL50803_17121	542	651	PB002482	1.60E-05	Pfam-B_2482
GL50803_17165	623	733	PF00005.20	4.50E-07	ABC_tran
GL50803_17236	26	206	PF07786.5	2.70E-02	DUF1624
GL50803_17288	1596	1618	PF00023.23	4.80E-04	Ank
GL50803_17288	1718	1741	PF00023.23	9.30E-03	Ank
GL50803_17288	1754	1773	PF00023.23	2.10E-02	Ank
GL50803_17288	1790	1808	PF00023.23	8.90E-04	Ank
GL50803_17328	807	1127	PF03302.6	2.50E-10	VSP
GL50803_17411	31	558	PF00118.17	1.70E-142	Cpn60_TCP1
GL50803_17476	21	279	PB001790	1.00E-08	Pfam-B_1790
GL50803_17476	955	1264	PB001790	8.10E-30	Pfam-B_1790
GL50803_17476	1780	2001	PF03302.6	3.50E-10	VSP
GL50803_17476	1996	2051	PF03302.6	1.50E-03	VSP
GL50803_18470	29	392	PF01496.12	1.20E-15	V_ATPase_I
GL50803_18470	425	919	PF01496.12	7.20E-139	V_ATPase_I
GL50803_1937	2	93	PB004452	6.70E-20	Pfam-B_4452
GL50803_23492	440	462	PF00023.23	8.00E-05	Ank
GL50803_23492	503	525	PF00023.23	7.90E-04	Ank
GL50803_23492	535	554	PF00023.23	2.00E-04	Ank
GL50803_23492	565	587	PF00023.23	2.40E-03	Ank

GiardiaDB r1.1	PFAM A+B identification (cut off 0.05)				
accession No.	start	end	ID No.	E-value	Protein families
GL50803_23492	597	619	PF00023.23	1.00E-03	Ank
GL50803_23492	658	681	PF00023.23	3.10E-05	Ank
GL50803_23492	690	709	PF00023.23	4.70E-02	Ank
GL50803_28379	109	392	PF00664.16	2.70E-10	ABC_membrane
GL50803_28379	677	773	PF00005.20	2.40E-11	ABC_tran
GL50803_28379	900	1146	PF00664.16	4.00E-18	ABC_membrane
GL50803_28379	1251	1282	PF08477.6	6.60E-03	Miro
GL50803_28379	1301	1417	PF00005.20	1.30E-12	ABC_tran
GL50803_28379	1438	1475	PB011559	1.50E-05	Pfam-B_11559
GL50803_28962	74	184	PF02430.8	1.70E-03	AMA-1
GL50803_28962	99	179	PF02009.9	1.10E-02	Rifin_STEVOR
GL50803_28962	105	193	PF12259.1	2.90E-04	DUF3609
GL50803_28962	122	193	PF08374.4	7.10E-03	Protocadherin
GL50803_28962	127	184	PF06024.5	1.90E-04	DUF912
GL50803_28962	139	188	PF05510.6	4.30E-02	Sarcoglycan_2
GL50803_28962	139	199	PB006006	4.50E-04	Pfam-B_6006
GL50803_28962	141	222	PF02480.9	1.80E-06	Herpes_gE
GL50803_28962	141	181	PF05454.4	5.00E-02	DAG1
GL50803_28962	143	183	PF06583.5	9.90E-05	Neogenin_C
GL50803_28962	145	197	PF04478.5	3.00E-03	Mid2
GL50803_28962	146	193	PB011119	8.30E-06	Pfam-B_11119
GL50803_28962	147	198	PF07423.4	3.60E-02	DUF1510
GL50803_28962	148	232	PB019284	3.00E-04	Pfam-B_19284
GL50803_28962	154	206	PF00335.13	1.10E-02	Tetraspannin
GL50803_28962	154	231	PF03748.7	4.90E-02	FliL
GL50803_28962	155	190	PF05961.4	2.00E-02	Chordopox_A13L
GL50803_32999	2	74	PF06009.5	4.70E-02	Laminin_II
GL50803_32999	2	36	PF12329.1	4.90E-02	TMF_DNA_bd
GL50803_32999	10	245	PF00038.14	9.60E-03	Filament
GL50803_32999	14	173	PF05701.4	3.30E-04	DUF827
GL50803_32999	50	153	PF03962.8	7.20E-03	Mnd1
GL50803_32999	84	127	PF04977.8	3.20E-02	DivIC
GL50803_32999	91	173	PB000039	5.40E-05	Pfam-B_39
GL50803_32999	97	250	PB016660	5.10E-04	Pfam-B_16660
GL50803_32999	122	174	PF11559.1	3.80E-02	ADIP
GL50803_32999	261	382	PF04156.7	8.30E-04	IncA
GL50803_32999	269	396	PF10174.2	1.20E-02	Cast
GL50803_32999	279	344	PB000381	4.70E-04	Pfam-B_381
GL50803_32999	298	349	PF06156.6	4.00E-02	DUF972
GL50803_32999	302	363	PF12329.1	6.10E-04	TMF_DNA_bd
GL50803_42285	679	969	PB003450	2.50E-22	Pfam-B_3450
GL50803_42285	2594	2895	PF08393.6	3.90E-38	DHC_N2
GL50803_42285	3265	3336	PF07728.7	1.60E-03	AAA_5
GL50803_5649	51	99	PF00191.13	3.30E-04	Annexin
GL50803_5649	111	162	PF00191.13	2.60E-02	Annexin
GL50803_5649	189	240	PF00191.13	5.50E-03	Annexin
GL50803_6733	129	177	PF01529.13	2.50E-20	zf-DHHC
GL50803_7110	11	79	PF00240.16	3.50E-34	ubiquitin
GL50803_7110	16	71	PB014891	7.30E-06	Pfam-B_14891
GL50803_7110	31	75	PB013282	3.10E-04	Pfam-B_13282
GL50803_7532	9	159	PB010324	0,00	Pfam-B_10324
GL50803_7532	12	74	PF02874.16	1.30E-09	ATP-synt_ab_N
GL50803_7532	234	501	PF00006.18	2.20E-96	ATP-synt_ab
GL50803_7532	524	654	PF00306.20	4.00E-13	ATP-synt_ab_C
GL50803_8559	18	80	PF00137.14	6.00E-13	ATP-synt_C
GL50803_8559	97	162	PF00137.14	1.50E-20	ATP-synt_C
GL50803_8564	642	699	PF10211.2	3.40E-03	Ax_dynein_light
GL50803_8589	53	336	PF02383.11	6.20E-44	Syja_N
GL50803_8903	50	189	PF07002.9	1.20E-32	Copine

GiardiaDB r1.1	PFAM A+B identification (cut off 0.05)				
accession No.	start	end	ID No.	E-value	Protein families
GL50803_93736	127	201	PB001786	3.70E-10	Pfam-B_1786
GL50803_93736	201	621	PB001786	1.90E-116	Pfam-B_1786
GL50803_93736	1252	1392	PF08393.6	3.10E-02	DHC_N2
GL50803_93736	1485	1615	PF08393.6	0,00	DHC_N2
GL50803_93736	1865	1938	PF07728.7	2.30E-03	AAA_5
GL50803_93736	2185	2329	PF07728.7	1.10E-07	AAA_5
GL50803_93736	2360	2658	PB000014	2.40E-27	Pfam-B_14
GL50803_93736	2637	2871	PB017424	7.50E-43	Pfam-B_17424
GL50803_93736	3170	3360	PB000014	1.00E-10	Pfam-B_14
GL50803_9503	71	303	PF00561.13	1.00E-11	Abhydrolase_1
GL50803_96670	129	197	PF00690.19	1.70E-19	Cation_ATPase_N
GL50803_96670	222	454	PF00122.13	6.40E-58	E1-E2_ATPase
GL50803_96670	764	832	PF00702.19	5.30E-10	Hydrolase
GL50803_96670	915	980	PF00702.19	5.90E-06	Hydrolase
GL50803_96670	1049	1294	PF00689.14	1.60E-47	Cation_ATPase_C
GL50803_9780	9	89	PF08400.3	2.20E-02	phage_tail_N
GL50803_88765	6	615	PF00012.13	3.70E-249	HSP70
GL50803_88765	515	613	PB002482	3.80E-07	Pfam-B_2482
GL50803_88765	614	657	PB008944	1.00E-05	Pfam-B_8944
GL50803_11654	8	67	PF00191.13	7.10E-09	Annexin
GL50803_11654	159	206	PF00191.13	4.40E-02	Annexin
GL50803_137618	133	319	PF03302.6	1.20E-09	VSP
GL50803_137618	455	853	PF03302.6	1.20E-130	VSP
GL50803_137618	793	850	PB016930	5.60E-05	Pfam-B_16930
GL50803_6430	7	241	PF00244.13	2.10E-104	14.3.2003
GL50803_21411	5	271	PB002445	2.60E-18	Pfam-B_2445
GL50803_21411	610	747	PF00005.20	1.70E-15	ABC_tran
GL50803_21411	1675	1733	PB003932	4.60E-04	Pfam-B_3932
GL50803_21411	1691	1818	PF00005.20	7.70E-14	ABC_tran
GL50803_13000	17	346	PF01992.9	1.60E-32	vATP-synt_AC39
GL50803_29327	18	170	PF03357.14	8.40E-15	Snf7
GL50803_29327	38	133	PF01406.12	3.10E-02	tRNA-synt_1e
GL50803_14225	53	246	PB001790	3.40E-06	Pfam-B_1790
GL50803_6497	27	79	PF00753.20	6.60E-07	Lactamase_B
GL50803_7796	18	67	PF00191.13	5.20E-08	Annexin
GL50803_10311	7	147	PF02729.14	1.40E-41	OTCace_N
GL50803_10311	159	319	PF00185.17	2.30E-46	OTCace
GL50803_23833	59	136	PF03635.10	9.90E-08	Vps35
GL50803_23833	169	349	PF03635.10	6.10E-24	Vps35
GL50803_23833	651	747	PF03635.10	5.30E-04	Vps35
