# Supplementary material for: The Minimal Proteome in the Reduced Mitochondrion of the Parasitic Protist Giardia intestinalis
Source: PLoS One. 2011 Feb 24;6(2):e17285. doi: 10.1371/journal.pone.0017285 (PMC3044749; doi:10.1371/journal.pone.0017285)
Supplement: Table S4 — Predictions of cellular localization. (PDF) [file pone.0017285.s013.pdf]

Table S4. Predictions of cellular localization

GiardiaDB r1.1		Cell localization									
		TargetP						SignalP		PSORTII
Annotatio	Accession No.	Len (aa)	0.938	SP	other	Loc	RC	Loc	**Sprob**	mit%
PAM18	AACB02000023-5-3211	106	0.518	0.152	0.219	M	4	N	0.102	30.4%
Hypothetica	GL50803_10016	317	0.077	0.940	0.014	S	1	Y	0.770	21.7%
Kinesin-3	GL50803_102101	1026	0.303	0.086	0.443	O	5	N	0.000	26.1%
Ornithine c	GL50803_10311	327	0.091	0.436	0.555	O	5	N	0.346	8.7%
Tenascin p	GL50803_10330	257	0.057	0.827	0.310	S	3	Y	0.978	11.1%
High cystei	GL50803_103454	736	0.074	0.744	0.289	S	3	Y	0.999	*
Chaperonir	GL50803_103891	547	0.158	0.053	0.840	O	2	N	0.000	*
Hypothetica	GL50803_111809	474	0.121	0.063	0.876	O	2	N	0.000	*
Alpha-tubu	GL50803_112079	454	0.071	0.122	0.871	O	2	N	0.005	*
Elongation	GL50803_112312	442	0.061	0.071	0.923	O	1	N	0.001	4.3%
Hypothetica	GL50803_11237	428	0.089	0.055	0.922	O	1	N	0.000	8.7%
Ribosomal	GL50803_11359	268	0.486	0.061	0.521	O	5	N	0.003	17.4%
Cysteine pı	GL50803_113656	636	0.185	0.623	0.288	S	4	Y	0.996	*
Long chain	GL50803_113892	758	0.069	0.066	0.940	O	1	N	0.000	26.1%
High cystei	GL50803_114042	827	0.100	0.705	0.280	S	3	Y	0.993	*
VSP with II	GL50803_11470	673	0.068	0.366	0.628	O	4	N	0.004	*
Hypothetica	GL50803_114777	656	0.462	0.167	0.341	M	5	N	0.043	4.3%
VSP	GL50803_11521	628	0.034	0.944	0.075	S	1	Y	0.999	*
Hypothetica	GL50803_11557	610	0.1	0.11	0.799	O	2	N	0.008	17.4%
Alpha-1 gia	GL50803_11654	295	0.058	0.086	0.938	O	1	N	0	13.0%
Kinase, NE	GL50803_11775	1618	0.072	0.052	0.950	O	1	N	0.000	17.4%
Hypothetica	GL50803_11866	156	0.071	0.126	0.897	O	2	N	0.003	21.7%
Coatomer a	GL50803_11953	1277	0.170	0.095	0.749	O	3	N	0.000	*
Elongation	GL50803_12102	402	0.396	0.079	0.376	M	5	N	0.039	13.0%
Hypothetica	GL50803_12999	224	0.572	0.198	0.252	M	4	Y	0.708	*
Vacuolar A	GL50803_13000	351	0.126	0.105	0.78	O	2	N	0	13.0%
Hypothetica	GL50803_13288	708	0.104	0.13	0.851	O	2	N	0	8.7%
Hypothetica	GL50803_13413	183	0.351	0.187	0.368	O	5	N	0.000	11.1%
Translation	GL50803_13561	220	0.16	0.094	0.806	O	2	N	0	13.0%
Beta tubulii	GL50803_136020	447	0.099	0.089	0.896	O	2	N	0.000	*
VSP	GL50803_137618	853	0.052	0.109	0.904	O	2	N	0.007	4.3%
Hypothetica	GL50803_137685	719	0.092	0.655	0.174	S	3	N	0.269	*
Hypothetica	GL50803_137746	555	0.392	0.036	0.644	O	4	N	0.000	4.3%
Hypothetica	GL50803_13922	1087	0.012	0.97	0.069	S	1	Y	0.999	*
Hypothetica	GL50803_14164	811	0.018	0.225	0.913	O	2	N	0.029	13.0%
Molybdenu	GL50803_14200	619	0.297	0.196	0.475	O	5	N	0.062	22.2%
Kinase, NE	GL50803_14223	302	0.126	0.081	0.834	O	2	N	0.000	13.0%
CXC-rich p	GL50803_14225	1805	0.026	0.957	0.083	S	1	Y	0.997	*
Hypothetica	GL50803_14278	666	0.266	0.057	0.688	O	3	N	0.000	13.0%
Synaptobre	GL50803_14469	239	0.544	0.038	0.588	O	5	N	0	*
Cysteine de	GL50803_14519	433	0.187	0.034	0.861	O	2	N	0.000	*
Chaperone	GL50803_14581	640	0.129	0.177	0.685	O	3	N	0.045	13.0%
Hypothetica	GL50803_14660	118	0.082	0.045	0.932	O	1	N	0	34.8%
Protein disı	GL50803_14670	116	0.123	0.848	0.103	S	2	Y	0.997	22.2%
HesB doma	GL50803_14821	131	0.430	0.080	0.506	O	5	N	0.013	34.8%
Hypothetica	GL50803_14845	286	0.081	0.215	0.593	O	4	N	0.035	4.3%
Hypothetica	GL50803_14939	314	0.725	0.093	0.105	M	2	Y	0.895	30.4%
Pyrophospl	GL50803_14993	544	0.721	0.043	0.338	M	4	N	0	34.8%
Hypothetica	GL50803_15084	1228	0.235	0.107	0.678	O	3	N	0.001	*
Alpha-14 g	GL50803_15097	337	0.124	0.141	0.722	O	3	N	0.017	4.3%
NifU-like pı	GL50803_15196	212	0.061	0.173	0.876	M	2	N	0.000	17.4%
ERP3	GL50803_15204	206	0.071	0.844	0.247	S	3	Y	0.698	*
UDP-N-ace	GL50803_15889	366	0.022	0.989	0.025	S	1	Y	0.998	4.3%
Hypothetica	GL50803_15985	263	0.537	0.224	0.112	M	4	N	0.004	13.0%
Peroxiredo	GL50803_16076	201	0.084	0.186	0.789	O	2	N	0.000	8.7%
Coiled-coil	GL50803_16152	1086	0.257	0.009	0.908	O	2	N	0.000	*
Hypothetica	GL50803_16313	581	0.688	0.021	0.456	M	4	N	0.000	17.4%
Protein 21.	GL50803_16354	718	0.054	0.068	0.946	O	1	N	0.000	4.3%
Hypothetica	GL50803_16424	252	0.164	0.070	0.829	O	2	N	0.000	26.1%
Hypothetica	GL50803_16430	2612	0.226	0.016	0.86	O	2	N	0.001	8.7%
Tenascin-3	GL50803_16477	582	0.028	0.9	0.162	S	2	Y	0.945	17.4%
Kinase, NE	GL50803_16824	823	0.313	0.052	0.714	O	3	N	0.000	*
Tenascin-li	GL50803_16833	605	0.079	0.764	0.354	S	3	Y	0.990	*
Phosphatid	GL50803_16906	376	0.842	0.135	0.044	M	2	N	0.002	8.7%
FtsJ cell di	GL50803_16993	1084	0.369	0.107	0.519	O	5	N	0.000	17.4%
Hypothetica	GL50803_16998	608	0.227	0.036	0.829	O	2	N	0.000	17.4%
Bip	GL50803_17121	677	0.027	0.742	0.334	S	3	Y	0.898	11.1%
Hypothetica	GL50803_17161	355	0.120	0.087	0.870	O	2	N	0.000	13.0%

GiardiaDB r1.1		Cell localization								
		TargetP						SignalP		PSORTII
**Annotatio**	**Accession No.**	Len (aa)	0.938	SP	other	Loc	RC	Loc	**Sprob**	mit%
ABC transp	GL50803_17165	878	0.315	0.296	0.387	O	5	N	0.137	4.3%
Hypothetica	GL50803_17236	673	0.462	0.215	0.257	M	4	N	0.002	*
Protein 21.	GL50803_17288	1850	0.196	0.089	0.705	O	3	N	0.084	4.3%
Hypothetica	GL50803_17296	540	0.715	0.062	0.323	M	4	N	0.000	*
High cystei	GL50803_17328	1299	0.061	0.918	0.077	S	1	Y	0.999	*
Hypothetica	GL50803_17342	659	0.475	0.208	0.300	M	5	N	0.043	*
TCP-1 cha	GL50803_17411	564	0.397	0.05	0.687	O	4	N	0	*
CXC-rich p	GL50803_17476	2169	0.061	0.861	0.068	S	2	Y	1	4.3%
Kinase, NE	GL50803_17510	685	0.076	0.137	0.877	O	2	N	0.075	17.4%
Vacuolar p	GL50803_18470	933	0.355	0.106	0.426	O	5	N	0.023	4.3%
Hypothetica	GL50803_19230	143	0.085	0.173	0.764	O	3	N	0.067	13.0%
Hypothetica	GL50803_1937	94	0.006	0.997	0.046	S	1	N	0.011	*
Glutaredox	GL50803_2013	202	0.121	0.391	0.653	O	4	N	0.124	13.0%
Long chain	GL50803_21118	765	0.251	0.062	0.679	O	3	N	0.000	26.1%
ABC transp	GL50803_21411	1978	0.112	0.887	0.034	S	2	N	0.056	*
Spindle pol	GL50803_21444	588	0.233	0.036	0.842	O	2	N	0.000	21.7%
Hypothetica	GL50803_23389	1102	0.045	0.534	0.638	O	5	N	0.012	*
Protein 21.	GL50803_23492	743	0.098	0.047	0.921	O	1	N	0	30.4%
Vacuolar p	GL50803_23833	765	0.118	0.104	0.81	O	2	N	0	11.1%
[2Fe-2S] fe	GL50803_27266	133	0.737	0.031	0.209	M	3	N	0.027	47.8%
Multidrug r	GL50803_28379	1503	0.207	0.024	0.821	O	2	N	0	*
Hypothetica	GL50803_28962	307	0.045	0.907	0.163	S	2	Y	0.999	4.3%
Hypothetica	GL50803_29327	200	0.132	0.04	0.902	O	2	N	0	17.4%
Hypothetica	GL50803_29500	103	0.109	0.28	0.636	O	4	Y	0.711	8.7%
Hypothetica	GL50803_3021	307	0.177	0.081	0.772	O	3	N	0.000	13.0%
Hypothetica	GL50803_32999	461	0.095	0.049	0.922	O	1	N	0.000	13.0%
ABC transp	GL50803_3470	1113	0.890	0.104	0.057	M	2	N	0.000	*
Hypothetica	GL50803_3491	278	0.154	0.057	0.848	O	2	N	0	30.4%
P24, putati	GL50803_40244	99	0.114	0.156	0.873	O	2	N	0.000	13.0%
Ciliary dyne	GL50803_42285	7449	*	*	*	*	*	N	0	*
Hypothetica	GL50803_4768	594	0.843	0.022	0.312	M	3	N	0.000	56.5%
Kinase, NE	GL50803_5375	405	0.419	0.049	0.592	O	5	N	0.035	17.4%
Alpha-10 g	GL50803_5649	328	0.113	0.063	0.899	O	2	N	0	8.7%
Sec61-alph	GL50803_5744	490	0.599	0.168	0.180	M	3	N	0.056	22.2%
14-3-3 prot	GL50803_6430	248	0.067	0.104	0.922	O	1	N	0	13.0%
Metal-depe	GL50803_6497	334	0.063	0.419	0.646	O	4	N	0.204	13.0%
Hypothetica	GL50803_6617	562	0.056	0.941	0.057	S	1	Y	0.999	*
Zinc finger	GL50803_6733	339	0.036	0.977	0.067	S	1	N	0	22.2%
Ubiquitin	GL50803_7110	82	0.097	0.330	0.457	O	5	N	0.008	17.4%
Kinase, NE	GL50803_7183	808	0.142	0.121	0.778	O	2	N	0.000	13.0%
Hypothetica	GL50803_7188	1103	0.064	0.787	0.186	S	2	Y	0.994	13.0%
Hypothetica	GL50803_7242	247	0.186	0.237	0.530	O	4	N	0.000	22.2%
Hypothetica	GL50803_7244	192	0.107	0.191	0.804	O	2	N	0.000	11.1%
Vacuolar A	GL50803_7532	655	0.06	0.118	0.873	O	2	N	0.038	17.4%
Alpha-2 gia	GL50803_7796	296	0.074	0.084	0.91	O	1	N	0	17.4%
Protein disu	GL50803_8064	134	0.122	0.622	0.206	S	3	Y	0.892	13.0%
Manganese	GL50803_8163	695	0.357	0.111	0.644	O	4	N	0.003	21.7%
Vacuolar A	GL50803_8559	173	0.03	0.391	0.734	O	4	N	0.142	11.1%
Coiled-coil	GL50803_8564	1680	0.335	0.055	0.556	O	4	N	0	*
Kinase, AG	GL50803_8587	482	0.152	0.033	0.886	O	2	N	0.000	4.3%
Suppresso	GL50803_8589	702	0.066	0.110	0.911	O	1	N	0.000	11.1%
Protein 21.	GL50803_86855	1111	0.083	0.096	0.906	O	1	N	0.000	8.7%
ABC transp	GL50803_87446	957	0.300	0.316	0.331	O	5	N	0.000	*
Kinase, SC	GL50803_8805	936	0.245	0.108	0.615	O	4	N	0.000	11.1%
Protein 21.	GL50803_88245	1226	0.113	0.165	0.701	O	3	N	0.086	17.4%
Cytosolic H	GL50803_88765	664	0.073	0.126	0.856	O	2	N	0.01	4.3%
Copine I	GL50803_8903	254	0.233	0.064	0.725	O	3	N	0.000	43.5%
Long chain	GL50803_9062	853	0.053	0.052	0.941	O	1	N	0.000	22.2%
High cystei	GL50803_91099	1374	0.075	0.662	0.398	S	4	Y	0.987	13.0%
Hypothetica	GL50803_9296	241	0.451	0.083	0.352	M	5	Y	0.664	56.5%
Dynein hea	GL50803_93736	4773	*	*	*	*	*	N	0.001	13.0%
Spastin	GL50803_94322	1118	0.07	0.07	0.93	O	1	N	0	8.7%
Hypothetica	GL50803_94658	527	0.158	0.076	0.805	O	2	N	0.000	13.0%
Alpha mito	GL50803_9478	0	0	0	0	O	0	N	0	4.3%
Hypothetica	GL50803_9503	307	0.052	0.096	0.929	O	1	N	0.000	8.7%
Coiled-coil	GL50803_9515	1374	0.063	0.104	0.919	O	1	N	0.017	*
Potassium-	GL50803_96670	1335	0.39	0.04	0.713	O	4	N	0	4.3%
NADH oxid	GL50803_9719	429	0.356	0.052	0.676	O	4	N	0.005	8.7%
Chaperone	GL50803_9751	347	0.133	0.051	0.885	O	2	N	0.000	13.0%
Hypothetica	GL50803_9780	249	0.096	0.852	0.094	S	2	Y	0.999	11.1%
Hypothetica	GL50803_9861	385	0.082	0.083	0.892	O	1	N	0.000	4.3%
