# Supplementary material for: The Minimal Proteome in the Reduced Mitochondrion of the Parasitic Protist Giardia intestinalis
Source: PLoS One. 2011 Feb 24;6(2):e17285. doi: 10.1371/journal.pone.0017285 (PMC3044749; doi:10.1371/journal.pone.0017285)
Supplement: Table S5 — Orthology phylogenetic profililng. Genomes of G. intestinalis and Rickettsia typhi were compared using orthology phylogenetic profile tool at GiardiaDB. (PDF) [file pone.0017285.s014.pdf]

Supplemental Table 5. Orthology phylogenetic profililng. Genomes of *G. intestinalis* and *Rickettsia typhi* were compared using orthology phylogenetic profile tool at GiardiaDB (http://www.orthomcl.org/cgi-bin/OrthoMclWeb.cgi)

Accession number	Identification	PsortII %mito	TargetP	SignalP
GL50803_10091	Ribosomal protein L23	17.4	26.3	N
GL50803_101501	Seryl-tRNA synthetase	21.7	16.7	N
GL50803_102963	tRNA 2-methylthioadenosine synthase	4.3	7	N
GL50803_103887	Dicer	17.4	8	N
GL50803_103891	Chaperonin 60	ND	15.8	N
GL50803_104173	Isoleucyl-tRNA synthetase	ND	6.5	N
GL50803_10521	Arginyl-tRNA synthetase	17.4	4.7	N
GL50803_10969	Nucleotide-binding protein 1	21.7	25.7	N
GL50803_11301	Nucleoside diphosphate kinase	4.3	31	N
GL50803_113021	Acetyl-CoA carboxylase/pyruvate carboxylase fusion protein, putative	17.4	8	N
GL50803_113610	GlcNAc-PI synthesis protein	13	6.4	N
GL50803_113873	Hypothetical protein	ND	33.7	N
GL50803_114246	GTP-binding protein, putative	13	10.5	N
GL50803_12082	Hypothetical protein	13	9.5	N
GL50803_12108	Threonine dehydratase lateral transfer candidate	ND	7	N
GL50803_12162	Phenylalanyl-tRNA synthetase alpha chain	4.3	15.8	N
GL50803_1376	GrpE	39.1	82.7	N
GL50803_13864	Heat shock protein HSP 90-alpha	17.4	22.4	N
GL50803_13889	Maf-like protein yhdE	11.1	37.7	Y
GL50803_13998	Threonyl-tRNA synthetase	8.7	32.9	N
GL50803_14275	hypothetical protein	8.7	31.6	N
GL50803_14519	Cysteine desulfurase	ND	18.7	N
GL50803_14521	Peroxiredoxin 1	8.7	8.4	N
GL50803_14702	RRNA biogenesis protein RRP5	4.3	59.7	N
GL50803_14821	HesB domain-containing protein	34.8	43	N
GL50803_14842	Dimethyladenosine transferase	4.3	14.2	N
GL50803_14856	Signal recognition particle receptor	13	6.3	N
GL50803_15048	ATP-dependent RNA helicase-like protein	4.3	10.6	N
GL50803_15156	SRP GTPase	ND	13.4	N
GL50803_15196	NifU-like protein	17.4	6.1	N
GL50803_15228	Ribosomal protein S15A	34.8	64.7	N
GL50803_15247	Grp94/Hsp90	ND	7.4	Y
GL50803_15256	Undecaprenyl pyrophosphate synthase	13	26.5	N
GL50803_15260	Ribosomal protein S15	17.4	49.6	N
GL50803_15313	Pseudouridine synthase, putative	17.4	26	Y
GL50803_15324	Nucleotide-binding protein 1	39.1	32.9	N
GL50803_15380	CDC8 Thymidylate kinase	34.8	28	N
GL50803_15383	Peroxiredoxin 1	ND	4.9	Y
GL50803_15551	Ribosomal protein S18	17.4	9.3	N
GL50803_16064	tRNA-nucleotidyltransferase, putative	17.4	11.3	N
GL50803_16069	Phosphoacetylglucosamine mutase	17.4	12.4	N
GL50803_16076	Peroxiredoxin 1	8.7	8.4	N
GL50803_16086	Ribosomal protein L2	55.2	94.6	N
GL50803_16149	Mlh1-like protein	4.3	6.8	N
GL50803_16223	DNA-directed RNA polymerase subunit A'	13	86.6	N
GL50803_16311	Centromere/microtubule binding protein CBF5	ND	6.3	N
GL50803_16328	DRAP deaminase	8.7	5.9	N
GL50803_16483	Dephospho-CoA kinase	13	29.5	Y
GL50803_16803	Hypothetical protein	8.7	1.1	Y
GL50803_16880	Multidrug resistance protein B	ND	7.3	N
GL50803_16975	DNA topoisomerase II	ND	37.7	N
GL50803_17030	Chaperone protein dnaJ	37.8	34.9	N
GL50803_17187	DNA-directed RNA polymerase subunit B'	ND	19.5	N
GL50803_17305	Multidrug resistance protein B	ND	5.2	N
GL50803_17389	SUA5 protein	11.1	30.9	Y
GL50803_17420	O-sialoglycoprotein endopeptidase	8.7	12.1	N
GL50803_17427	Hypothetical protein	ND	3.6	Y
GL50803_17448	DNA-directed RNA polymerase RPB2	ND	8	N
GL50803_17520	ClpB protein	8.7	20.9	N
GL50803_17587	CTP synthase	17.4	3.8	N
GL50803_2013	Glutaredoxin-related protein	13	12.1	N
GL50803_22204	Methionyl-tRNA synthetase	21.7	8.6	N
GL50803_22808	Histidyl-tRNA synthetase	4.3	1.8	N
GL50803_24636	Replication factor C, subunit 3	13	5.8	N
GL50803_2486	Cytosine deaminase, putative	17.4	5.9	N
GL50803_27266	[2Fe-2S] ferredoxin	47.8	73.8	N
GL50803_27614	Ribose 5-phosphate isomerase	17.4	37.8	N
GL50803_28234	Adenylate kinase	17.4	8.8	N
GL50803_32697	Hypothetical protein	13	8.3	N
GL50803_32838	Nfu	39.1	46.4	N

Accession number	Identification	PsortII %mito	TargetP	SignalP
GL50803_3287	Acetyl-CoA acetyltransferase	21.7	80.8	N
GL50803_34058	Mismatch repair protein	4.3	40.1	N
GL50803_34134	Ribonuclease H	8.7	16	N
GL50803_34684	DEAD box RNA helicase Vasa	17.4	18.1	N
GL50803_35428	Valine-tRNA ligase	ND	9.7	N
GL50803_3565	Dihydrouridine synthase, putative	4.3	10	N
GL50803_3595	Endonuclease III	17.4	5.5	N
GL50803_4156	HIT family protein	8.7	5.1	N
GL50803_4849	Adenylate kinase	26.1	46.1	N
GL50803_5593	Ribosomal protein L11	8.7	8.8	N
GL50803_5659	S-adenosylmethionine synthetase	13	18.8	N
GL50803_5867	Cysteinyl-tRNA synthetase	13	14.6	N
GL50803_5919	Cation efflux family protein	13	11.8	N
GL50803_6022	Ribosomal protein S20	8.7	9.7	N
GL50803_6055	FtsJ-like protein	13	11.9	N
GL50803_6184	Branched-chain amino acid aminotransferase lateral transfer candidate	8.7	17.4	N
GL50803_6483	Ribonuclease HI, large sub	4.3	5.1	N
GL50803_7203	Guanylate kinase	65.2	64.2	N
GL50803_7259	CDP-diacylglycerol-glycerol-3-phosphate 3-phosphatidyltransferase	21.7	56.7	N
GL50803_7615	DNA topoisomerase III	21.7	14.9	N
GL50803_7870	Ribosomal protein L23A	17.4	34	N
GL50803_7878	Ribosomal protein S14	43.5	12.6	N
GL50803_7999	Ribosomal protein S3	47.8	9	N
GL50803_8118	Ribosomal protein S2	8.7	16.3	N
GL50803_8444	MDR-type permease	ND	15.5	N
GL50803_87446	ABC transporter family protein	ND	30	N
GL50803_89347	DNA-directed RNA polymerase II largest subunit RPB1	4.3	49.6	N
GL50803_89746	Pseudouridylate synthase, putative	26.1	11	Y
GL50803_9046	Sugar transport family protein	4.3	15.4	N
GL50803_95064	tRNA-ribosyltransferase, putative	22.2	81	N
GL50803_95789	Deoxyribonuclease, TatD family	4.3	8.4	N
GL50803_96460	Alanyl-tRNA synthetase	17.4	27	N
GL50803_98054	Heat shock protein HSP 90-alpha	13	5.8	N
GL50803_9827	Thioredoxin reductase	13	54	N
GL50803_9834	Endonuclease/Exonuclease/phosphatase	17.4	16.3	N
GL50803_9909	Pyruvate, phosphate dikinase	13	25.7	N
