# Supplementary material for: The Minimal Proteome in the Reduced Mitochondrion of the Parasitic Protist Giardia intestinalis
Source: PLoS One. 2011 Feb 24;6(2):e17285. doi: 10.1371/journal.pone.0017285 (PMC3044749; doi:10.1371/journal.pone.0017285)
Supplement: Table S6 — List of primers that were used for subcloning of genes into expression vector pONDRA to investigate subcellular localization of corresponding gene products in G. intestinalis. (PDF) [file pone.0017285.s015.pdf]

Table S6. List of primers that were used for subcloning of genes into expression vector pONDRA to investigate subcellular localization of corresponding gene products in *G. intestinalis*.

Gene ID	Primer sequence
GL50803_10013, Pescadillo	F: CTAGCATATGCCGCTTGTTAGGCGTAAG
	R: AGTCAGATCTCTGTTCATCCTCGTTGGGCAG
GL50803_14939 Hypothetical protein	F: CTAGCATATGCCGGCTCACAGACACAGC
	R: AGTCAGATCTATTTAGCCTTGTCTTGGTCAT
GL50803_14200 Molybdenum cofactor sulfurase	F:CTAGATTAATGACGTCTGTTTTTATTTTT
	R: AGTCAGATCTCTTCGTCTGTAAATCCTCATA
GL50803_15985 Hypothetical protein	F: CTAGCATATGAACACTCTCTTCTCCGTT
	R: AGTCGGATCCAAATTTGTGCAATAGCCTACC
GL50803_3470 ABC transporter	F: CTAGCATATGGTTGCCAATTCCCTTGAT
	R: AGTCGGATCCAAGTGTGAGAACTACTTTGAA
GL50803_6497 Metal dependent	F: CTAGATTAATGTTTCCGTTTATCCCAAAG
	R: AGTCGGATCCTTCGATGGGATATACTTTATC
GL50803_7909 Amino acid	F: CTAGCATATGACTTTAGATTCCGGAGAG
	R: AGTCAGATCTTGTTTTGCGAAACAATAACAC
GL50803_87446 ABC transporter	F: CTAGCATATGGCGAGAAAGTGTCGCAAA
	R: AGTCGGATCCCAGGGACACCGGGGACCCGTC
GL50803_91252 Nitric oxide s Forward	CTAGATTAATGGCCCTGAGCATAGTGTAT
91252 Nitric oxide s Revers	AGTCGGATCCATATGCCTCAACGTGAACTCT
GL50803_9751 DnaJ protein, Type III Forward	CTAGCATATGGGAACCAAATCCAAAAAG
9751 DnaJ protein, Type III Revers	AGTCAGATCTCCTATAGCGCATCGGCCGAAC
GL50803_16596 Hypothetical protein Forward	CTAGCATATGTCTAATAGGTTTACCACC
16596 Hypothetical protein Revers	AGTCGGATCCCTCTTTAAGAGCATAAGTGAT
GL50803_4768 Hypothetical protein Forward	CTAGATTAATGAATCCTGCCATAATACCA
4768 Hypothetical protein Revers	AGTCGGATCCAAAGTTCACAAAGAGATTGTT
GL50803_9296 Hypothetical protein Forward	CTAGCATATGCGTTCTTCTATCGCCGCC
9296 Hypothetical protein Revers	AGTCGGATCCAAGTTTTTCTGCTGAAGGAGA
GL50803_21662 Coiled-coil protei Forward	CTAGATTAATGCCGCACCTGTCCACCCGT
21662 Coiled-coil protei Revers	AGTCGGATCCTTCACACAATTCTGTCGGTTT
GL50803_17296 Major facilitator superfamily mfs1 Forward	CTAGCATATGGTGGTTTCCCCCCATCAT
17296 Major facilitator superfamily mfs1 Revers	AGTCGGATCCCTCGGGTCTGAGGCTCAGCTT
GL50803_9062 Long chain fatty acid CoA ligase 5 Forward	CTAGCATATGTCGGATTTCATCTGCCCC
9062 Long chain fatty acid CoA ligase 5 Revers	AGTCGGATTCCTTACTAGATGGTCTAGAAAA

GL50803_114777 Major facilitator superfamily mfs1 Forward	CTAGATTAATGGCAAATCTCGTTCTGAAC
114777 Major facilitator superfamily mfs1 Revers	AGTCGGATCCGACAGATGCCTGACGCCTCTT
GL50803_16906 Phosphatidate cytidylyltransferase Forward	CTAGCATATGGGGGCTAAACACAGACGT
16909 Phosphatidate cytidylyltransferase Revers	AGTCGGATTCTTCGTAAAAGAAATCATAGAT
GL50803_9719 NADH oxidase Forward	CTAGCATATGTCTTTGCCCTCCACCGTG
9719 NADH oxidase Revers	AGTCGGATCCCAGTTTCATCAGCGTGGAGAT
GL50803_5744 Sec61-alpha Forward	CTAGCATATGTTACAGTTTGTTAAAAGT
5744 Sec61-alpha Revers	AGTCAGATCTCATAGAAAGCTGGATACCTTC
GL50803_12999 Hypothetical protein Forward	CTAGCATATGGAATCTCTCACTGCAGCT
12999 Hypothetical protein Revers	AGTCGGATCCGTCTGTCTTGGCATCAGCCTC
GL50803_29500 cpn10 Forward	GATCCATATGAGCCTCCTTGTCTTGGGT
29500 cpn10 Revers	GATCAGATCTATGAGATGATGCAGTGAAAGT
GL50803_19230 Pam 16 Forward	CATGATTAATATGCTTCTTCCCAAGGCTGGG
19230 Pam 16 Revers	GATCGGATCCTATGTTTTTATTTAAAATCTG
GL50803_17342 major facilitator superfamily mfs1 Forward	GGGCCCATGGCAAATCTCGTTCTA
17342 major facilitator superfamily mfs1 Revers	GATATCCACACGGGCCAAGGAGGC
GL50803_3287 Acetyl-CoA acetyltransferase Forward	CATGCATATGCCCGGGTGTATCTTTGCT
3287 Acetyl-CoA acetyltransferase Revers	AGTCGGATCCCCGGATTCTACGGAGACAGAC
GL50803_7203 Guanylate kinase Forward	CATGCATATGCAGACGCGCTGCTTCAGG
7203 Guanylate kinase Revers	AGTCGGATCCCCCCGAGAGGAATCTCCGCAC
GL50803_7259 CDP-diacylglycerol-glycerol-3-phosphate 3-phosphatidyltransferase Forward	CATGCATATGTCCACTCAGCGCCACGTC
7259 CDP-diacylglycerol-glycerol-3-phosphate 3-phosphatidyltransferase Revers	AGTCGGATCCATCAAATTTTGGTGGAGGTGG
9827Thioredoxin reductase Forward	CATGCATATGTCCACTCAGCGCCACGTC
9827Thioredoxin reductase Revers	AGTCGGATCCCTCCTGCATGGCAAGCCAGCG
15380 Thymidylate kinase, CDC8 Forward	CATGCATATGAGCACGCACAGAAGCACC
15380 Thymidylate kinase, CDC8 Revers	AGTCGGATCCACACAAATTCAATTCTAAATG
17315 ABC transporter, putative Forward	CATGCATATGCTTGAGGAGGAGGACTG
17315 ABC transporter, putative Revers	CATGGGATCCCAGATCCCCCTTTGCATCAAG
9089 Cytochrome B5 Forward	CATATGAGTGAACATCATGGT

9089 Cytochrome B5 Revers	GGATCCGGCCGGCGACCCTCTTCT
17116 Cytochrome B5 Forward	CATATGAATCGACACGTATAC
17116Cytochrome B5 Revers	GGATCCAATAAGCTCATCTTTCC
33870 Cytochrome B5 Forward	CATATGGCATCGCATGGCAGA
33870 Cytochrome B5 Revers	GGATCCGTACCGCCTCCGTAGAAC
2972 Cytochrome B5 Forward	CATATGGAGTCCGTATCTATG
2972 Cytochrome B5 Revers	GGATCCTTCTACAACATGAAAATAC
15398 Chaperone protein dnaJ Forward	CATGGGATCCATGATTTTGTTCCTTCTT
15398 Chaperone protein dnaJ Revers	CTAGCTGCAGTTTACTGACAAGCCTACT
9808 Chaperone protein dnaJ Forward	CTAGATTAATGGTCAAGGAGACAGAG
9808 Chaperone protein dnaJ Revers	CATGCTCGAGTCAGGCAGAGTCTGGGACGT<br>CATATGGATACATCGCCTGGCACTGCGCACC
17483 Chaperone protein dnaJ Forward	CATGAGATCTATGGGTAGGAGTTTCTAT
17483 Chaperone protein dnaJ Revers	CTAGCTGCAGATTTGGCGGCAGCAGCTC
